# A Risk Model Based on Sorafenib-Response Target Genes Predicts the Prognosis of Patients with HCC

**DOI:** 10.1155/2022/7257738

**Published:** 2022-06-28

**Authors:** Xiang Liu, Jian Zeng, Huanyu Li, Feng Li, Bin Jiang, Ming Zhao, Zhuo Liu, Ruineng Li, Tiexiang Ma

**Affiliations:** Third Department of General Surgery, The Central Hospital of Xiangtan, Xiangtan, Hunan, China

## Abstract

Sorafenib is used to treat digestive system tumors in patients who do not respond to or cannot tolerate surgery. However, the roles and inhibitory mechanisms of sorafenib against hepatocellular carcinoma (HCC) are unclear. Differentially expressed genes in tissues from responders and nonresponders to sorafenib were investigated using the HCC GSE109211 data set. Biological functions and mechanisms were studied using the Gene Ontology and Kyoto Encyclopedia of Genes and Genomes databases. The expression levels of differential expressed target genes were identified in HCC tissues, using The Cancer Genome Atlas database, and their prognostic and diagnostic values were explored using survival and receiver operating characteristic curve analysis. A nomogram and risk model of sorafenib-response target genes enabled the evaluation of the prognosis of patients with HCC. The relationship between risk scores and levels of infiltrating immune cells was visualized via correlation analysis. We identified 1620 sorafenib-response target genes involved in the PPAR signaling pathway, antigen processing and presentation, and ferroptosis. SLC41A3, SEC61A1, LRP4, PPM1G, and HSP90AA1 were independent risk factors for a poor prognosis for patients with HCC and had diagnostic value. A risk model based on SLC41A3, SEC61A1, LRP4, PPM1G, and HSP90AA1 expression showed that patients with HCC in the high-risk group had a worse prognosis. Consensus-clustering analysis (performed with K set to 2) distinguished two clusters (the cluster 1 and cluster 2 groups). Patients in cluster 1 survived significantly longer than those in cluster 2. The risk score correlated with the levels of T cells, cytotoxic lymphocytes, CD8^+^ T cells, macrophages, memory B cells, follicular helper T cells, and other immune cells. The high risk based on the sorafenib-response targets SLC41A3, SEC61A1, LRP4, PPM1G, and HSP90AA1 represented the poor prognosis for patients with HCC and significantly correlated with the levels of immune infiltrating cells in HCC.

## 1. Introduction

Liver cancer has high morbidity and mortality rates and is one of the most common cancers in the world. Hepatocellular carcinoma (HCC) is the most common type of liver cancer [1, 2]. Ultrasonography and alpha-fetoprotein (AFP) screening are often used for the early diagnosis of HCC. However, ultrasonography and AFP have certain limitations in HCC [1]. Previous data have shown that some genes, microRNAs, and long noncoding RNAs (lncRNAs) have important biological roles in the progression of HCC [2–7]. For example, Cheng et al. reported that kinesin family members 14 (KIF14) and 23 (KIF23) are upregulated in HCC tissues. KIF14 and KIF23 upregulation were associated with shorter overall survival (OS), recurrence-free survival (RFS), and disease-specific survival (DFS) in patients with HCC. Knocking down KIF14 and KIF23 inhibited cell proliferation, promoted cell invasion and HCC migration, and upregulated Bax expression. Decreased expression of KIF14 and KIF23 was shown to enhance the chemosensitivity of HCC cells to cisplatin and sorafenib [4]. He et al. reported that the expression level of lncRNA ZFPM2-AS1 was significantly higher in HCC tissues than in the adjacent normal tissues. High ZFPM2-AS1 expression levels were associated with a shorter OS. ZFPM2-AS1 silencing can inhibit the proliferation, migration, and invasion of HCC cells and promote apoptosis in vitro. ZFPM2-AS1 can regulate GDF10 expression by competitively binding to miR-139, and miR-139 overexpression and GDF10 downregulation can reverse the cellular phenotype induced by ZFPM2-AS1 [6]. Currently, biomarkers for diagnosing HCC and evaluating the prognosis of patients with HCC are lacking.

The results of previous studies have shown that sorafenib has anticancer efficacy, and it is often used in patients with advanced cancer that cannot be treated surgically [8–14]. For example, sorafenib can inhibit breast cancer (BC) cell proliferation, migration, and invasion and exerts cytotoxic effects. Sorafenib has been found to promote mitochondrial superoxide production and inhibit BC stem cell self-renewal, the epithelial-mesenchymal transition (EMT), and the ERK signaling pathway [12]. Sorafenib can also inhibit HCC cell viability, proliferation, and migration in dose-dependent manners. Sorafenib can destroy the mitochondrial morphology of HCC cells, reduce oxidative-phosphorylation activity, lower the mitochondrial membrane potential, reduce ATP synthesis, and lead to cell death [11]. Furthermore, the OS of sorafenib-treated patients with HCC was compared to that of patients with HCC who did not receive sorafenib. Among patients with grade 1/2, the median OS was 6.1 months in sorafenib-treated patients and 3.1 months in sorafenib-naïve patients [13, 14]. However, the signaling mechanisms of sorafenib against HCC have not been fully elucidated. Using microarrays, Pinyol et al. analyzed gene expression levels in tissues from patients with HCC who were treated with sorafenib in order to explore potential biomarkers and improve patient survival [15]. In this study, the data from Pinyol et al. were used to explore the target genes in response to sorafenib treatment. The biological functions and signaling mechanisms of the response target genes were investigated. The response target genes with prognostic and diagnostic significance for patients with HCC were screened and identified, and sorafenib-response target-gene nomogram and risk model were constructed to assess their utilities in determining patient prognosis.

## 2. Materials and Methods

### 2.1. GSE109211 Data Set

The data for HCC patients treated with sorafenib in the GSE109211 data set of the Gene Expression Omnibus (GEO; http://www.ncbi.nlm.nih.gov/geo) database were analyzed using GEO2R. The patients were grouped based on the effectiveness of sorafenib treatment, with 46 patients in the nonresponder group and 21 patients in the responder group. In addition, the platform-annotation file (GPL13938 : Illumina HumanHT-12 WG-DASL V4.0 expression beadchip) in the GSE109211 data set was downloaded.

### 2.2. Biological Functions and Signaling Mechanisms

The Gene Ontology (GO) and the Kyoto Encyclopedia of Genes and Genomes (KEGG) databases are often used to investigate the biological functions and signaling mechanisms involving multiple genes. In this study, the R clusterProfiler package was used to investigate the biological processes, cellular components, and molecular functions associated with sorafenib-response targets based on GO annotation. The KEGG database was used to analyze possible signaling mechanisms involved in the response to sorafenib [16].

### 2.3. Protein-Protein Interaction (PPI) Network

The sorafenib-response targets were entered into the Search Tool for the Retrieval of Interacting Genes (STRING; https://string-db.org/) website, and human species were selected to explore the PPI relationships between the sorafenib-response targets [16]. Cytoscape software (version 3.8.2) was used to visualize the PPI network, and the CytoHubba plugin was used to explore key genes in the PPI network of sorafenib-response target genes based on the connectivity scores. The expression levels of key genes in the PPI network and their prognostic value in HCC were visualized using the Gene Expression Profiling Interactive Analysis (GEPIA) online database.

### 2.4. Identifying the Expression Levels of Sorafenib-Response Targets Using The Cancer Genome Atlas (TCGA) Database

Published data for 374 HCC tissues and 50 normal liver tissues were downloaded from the official website of the TCGA (https://portal.gdc.cancer.gov/projects/) database. The sorafenib-responsive target genes in the GEO database were merged with the gene-expression data in the TCGA database, and then, the expression levels of sorafenib-responsive target genes in normal and HCC tissues were analyzed using the Limma package. Aberrantly expressed sorafenib-responsive genes were visualized using heat maps and dot plots.

### 2.5. Prognostic and Diagnostic Values of the Sorafenib-Response Target Genes

The differentially expressed sorafenib-response gene data in the TCGA database were merged with the prognostic data for the patients with HCC. Patients with incomplete prognostic information were excluded. The influence of the sorafenib-response target genes in the OS of patients with HCC was explored using Kaplan–Meier (K-M) survival analysis, where *P* < 0.001 was used as the screening criterion. A receiver operating characteristic (ROC) curve was used to evaluate the impact of the prognosis-related response genes on the disease diagnosis. The closer the area under the curve (AUC) was to 1, the greater the diagnostic value [17].

### 2.6. Risk Models of Sorafenib-Response Target Genes

The relationships between the OS of patients and the expression levels of BAMBI, BRD9, CCT3, CDC123, DEGS1, DENR, DHX37, EIF3B, GAPDH, HM13, HSP90AA1, IQCA1, LRP4, MCM8, PIGU, PPFIA4, PPM1G, RRP7A, SEC61A1, SLC25A39, SLC41A3, SOX11, SPC25, TAGLN2, ZC3H3, and ZNF207 expression levels were explored using univariate Cox regression analysis. On this basis, multivariate Cox regression analysis and the Akaike information criterion (AIC) method were performed to screen independent prognostic factors and construct a risk model [18, 19].

### 2.7. Verifying the Values of Risk Model and Constructing the Nomogram of Prognostic Genes

After grouping patients based on the median expression of risk model factors, the relationships between risk-model genes and the clinicopathological characteristics of patients were explored. The relationships between the high- and low-risk models and the prognosis of patients with HCC were evaluated by performing K-M survival analysis, and the AUC of the risk model at 3 and 5 years was analyzed via ROC analysis. COX regression analysis was used to explore the effectiveness of the risk model in assessing the prognosis of patients with HCC [17]. The roles of risk-model factors in patient prognosis were incorporated into the nomogram.

### 2.8. Consensus Clustering and Prognostic Assessment of Sorafenib-Response Target Genes

Principal component analysis (PCA) was performed by dividing the data from 374 patients with HCC (from the TCGA database) into two groups according to the gene-expression levels of SLC41A3, SEC61A1, LRP4, PPM1G, and HSP90AA1 using the R Consensus ClusterPlus package [18]. K-M survival analysis consensus clustering was used to separate the patients into two groups based on their survival rates.

### 2.9. Risk Model and Immune Infiltration

The MCPcounter and CIBERSORT algorithms were separately applied to TCGA gene-expression data obtained from patients with HCC in order to calculate immune cell levels [20]. The TCGA HCC risk-model score was merged with patient immune cell data. The patients were divided into two risk groups, and the expression levels of immune cells in both groups were investigated.

### 2.10. Statistical Analysis

Gene-expression levels in TCGA HCC tissues were analyzed using the Limma package. COX regression analysis, K-M survival analysis, and ROC analysis were used to explore the value of sorafenib-response targets in the prognosis and diagnosis of patients with HCC. Immune cell levels in the risk models were identified using the *t*-test. The threshold for statistical significance was set at *P* < 0.05.

## 3. Results

### 3.1. Sorafenib-Response Targets in HCC

**Figure 1:**
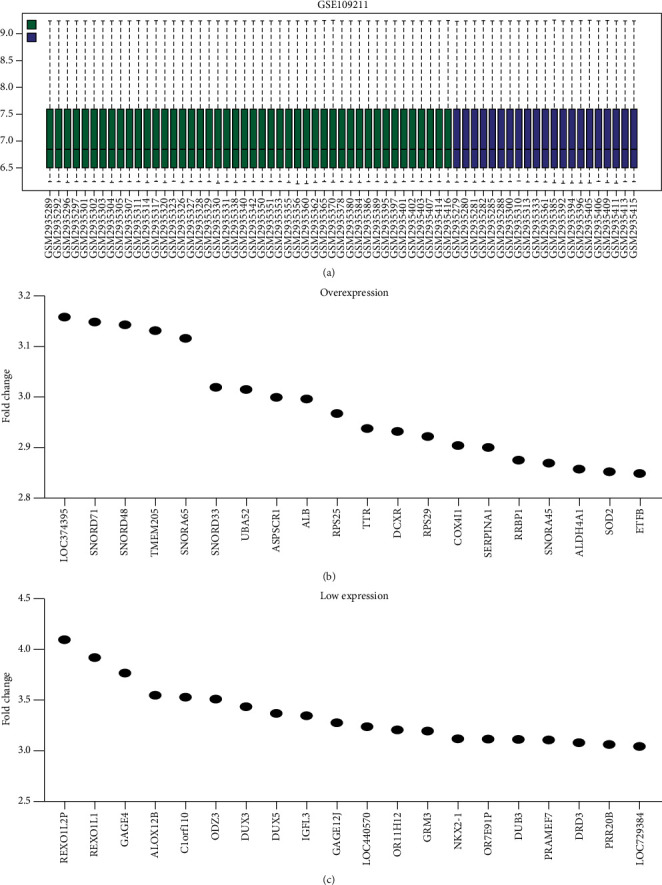
Tissue samples and DEGs in sorafenib-treated HCC patients based on the GSE109211 data set. Abbreviations: DEGs, differentially expressed genes; HCC, hepatocellular carcinoma.

Sixty-five samples from sorafenib-treated patients with HCC were included in the GSE109211 data set (Figure 1(a)). The gene-expression levels in the nonresponder and responder groups were analyzed using GEO2R, and the results showed that the tissue samples were consistent (Figure 1(a)). Compared with the tissues of the nonresponder group, those from the responder group had 1620 differentially expressed genes (DEGs) (Table S1). Among them, 799 DEGs were upregulated and 821 genes were downregulated. Figures 1(b) and 1(c) show the top 20 DEGs according to the fold changes in expression.

### 3.2. Functions and Mechanisms of Sorafenib-Response Targets

**Figure 2:**
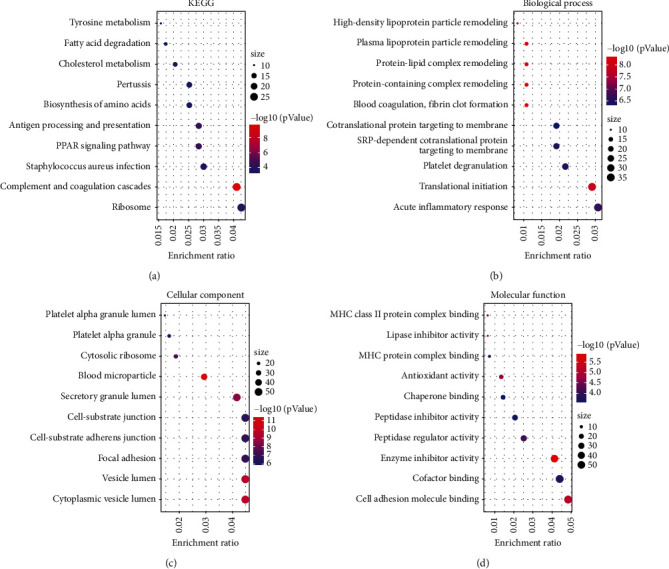
Functions and mechanisms of sorafenib-response target genes based on the GO and KEGG databases. Abbreviations: GO, Gene Ontology; KEGG, Kyoto Encyclopedia of Genes and Genomes.

KEGG pathway indicated that the sorafenib-response targets were enriched for terms such as complement and coagulation cascades, PPAR signaling pathway, antigen processing and presentation, ribosomes, cholesterol metabolism, amino acid biosynthesis, ferroptosis, and other terms (Figure 2(a) and Table 1). GO annotation showed that the sorafenib-response targets were enriched for terms such as translational initiation, protein targeting to ER, protein localization to the endoplasmic reticulum, response to toxic substance, mRNA catabolic process, protein activation cascade, protein targeting, drug catabolic process, RNA catabolic process, protein targeting to membrane, regulation of production of molecular mediator of the immune response, humoral immune response, epithelial tube formation, epidermal cell differentiation, positive regulation of cytokine production involved in immune response, and other terms (Figure 2(b)–2(d) and Table S2).

### 3.3. PPI Network of Sorafenib-Responsive Targets

**Figure 3:**
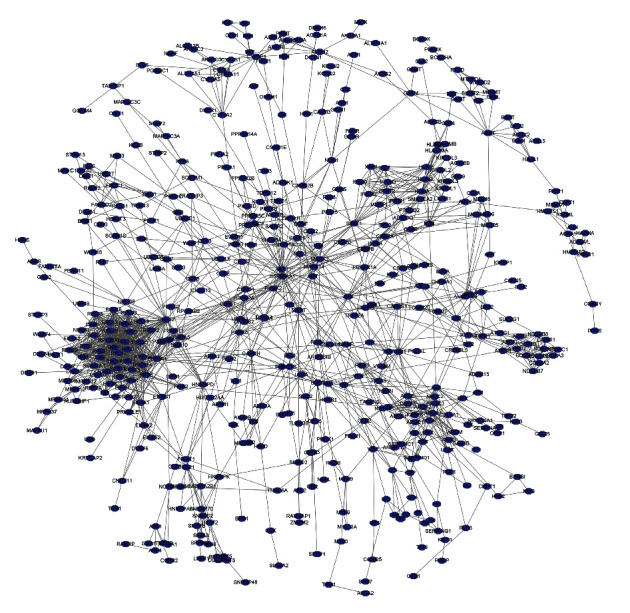
PPI network of sorafenib-response target genes. Abbreviation: PPI, protein-protein interaction.

The PPI network revealed protein-interaction relationships between sorafenib-responsive target genes (Figure 3). The top 10 key response target genes were screened based on the binding score, using the CytoHubba plugin. The key response genes identified were UBA52, RPS11, RPS16, RPS6, RPL11, RPS14, RPL5, FAU, RPL9, and RPL13A (Figure S1). Most of the response target genes have important clinical value for HCC in the GEPIA database (Figure S2). In detail, UBA52, RPS11, RPS16, RPS6, RPS14, RPL5, and FAU were significantly overexpressed in HCC tissues. Overexpression of UBA52, RPS11, RPS16, RPS6, RPS14, and RPL5 was significantly associated with a poor prognosis for patients with HCC.

### 3.4. Determining the Expression Levels of Sorafenib-Response Target Genes

**Figure 4:**
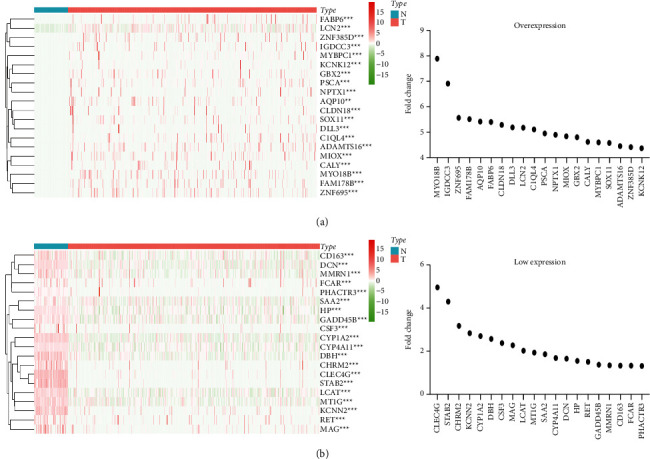
DEGs in sorafenib-response target genes were identified in HCC tissues (based on information in the TCGA database) based on fold-change differences in expression. Abbreviations: HCC, hepatocellular carcinoma; TCGA, The Cancer Genome Atlas.

In the TCGA database, 393 sorafenib-responsive target genes were abnormally expressed in HCC tissues versus normal liver tissues (Table S3). Among them, 352 response target genes were upregulated in HCC tissues and 41 were downregulated.  Figure 4 shows a heat map with the top 20 sorafenib-responsive target genes based on fold changes in expression.

### 3.5. Prognostic Values of Identifying Sorafenib-Response Targets

**Figure 5:**
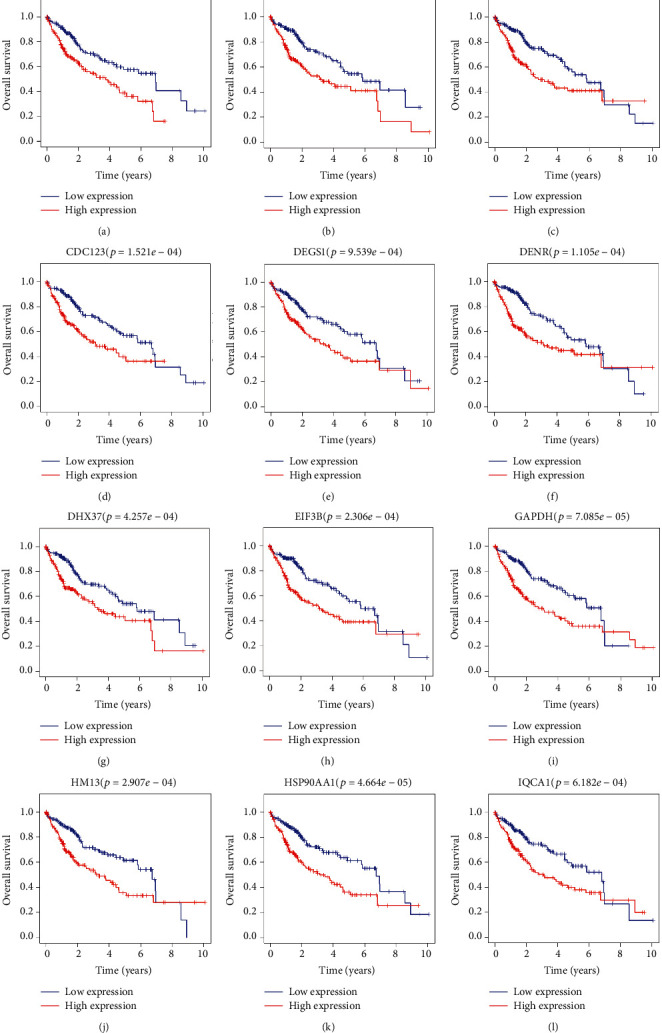
The prognostic values of sorafenib-response target genes in the TCGA database were determined by performing K-M survival analysis. Abbreviations: HCC, hepatocellular carcinoma; K-M, Kaplan–Meier; TCGA, The Cancer Genome Atlas.

K-M survival analysis showed that 26 sorafenib-responsive genes were associated with a poor prognosis for patients with HCC (*P* < 0.001). Specifically, the overexpression of BAMBI, BRD9, CCT3, CDC123, DEGS1, DENR, DHX37, EIF3B, GAPDH, HM13, HSP90AA1, IQCA1, LRP4, MCM8, PIGU, PPFIA4, PPM1G, RRP7A, SEC61A1, SLC25A39, SLC41A3, SOX11, SPC25, TAGLN2, ZC3H3, and ZNF207 was significantly associated with a poor prognosis for patients with HCC (Figures 5 and S3).

### 3.6. Diagnostic Values of Sorafenib-Response Target Genes

**Figure 6:**
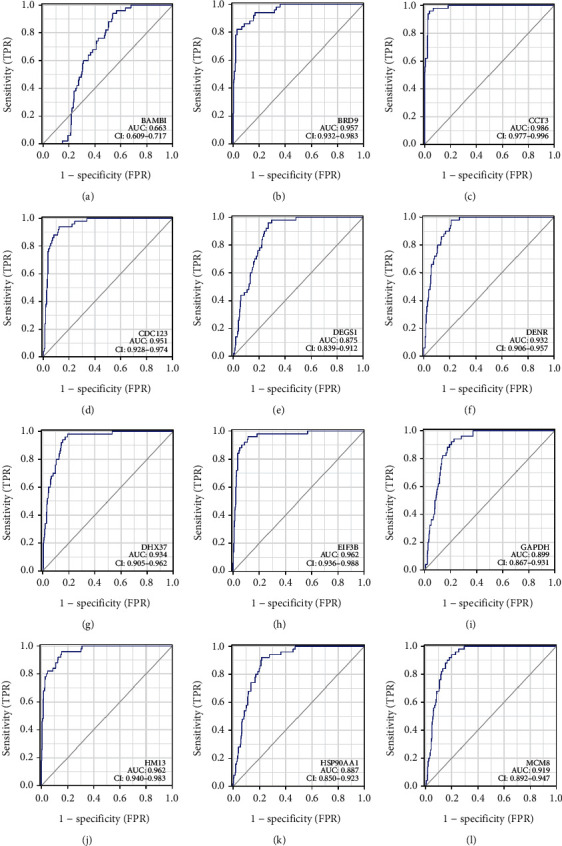
The diagnostic values of sorafenib-response target genes in the TCGA database were determined by performing ROC analysis. Abbreviations: ROC, receiver operating characteristic; TCGA, The Cancer Genome Atlas.

BAMBI, BRD9, CCT3, CDC123, DEGS1, DENR, DHX37, EIF3B, GAPDH, HM13, HSP90AA1, IQCA1, LRP4, MCM8, PIGU, PPFIA4, PPM1G, RRP7A, SEC61A1, SLC25A39, SLC41A3, SOX11, SPC25, TAGLN2, ZC3H3, and ZNF207 had significant diagnostic values for HCC (Figures 6 and S4). The respective AUC values were 0.663, 0.957, 0.986, 0.951, 0.875, 0.932, 0.934, 0.962, 0.899, 0.962, 0.887, 0.919, 0.589, 0.640, 0.977, 0.746, 0.971, 0.873, 0.926, 0.977, 0.974, 0.744, 0.969, 0.903, 0.962, and 0.941 based on ROC analysis.

### 3.7. Construction of a Risk Model for Sorafenib-Response Target Genes

Univariate Cox regression analysis revealed that overexpression of SOX11, PPFIA4, SLC41A3, SPC25, PIGU, GAPDH, ZC3H3, TAGLN2, DHX37, MCM8, BRD9, CDC123, CCT3, RRP7A, HM13, SLC25A39, DENR, EIF3B, SEC61A1, LRP4, PPM1G, DEGS1, BAMBI, HSP90AA1, and ZNF207 was associated with a poor prognosis for patients with HCC (Figure S5). A risk model was constructed based on the multivariate Cox regression analysis and AIC standardization. The results revealed SLC41A3, SEC61A1, LRP4, PPM1G, and HSP90AA1 as independent influencing factors of a dismal prognosis for patients with HCC (Table 2) and key risk-model genes. The risk score was calculated using the following formula: risk score = SLC41A3 *∗* 0.402806964 + SEC61A1 *∗* 0.352569453 + LRP4 *∗*0.318687138+ PPM1G *∗* 0.43460103+ HSP90AA1 *∗* 0.332298087.

### 3.8. Prognostic Value of the Risk Model

UHSP90AA1-expression levels correlated with the age, fibrosis Ishak score (FIS), and OS of patients with HCC (Table S4). LRP4-expression levels correlated with the T stage, pathologic stage, sex, adjacent hepatic tissue inflammation, vascular invasion, and OS in patients with HCC (Table S5). PPM1G-expression levels correlated with the T stage, pathologic stage, race, weight, body-mass index (BMI), histologic grade, AFP level, and OS of patients with HCC (Table S6). SEC61A1-expression levels correlated with the T stage, pathologic stage, sex, OS, and disease-specific survival (DSS) of patients with HCC (Table S7). SLC41A3-expression levels correlated with the weight, BMI, histologic grade, AFP, and OS of patients with HCC (Table S8).

**Figure 7:**
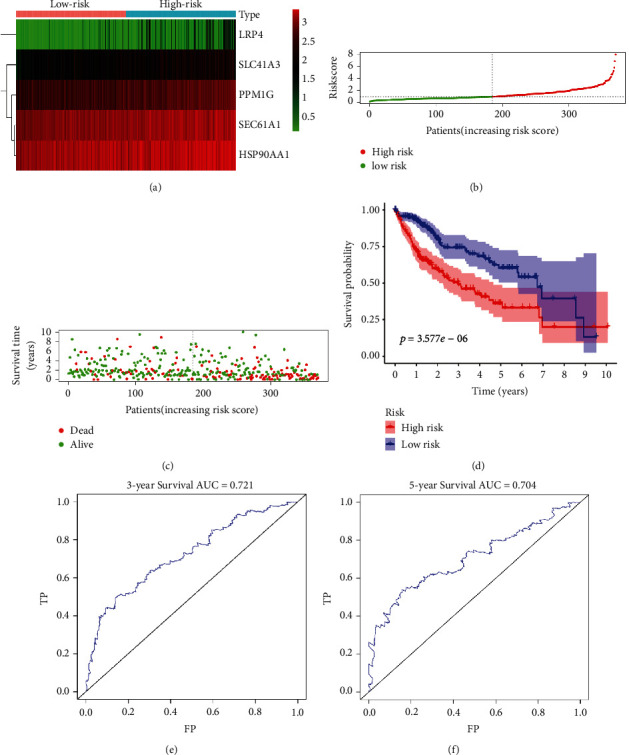
Risk model associated with the prognosis of patients with HCC. (a) Sorafenib-responsive target genes used to construct the risk model. (b)–(d) The risk score correlated with a dismal prognosis of patients with HCC. (e)–(f) The 3- and 5-year diagnostic value of the risk model based on ROC analysis. Abbreviations: HCC, hepatocellular carcinoma; ROC, receiver operating characteristic.

**Figure 8:**
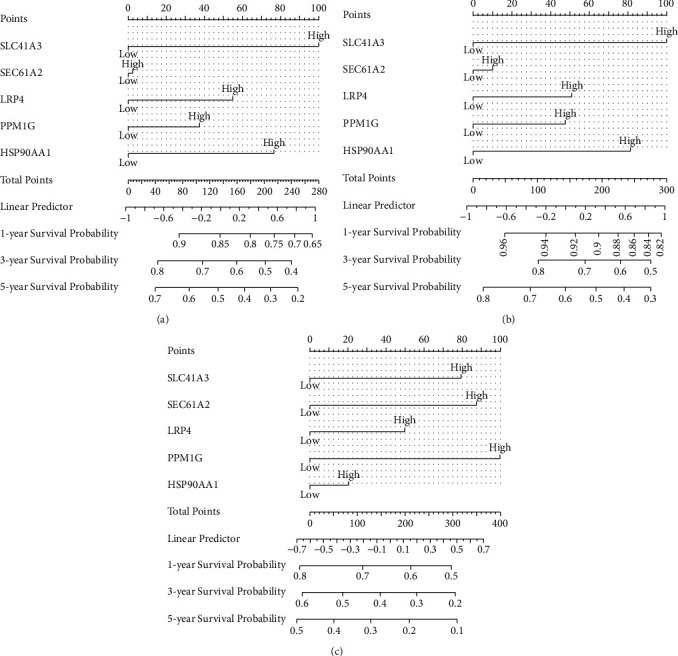
Construction of prognosis-related nomogram based on the sorafenib-responsive target genes. (a) OS; (b) DSS; (c) DFI. Abbreviations: DFI, disease-free survival; DSS, disease-specific survival; OS, overall survival.

Figure 7(a) shows the relationships between different sorafenib-response targets (SLC41A3, SEC61A1, LRP4, PPM1G, and HSP90AA1) and the risk models. Figures 7(b) and 7(c) show the relationships between the risk scores and survival times of patients with HCC. K-M survival analysis showed that HCC patients in the high-risk group had a worse prognosis (Figure 7(d)). Time-dependent diagnostic analysis showed that the risk model had a significant diagnostic value (Figures 7(e) and 7(f)). Univariate and multivariate Cox regression analyses showed that the risk score was an independent risk factor for a poor prognosis for patients with HCC (Figure S6). Therefore, we constructed prognostic nomograms of the sorafenib-responsive targets, SLC41A3, SEC61A1, LRP4, PPM1G, and HSP90AA1 (Figure 8).

### 3.9. Consensus Clustering of Sorafenib-Response Targets Correlated with Distinct Clinical Outcomes in Patients with HCC

**Figure 9:**
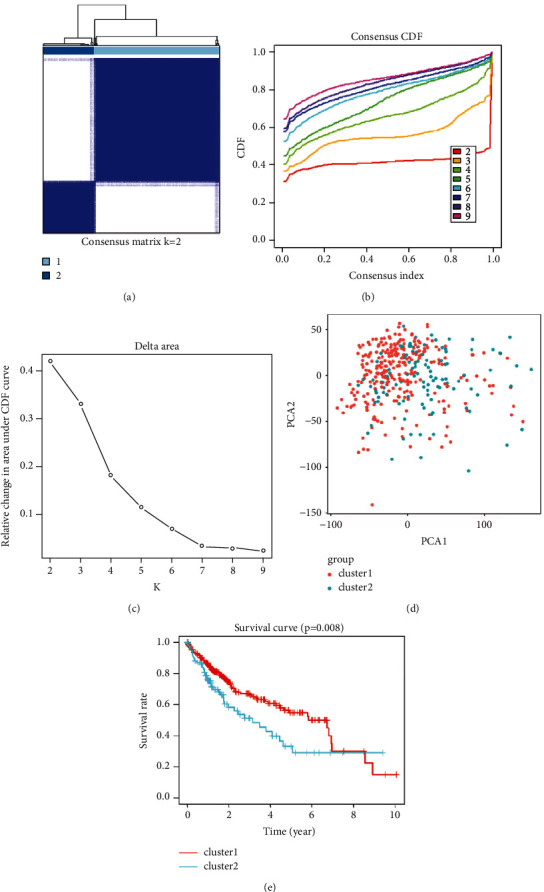
Consensus clustering of sorafenib-response targets identified distinct clinical outcomes in patients with HCC. Abbreviation: HCC, hepatocellular carcinoma.

Consensus-clustering analysis with the TCGA HCC data for the sorafenib-response targets, SLC41A3, SEC61A1, LRP4, PPM1G, and HSP90AA1, showed that *K* = 2 enabled the best grouping (Figures 9(a)–9(c)). Consensus-clustering analysis was performed with *K* = 2, and the patients were grouped into two clusters, namely, the cluster 1 and cluster 2 groups, with significant differences. PCA revealed a significant difference between the cluster 1 and cluster 2 groups based on data for 374 patients with HCC in the TCGA database (Figure 9(d)). K-M survival analysis showed that the survival times of patients with HCC in the cluster 1 group were significantly higher than those in the cluster 2 group (Figure 9(e)).

### 3.10. A Risk Model Based on the Sorafenib-Response Target Genes Was Associated with HCC Immune Cell Infiltration

**Figure 10:**
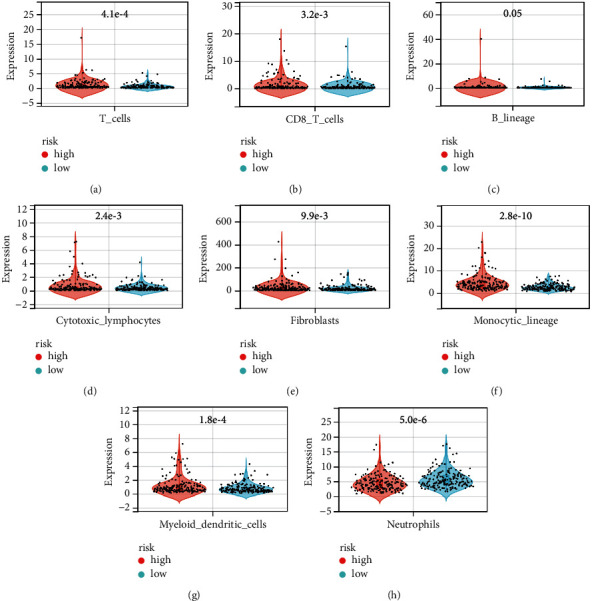
The expression levels of immune cells were abnormal in the high- and low-risk groups. (a) T cells; (b) CD8^+^ T cells; (c) B lineage cells; (d) cytotoxic lymphocytes; (e) fibroblasts; (f) monocytic lineage cells; (g) myeloid dendritic cells; (h) neutrophils.

**Figure 11:**
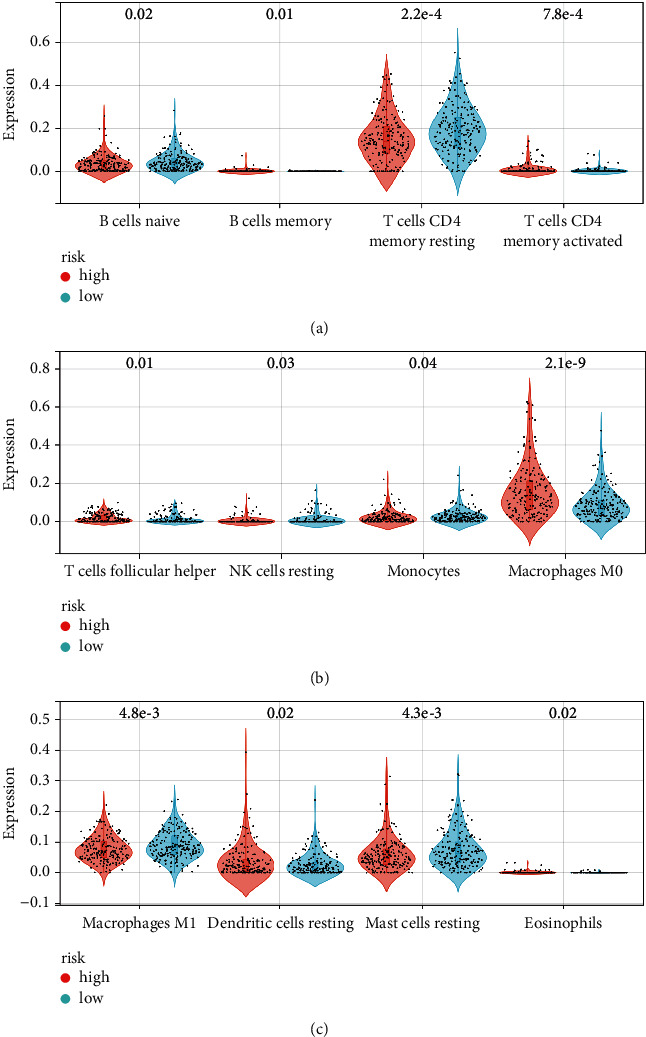
The expression levels of immune cells were abnormal in the high- and low-risk groups.

SLC41A3, SEC61A1, LRP4, PPM1G, and HSP90AA1 were differentially expressed between the high- and low-risk groups and correlated positively with risk scores (Figure S7). The levels of immune cells in HCC tissues were calculated using the MCPcounter algorithm, and the results showed that the abundances of the B lineage, neutrophils, myeloid dendritic cells, T cells, cytotoxic lymphocytes, CD8^+^ T cells, monocytic lineage, and fibroblasts were abnormal in the high- and low-risk groups and that these differences were statistically significant (Figure 10 and Table 3). The levels of immune cells in HCC tissues were calculated via CIBERSORT analysis, and the results showed that the abundances of M0 macrophages, T cells, CD4^+^ memory T cells, activated CD4^+^ memory cells, resting mast cells, M1 macrophages, memory B cells, follicular helper T cells, naïve B cells, resting dendritic cells, eosinophils, neutrophils, resting NK cells, and monocytes differed significantly in the high- and low-risk groups (Figure 11 and Table 4).

## 4. Discussion

Liver cancer is highly malignant and insensitive to cytotoxic chemotherapy, often resulting in a poor prognosis for cancer patients. Sorafenib was developed early as a small-molecule drug used in the first-line treatment of advanced liver cancer [8, 21]. However, some patients with liver cancer do not respond to treatment with sorafenib and, thus, have a poor prognosis. Therefore, in this study, we grouped patients with advanced HCC in terms of their response to sorafenib to explore the key response targets and provide new candidate molecules for improving the prognosis of such patients.

The PPAR signaling pathway, antigen processing and presentation, and ferroptosis are associated with cancer growth and metastasis [22–27]. For example, the overexpression of the lncRNA TINCR inhibits colorectal cancer (CRC) cell proliferation and promotes apoptosis. Overexpression of miR-107 in CRC cells induces cancer cell proliferation and inhibits apoptosis. TINCR overexpression regulates the PPAR signaling pathway through the miR-107/CD36 signaling axis to inhibit CRC progression [23]. Interferon gamma (IFN*γ*) released by CD8 ^+^ T cells or natural killer cells plays a crucial role in antitumor host immunity. IFN*γ* is involved in regulating tumor cell proliferation and apoptosis. IFN*γ* enhances glutathione depletion, promotes cell cycle arrest in the G0/G1 phase, increases lipid peroxidation, and sensitizes HCC cells to ferroptosis activators. IFN*γ* downregulates SLC3A2 and SLC7A1 mRNA and protein expression by activating the JAK/STAT signaling pathway in HCC cells [27]. In this study, we found that sorafenib-response target genes are involved in the PPAR signaling pathway, antigen processing and presentation, ferroptosis, and other mechanisms. Preliminary evidence suggests that the anticancer effects of sorafenib in HCC patients are related to these mechanisms.

The results of previous studies have confirmed that SLC41A3, SEC61A1, LRP4, PPM1G, and HSP90AA1 play important roles in cancer progression [28–39]. SLC41A3 is a member of solute carrier family 41 and is involved in many cellular processes. The expression level of SLC41A3 in HCC tissues was higher than that in the normal tissues. SLC41A3 is associated with HCC metastasis, the disease grade, microvascular invasion, AFP, and prognosis and is an influencing factor for a short OS in patients with HCC [28]. Upregulated SEC61A1 expression promoted HCC cell proliferation, migration, and stem cell properties. miR-491-5p inhibits HCC cell proliferation and migration by regulating SEC61A1 expression. VPS9D1-AS1 can upregulate SEC61A1 expression by sponging miR-491-5p, which in turn is involved in HCC growth and metastasis [30]. PPM1G is overexpressed in HCC tissues and associated with HCC metastasis, the pathological grade, microvascular invasion, and hepatitis B virus (HBV) infection. High PPM1G expression is an independent prognostic factor for patients with HCC, and PPM1G regulates SRSF3 phosphorylation in HCC cells and contributes to the proliferation, invasion, and metastasis of HCC cells. PPM1G knockdown inhibits HCC cell growth, invasion, and tumor growth in vivo [34, 35]. In this study, we found that SLC41A3, SEC61A1, LRP4, PPM1G, and HSP90AA1 were abnormally expressed in responder and nonresponder tissues from patients with HCC who were treated with sorafenib. In addition, compared with normal liver tissue, the expression levels of SLC41A3, SEC61A1, LRP4, PPM1G, and HSP90AA1 in HCC tissues were significantly higher and correlated significantly with a dismal prognosis for patients with HCC, which has significant diagnostic value. HSP90AA1 is associated with the age, FIS, and OS of patients with HCC. LRP4 is associated with the T stage, pathologic stage, sex, adjacent hepatic tissue inflammation, vascular invasion, and OS of patients with HCC. PPM1G is correlated with the T stage, pathologic stage, race, weight, BMI, histologic grade, AFP, and OS of patients with HCC. SEC61A1 is correlated with the T stage, pathologic stage, sex, OS, and DSS of patients with HCC. LC41A3 is correlated with the weight, BMI, histologic grade, AFP level, and OS of patients with HCC. The risk model constructed based on SLC41A3, SEC61A1, LRP4, PPM1G, and HSP90AA1 was associated with the OS of patients with HCC, where overexpression of these genes negatively impacted the prognosis. The prognostic nomogram constructed based on the sorafenib-response targets SLC41A3, SEC61A1, LRP4, PPM1G, and HSP90AA1 is expected to better assess the prognosis of patients with HCC and provide new candidate molecules.

Infiltrating immune cells in the immune microenvironment play crucial roles in the occurrence and development of HCC [39–42]. For example, the immune cell marker, CD39, is expressed in tumor-infiltrating lymphocytes and is a marker for identifying tumor-reactive T cells. Simultaneous knockdown of PD-1, Tim-3, and Lag-3 enhances the antitumor activity of CD39 CAR-T cells. CD39 CAR-T cells showed increased interferon-*γ* secretion and potent antitumor effects in a mouse model of patient-derived xenografts [39]. Amygdalin treatment rescued HBV-T cell viability and promoted IFN*γ* and TNF-*α* production. Among HBV-T cells, the mean fluorescence intensity of CD8^+^ T cells decreased significantly. Amygdalin reduces the phosphorylation of STAT3 and JAK2 in HBV-T cells. Amygdalin suppresses HBV-related HCC cell proliferation, invasion, and migration through T cell-mediated tumor immunity [40]. The risk model constructed here, based on SLC41A3, SEC61A1, LRP4, PPM1G, and HSP90AA1 expression, was associated with the levels of immune infiltrating cells in HCC. The risk score was correlated with the B lineage, monocytic lineage, neutrophils, myeloid dendritic cells, T cells, cytotoxic lymphocytes, CD8^+^ T cells, fibroblasts, M0 macrophages, T cells, resting CD4^+^ memory T cells, activated CD4^+^ memory T cells, resting mast cells, M1 macrophages, memory B cells, follicular helper T cells, naïve B cells, resting dendritic cells, eosinophils, neutrophils, resting NK cells, and monocytes, which further indicates that the risk model constructed based on sorafenib-response target genes has an important value in predicting HCC progression.

In this study, we used information from the GEO and TCGA databases to identify key sorafenib-response targets for patients with advanced HCC and a prognostic gene-expression signature. The data obtained in this study offered the advantages of a large sample size and high reliability. However, a risk model based on sorafenib-response target genes needs to be established through basic experiments and clinical applications. Overall, the sorafenib-response targets SLC41A3, SEC61A1, LRP4, PPM1G, and HSP90AA1 were found to be independent risk factors for a poor prognosis for patients with HCC. Using the risk model constructed based on the expression of these five genes, we found that patients with HCC in the high-risk group had a worse prognosis and that the risk model had excellent diagnostic value. The survival times of patients with HCC in the cluster 1 and cluster 2 groups were significantly different. The risk model constructed based on SLC41A3, SEC61A1, LRP4, PPM1G, and HSP90AA1 is expected to enable the prognosis of patients with HCC and significantly correlated with the levels of infiltrating immune cells in HCC.

## 5. Conclusions

In our study, the high risk based on the sorafenib-response targets SLC41A3, SEC61A1, LRP4, PPM1G, and HSP90AA1 represented the poor prognosis for patients with HCC and significantly correlated with the levels of immune infiltrating cells in HCC.

## Figures and Tables

**Table 1:** The signaling mechanisms of sorafenib-response target genes as determined by performing KEGG analysis.

Type	Description	*P* value	FDR
hsa04610	Complement and coagulation cascades	1.69E-10	5.38E-08
hsa03320	PPAR signaling pathway	2.19E-05	0.003386551
hsa04612	Antigen processing and presentation	3.19E-05	0.003386551
hsa03010	Ribosome	7.06E-05	0.00561395
hsa04979	Cholesterol metabolism	0.000109262	0.006949059
hsa05150	Staphylococcus aureus infection	0.000178779	0.009475262
hsa01230	Biosynthesis of amino acids	0.000232795	0.010575559
hsa05133	Pertussis	0.000273467	0.0108703
hsa00350	Tyrosine metabolism	0.000379378	0.013404691
hsa00071	Fatty acid degradation	0.000530146	0.016858636
hsa04216	Ferroptosis	0.000944308	0.02729909

Abbreviations: FDR, false discovery rate; KEGG, Kyoto Encyclopedia of Genes and Genomes.

**Table 2:** Prognostic factors and risk-model genes of patients with HCC as determined by performing Cox regression analysis.

Gene	Coef.	HR	HR.95L	HR.95H	*P* value
SLC41A3	0.402806964	1.496018079	1.030208213	2.172444428	0.034317063
SEC61A1	0.352569453	1.422718465	1.010657817	2.002782539	0.043308215
LRP4	0.318687138	1.375320972	1.056534063	1.790295117	0.017849829
PPM1G	0.43460103	1.544346787	1.056839953	2.256734327	0.024728555
HSP90AA1	0.332298087	1.39416837	1.064469632	1.825984871	0.015787549

Abbreviations: HCC, hepatocellular carcinoma; HR, hazard ratio.

**Table 3:** The expression levels of immune cells were abnormal in the high- and low-risk groups.

Immune cell	High-risk group	Low-risk group	*t*-test
T cells	1.16 ± 1.61	0.69 ± 0.76	4.10E-04
CD8 T cells	1.30 ± 2.46	0.68 ± 1.45	3.20E-03
Cytotoxic lymphocytes	0.68 ± 1.08	0.42 ± 0.46	2.40E-03
B lineage	0.76 ± 3.16	0.28 ± 0.73	0.05
NK cells	0.09 ± 0.13	0.07 ± 0.08	0.09
Monocytic lineage	4.37 ± 3.48	2.56 ± 1.35	2.80E-10
Myeloid dendritic cells	1.15 ± 1.22	0.77 ± 0.65	1.80E-04
Neutrophils	4.39 ± 2.56	5.68 ± 2.82	5.00E-06
Endothelial cells	2.67 ± 1.17	2.76 ± 1.48	0.48
Fibroblasts	28.45 ± 48.11	18.16 ± 24.46	9.90E-03

**Table 4:** The expression levels of immune cells were abnormal in the high- and low-risk groups.

Immune cell	High-risk group	Low-risk group	*t*-test
B cells naïve	0.03 ± 0.04	0.04 ± 0.04	0.02
B cells memory	1.2e-3 ± 6.4e-3	6.7e-5 ± 5.0e-4	0.01
Plasma cells	0.02 ± 0.04	0.02 ± 0.04	0.23
T cells CD8	0.10 ± 0.10	0.10 ± 0.09	0.77
T cells CD4 naïve	1.1e-3 ± 7.8e-3	2.0e-4 ± 2.7e-3	0.12
T cells CD4 memory resting	0.15 ± 0.11	0.19 ± 0.11	2.20E-04
T cells CD4 memory activated	8.7e-3 ± 0.02	2.6e-3 ± 0.01	7.80E-04
T cells follicular helper	0.02 ± 0.02	0.01 ± 0.02	0.01
T cells regulatory (Tregs)	0.05 ± 0.04	0.05 ± 0.04	0.11
T cells gamma delta	0.01 ± 0.02	0.02 ± 0.03	0.09
NK cells resting	4.5e-3 ± 0.02	9.2e-3 ± 0.02	0.03
NK cells activated	0.06 ± 0.03	0.06 ± 0.03	0.7
Monocytes	0.02 ± 0.03	0.03 ± 0.03	0.04
Macrophages M0	0.16 ± 0.13	0.09 ± 0.08	2.10E-09
Macrophages M1	0.08 ± 0.04	0.09 ± 0.04	4.80E-03
Macrophages M2	0.18 ± 0.09	0.19 ± 0.09	0.06
Dendritic cells resting	0.04 ± 0.05	0.03 ± 0.03	0.02
Dendritic cells activated	2.0e-3 ± 0.01	4.7e-4 ± 3.8e-3	0.1
Mast cells resting	0.06 ± 0.05	0.07 ± 0.06	4.30E-03
Mast cells activated	1.2e-3 ± 7.6e-3	2.0e-3 ± 0.02	0.53
Eosinophils	9.4e-4 ± 4.2e-3	1.9e-4 ± 1.1e-3	0.02
Neutrophils	7.3e-3 ± 0.02	3.4e-3 ± 8.0e-3	0.02

## Data Availability

The genomic data used to support the findings of this study have been deposited in the TCGA and GEO database. These data can also be obtained from the corresponding author.

## Supplementary Materials

Supplementary Materials Figure S1. Hub sorafenib-response target genes in the PPI network. Abbreviation: PPI, protein-protein interaction. Figure S2. The expression levels and prognostic values of hub sorafenib-response genes in HCC based on patient information deposited in the GEPIA database. Abbreviations: GEPIA, Gene Expression Profiling Interactive Analysis; HCC, hepatocellular carcinoma. Figure S3. The prognostic values of sorafenib-response target genes in the TCGA database were determined by performing K-M survival analysis. Abbreviations: K-M, Kaplan–Meier; TCGA, The Cancer Genome Atlas. Figure S4. The diagnostic values of sorafenib-response target genes in the TCGA database were determined by performing ROC analysis. Abbreviations: ROC, receiver operating characteristic; TCGA, The Cancer Genome Atlas. Figure S5. Univariate Cox regression analysis showed that the overexpression of sorafenib-response target genes affected the dismal prognosis of patients with HCC. Abbreviation: HCC, hepatocellular carcinoma. Figure S6. Cox regression analysis showed that risk score was an independent risk factor for a poor prognosis for patients with HCC. Abbreviation: HCC, hepatocellular carcinoma. Figure S7. Sorafenib-response target genes associated with the high- and low-risk groups. Table S1. Differentially expressed genes in the tissues of patients with HCC who responded to treatment with sorafenib. Abbreviation: HCC, hepatocellular carcinoma. Table S2. Functions of sorafenib-response targets as determined by performing GO analysis. Abbreviation: GO, Gene Ontology. Table S3. Differentially expressed genes in the tissues of patients with HCC. Abbreviation: HCC, hepatocellular carcinoma. Table S4. The UHSP90AA1-expression level correlated with the clinicopathological features of patients with HCC. Abbreviations: AHTI, adjacent hepatic tissue inflammation; FIS, fibrosis Ishak score; HCC, hepatocellular carcinoma. Table S5. The LRP4-expression level correlated with the clinicopathological features of patients with HCC. Abbreviations: AHTI, adjacent hepatic tissue inflammation; FIS, fibrosis Ishak score; HCC, hepatocellular carcinoma. Table S6. The PPM1G-expression level correlated with the clinicopathological features of patients with HCC. Abbreviations: AHTI, adjacent hepatic tissue inflammation; FIS, fibrosis Ishak score; HCC, hepatocellular carcinoma. Table S7. The SEC61A1-expression level correlated with the clinicopathological features of patients with HCC. Abbreviations: AHTI, adjacent hepatic tissue inflammation; FIS, fibrosis Ishak score; HCC, hepatocellular carcinoma. Table S8. The SLC41A3-expression level correlated with the clinicopathological features of patients with HCC. Abbreviations: AHTI, adjacent hepatic tissue inflammation; FIS, fibrosis Ishak score; HCC, hepatocellular carcinoma.
