# Supplementary material for: A Risk Model Based on Sorafenib-Response Target Genes Predicts the Prognosis of Patients with HCC
Source: J Oncol. 2022 Jun 28;2022:7257738. doi: 10.1155/2022/7257738 (PMC9256406; doi:10.1155/2022/7257738)
Supplement: Supplementary Materials — Figure S1. Hub sorafenib-response target genes in the PPI network. Abbreviation: PPI, protein-protein interaction. Figure S2. The expression levels and prognostic values of hub sorafenib-response genes in HCC based on patient information deposited in the GEPIA database. Abbreviations: GEPIA, Gene Expression Profiling Interactive Analysis; HCC, hepatocellular carcinoma. Figure S3. The prognostic values of sorafenib-response target genes in the TCGA database were determined by performing K-M survival analysis. Abbreviations: K-M, Kaplan–Meier; TCGA, The Cancer Genome Atlas. Figure S4. The diagnostic values of sorafenib-response target genes in the TCGA database were determined by performing ROC analysis. Abbreviations: ROC, receiver operating characteristic; TCGA, The Cancer Genome Atlas. Figure S5. Univariate Cox regression analysis showed that the overexpression of sorafenib-response target genes affected the dismal prognosis of patients with HCC. Abbreviation: HCC, hepatocellular carcinoma. Figure S6. Cox regression analysis showed that risk score was an independent risk factor for a poor prognosis for patients with HCC. Abbreviation: HCC, hepatocellular carcinoma. Figure S7. Sorafenib-response target genes associated with the high- and low-risk groups. Table S1. Differentially expressed genes in the tissues of patients with HCC who responded to treatment with sorafenib. Abbreviation: HCC, hepatocellular carcinoma. Table S2. Functions of sorafenib-response targets as determined by performing GO analysis. Abbreviation: GO, Gene Ontology. Table S3. Differentially expressed genes in the tissues of patients with HCC. Abbreviation: HCC, hepatocellular carcinoma. Table S4. The UHSP90AA1-expression level correlated with the clinicopathological features of patients with HCC. Abbreviations: AHTI, adjacent hepatic tissue inflammation; FIS, fibrosis Ishak score; HCC, hepatocellular carcinoma. Table S5. The LRP4-expression level correlated with the clinicopathological features [file 7257738.f1.docx]

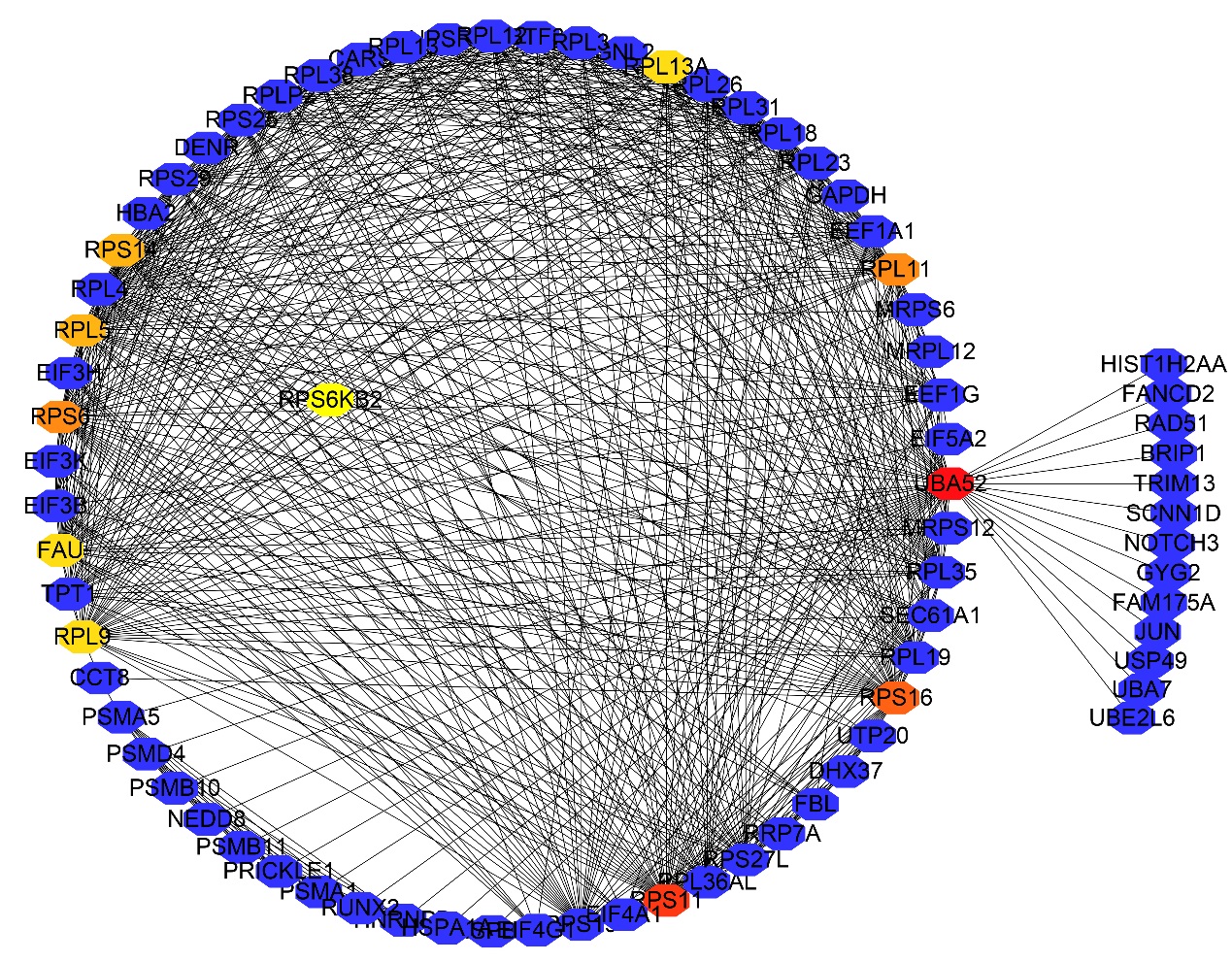
Figure S1. Hub sorafenib-response target genes in the PPI network. Abbreviation: PPI, protein-protein interaction.


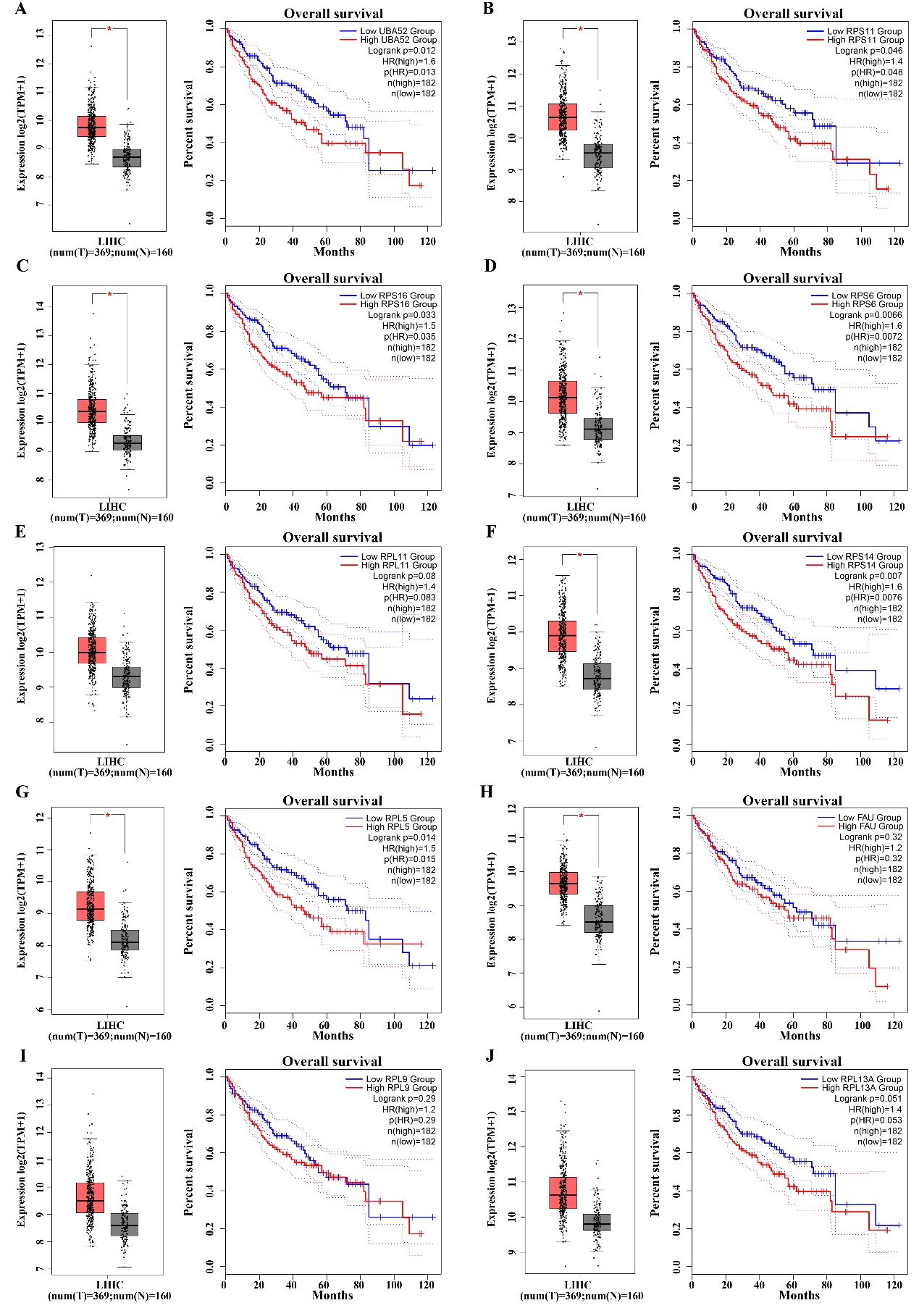
 Figure S2. The expression levels and prognostic values of hub sorafenib-response genes in HCC, based on patient information deposited in the GEPIA database. Abbreviations: GEPIA, Gene Expression Profiling Interactive Analysis; HCC, hepatocellular carcinoma


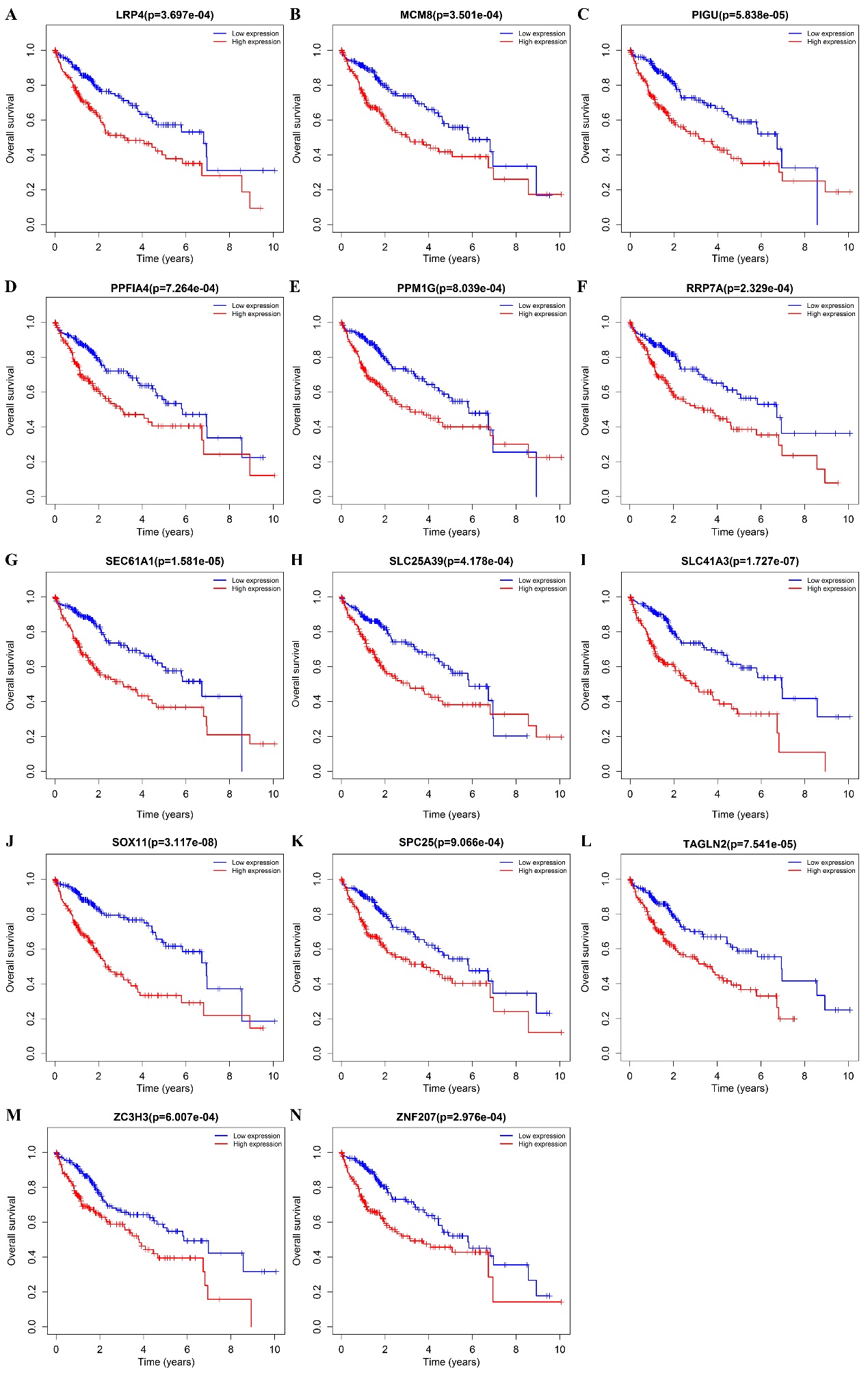
 Figure S3. The prognostic values of sorafenib-response target genes in the TCGA database were determined by performing K-M survival analysis. Abbreviations: K-M, Kaplan-Meier; TCGA, The Cancer Genome Atlas


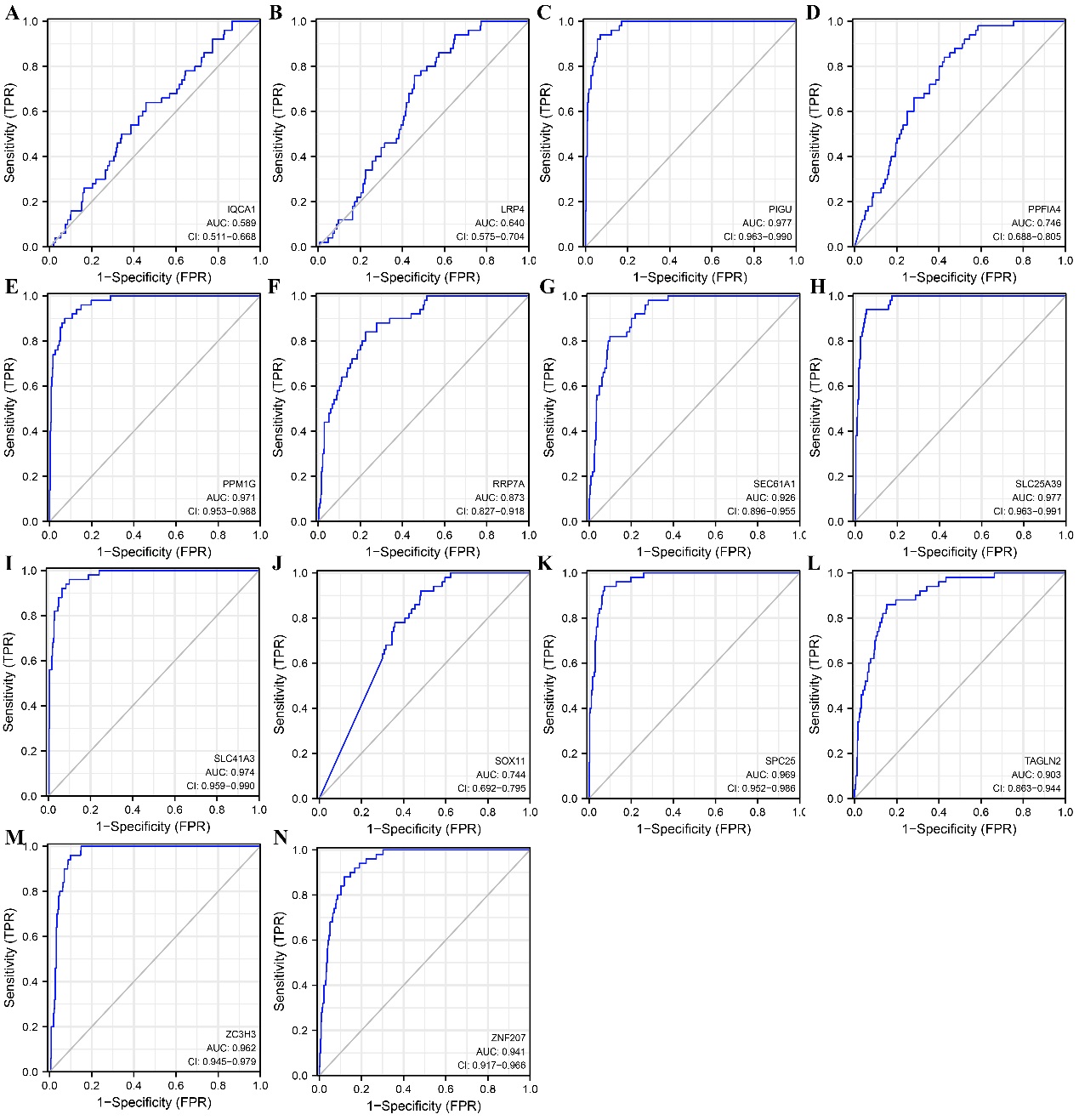
 Figure S4. The diagnostic values of sorafenib-response target genes in the TCGA database were determined by performing ROC analysis. Abbreviations: ROC, receiver operating characteristic; TCGA, The Cancer Genome Atlas


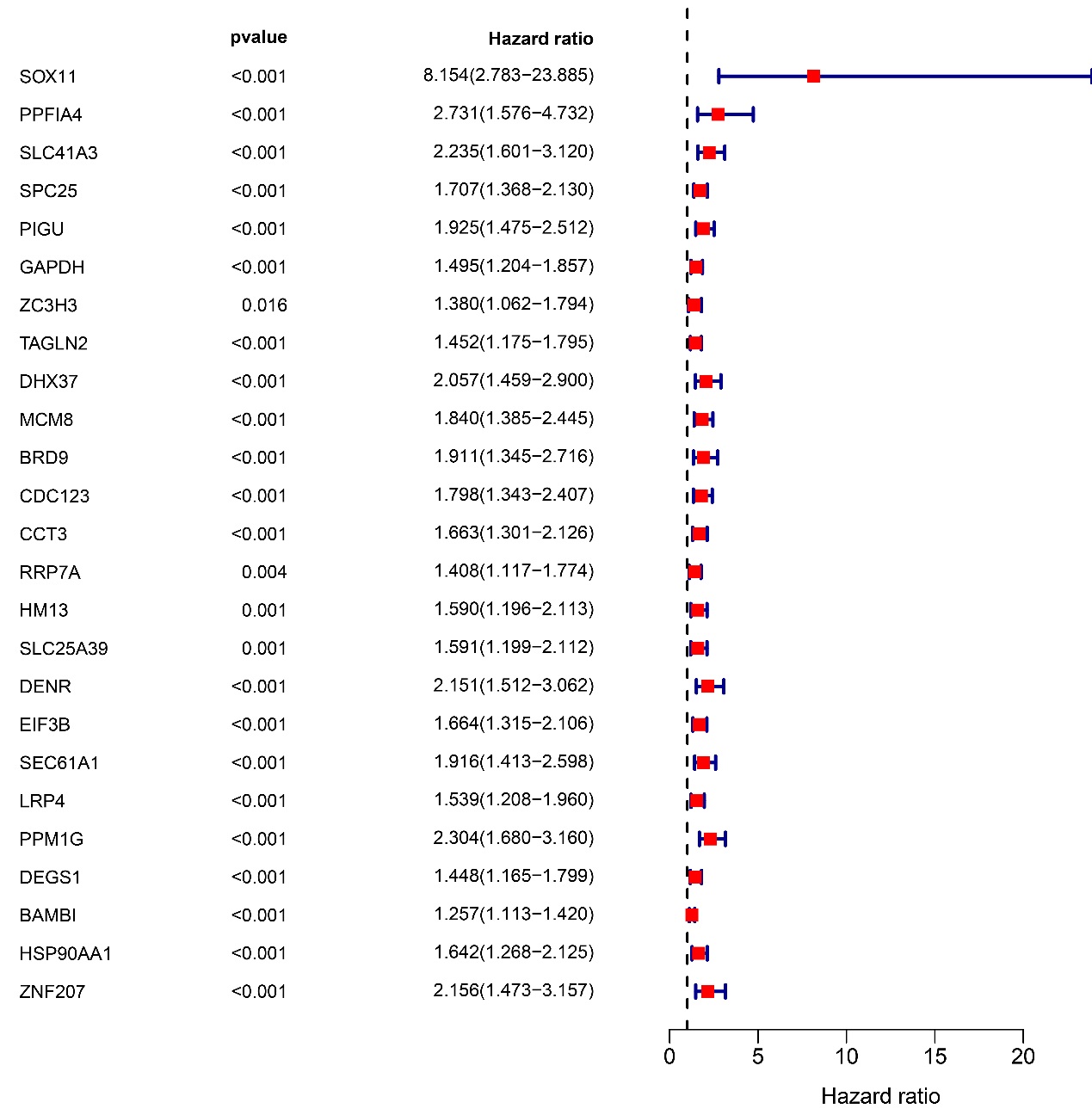
 Figure S5. Univariate Cox regression analysis showed that the overexpression of sorafenib-response target genes affected the dismal prognosis of patients with HCC. Abbreviation: HCC, hepatocellular carcinoma


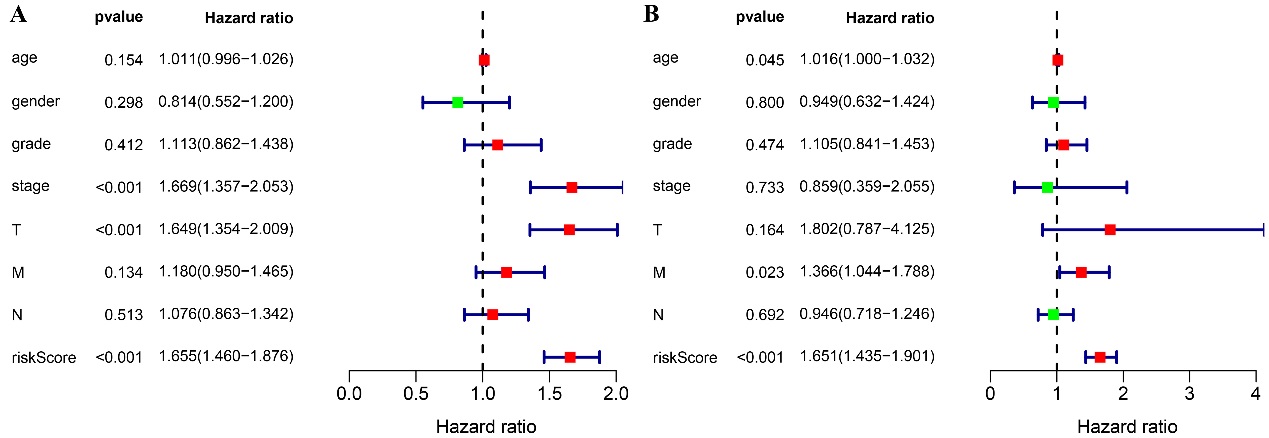
 Figure S6. Cox regression analysis showed that risk score was an independent risk factor for a poor prognosis for patients with HCC. Abbreviation: HCC, hepatocellular carcinoma


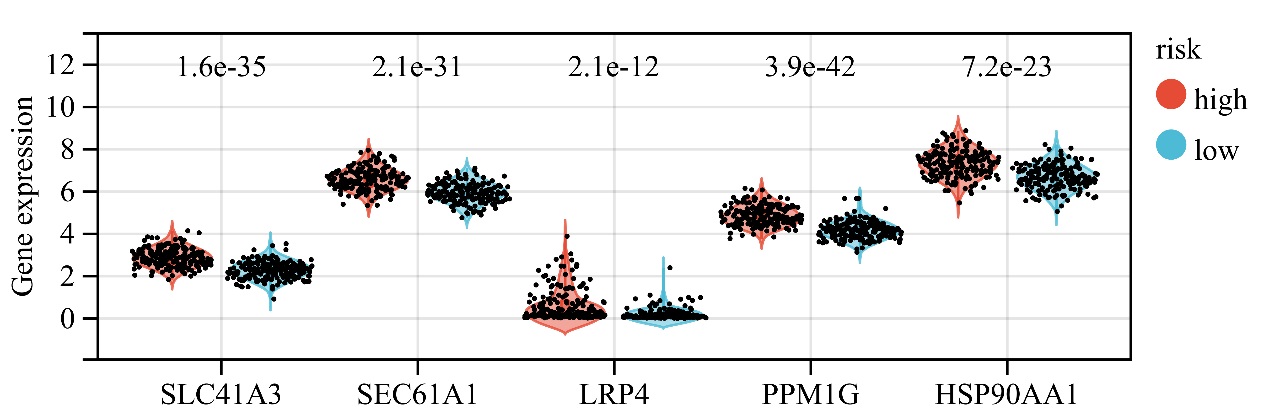
 Figure S7. Sorafenib-response target genes associated with the high- and low-risk groups

Table S1. Differentially expressed genes in the tissues of patients with HCC who responded to treatment with sorafenib.

| id | Gene | logFC | P value | adj P value |
| --- | --- | --- | --- | --- |
| ILMN_2359168 | A2BP1 | -2.8615567 | 9.35E-11 | 1.57E-09 |
| ILMN_1814316 | A2BP1 | -2.7447497 | 1.15E-11 | 2.83E-10 |
| ILMN_1745607 | A2M | 2.3224366 | 7.64E-11 | 1.33E-09 |
| ILMN_1767388 | AAA1 | -1.9559234 | 4.43E-09 | 4.29E-08 |
| ILMN_1662364 | AARS | 1.7327757 | 4.93E-09 | 4.70E-08 |
| ILMN_1802404 | ABCC1 | -1.5219609 | 2.84E-09 | 2.91E-08 |
| ILMN_1767129 | ABCC8 | -2.3220481 | 1.48E-09 | 1.64E-08 |
| ILMN_1658176 | ABCG1 | -1.8766352 | 3.58E-16 | 7.95E-14 |
| ILMN_1699750 | ABHD9 | -1.8947549 | 1.35E-09 | 1.51E-08 |
| ILMN_1758825 | ABLIM2 | -2.0832785 | 2.47E-10 | 3.56E-09 |
| ILMN_1738921 | ACAA1 | 1.7212054 | 8.12E-09 | 7.17E-08 |
| ILMN_1763852 | ACACB | 2.4397065 | 1.47E-11 | 3.44E-10 |
| ILMN_1806408 | ACADVL | 2.0892838 | 2.60E-14 | 2.11E-12 |
| ILMN_2106002 | ACBD7 | -2.4683168 | 1.99E-20 | 1.05E-16 |
| ILMN_1780630 | ACCSL | -2.2510583 | 2.06E-10 | 3.05E-09 |
| ILMN_2371379 | ACLY | 1.748244 | 4.19E-07 | 2.24E-06 |
| ILMN_1654861 | ACO2 | 1.6104331 | 2.49E-15 | 3.36E-13 |
| ILMN_1685703 | ACOX2 | 1.6534357 | 4.79E-10 | 6.23E-09 |
| ILMN_1728331 | ACPT | -2.1611374 | 8.25E-08 | 5.37E-07 |
| ILMN_1705247 | ACSL5 | 1.7942284 | 2.08E-08 | 1.62E-07 |
| ILMN_1746128 | ACSM2B | 1.5032681 | 1.71E-06 | 7.81E-06 |
| ILMN_2336595 | ACSS2 | 1.8417857 | 1.45E-10 | 2.28E-09 |
| ILMN_1714197 | ACSS2 | 2.3696079 | 3.67E-12 | 1.09E-10 |
| ILMN_1671703 | ACTA2 | 2.1471597 | 1.08E-10 | 1.77E-09 |
| ILMN_2038777 | ACTB | 1.6891863 | 3.36E-11 | 6.71E-10 |
| ILMN_2152131 | ACTB | 1.7540702 | 1.81E-07 | 1.07E-06 |
| ILMN_1657855 | ACTL6B | -2.3702173 | 2.50E-11 | 5.32E-10 |
| ILMN_1792314 | ACTR1A | 1.7626073 | 1.08E-13 | 6.39E-12 |
| ILMN_1683883 | ACY1 | 2.137452 | 6.50E-12 | 1.76E-10 |
| ILMN_1756920 | ADAM15 | 2.3830007 | 3.38E-14 | 2.60E-12 |
| ILMN_1726247 | ADAMTS16 | -1.6989753 | 2.91E-08 | 2.16E-07 |
| ILMN_1721495 | ADAMTSL2 | 1.8356326 | 7.64E-07 | 3.81E-06 |
| ILMN_1759252 | ADD1 | 2.1856266 | 1.93E-11 | 4.29E-10 |
| ILMN_1764309 | ADH1A | 1.8476061 | 2.35E-09 | 2.46E-08 |
| ILMN_1811598 | ADH1B | 1.7335849 | 1.25E-08 | 1.04E-07 |
| ILMN_1740717 | ADH1C | 1.6814149 | 2.54E-08 | 1.93E-07 |
| ILMN_1775045 | ADIPOQ | -2.1340191 | 6.04E-14 | 4.08E-12 |
| ILMN_3307926 | ADRBK1 | 1.9746445 | 7.58E-17 | 2.58E-14 |
| ILMN_1813530 | AGT | 2.116866 | 1.42E-10 | 2.24E-09 |
| ILMN_1709796 | AGXT | 2.598061 | 1.93E-11 | 4.29E-10 |
| ILMN_1730625 | AHSG | 2.031468 | 1.61E-08 | 1.29E-07 |
| ILMN_2261519 | AIRE | -2.00669 | 6.89E-13 | 2.76E-11 |
| ILMN_1728047 | AKR1A1 | 1.9657632 | 1.24E-11 | 3.00E-10 |
| ILMN_2412336 | AKR1C2 | 2.0653505 | 2.80E-11 | 5.80E-10 |
| ILMN_1713124 | AKR1C3 | 2.5405082 | 3.77E-09 | 3.73E-08 |
| ILMN_2388507 | AKT1 | 2.1597999 | 7.81E-09 | 6.94E-08 |
| ILMN_1782939 | ALB | 1.8358249 | 2.23E-06 | 9.91E-06 |
| ILMN_1682763 | ALB | 2.9954439 | 1.90E-10 | 2.85E-09 |
| ILMN_1709348 | ALDH1A1 | 2.5796355 | 2.84E-14 | 2.27E-12 |
| ILMN_2096372 | ALDH1A1 | 2.6604916 | 6.86E-13 | 2.76E-11 |
| ILMN_1793859 | ALDH2 | 2.1963934 | 4.55E-16 | 9.38E-14 |
| ILMN_1696099 | ALDH4A1 | 1.5172364 | 4.20E-07 | 2.24E-06 |
| ILMN_1656368 | ALDH4A1 | 2.8571185 | 1.39E-14 | 1.30E-12 |
| ILMN_2372403 | ALDH5A1 | 1.5403023 | 1.61E-11 | 3.70E-10 |
| ILMN_1761804 | ALDH9A1 | 1.7721377 | 5.77E-08 | 3.92E-07 |
| ILMN_1741148 | ALDOA | 1.5320171 | 2.76E-11 | 5.74E-10 |
| ILMN_1747716 | ALDOB | 2.2899605 | 1.66E-10 | 2.53E-09 |
| ILMN_2271014 | ALG8 | -1.7976356 | 9.11E-15 | 9.40E-13 |
| ILMN_1692332 | ALOX12B | -3.5478621 | 1.78E-14 | 1.59E-12 |
| ILMN_1693789 | ALPP | -1.7023741 | 2.31E-16 | 5.69E-14 |
| ILMN_2397406 | AMELX | -1.956319 | 7.60E-11 | 1.32E-09 |
| ILMN_1760727 | ANG | 1.6848646 | 2.03E-08 | 1.59E-07 |
| ILMN_1758443 | ANGPT4 | -1.9076839 | 1.05E-09 | 1.22E-08 |
| ILMN_1695193 | ANGPTL3 | 2.2582097 | 7.94E-09 | 7.03E-08 |
| ILMN_1707727 | ANGPTL4 | 2.3221271 | 6.74E-11 | 1.20E-09 |
| ILMN_1716264 | ANKRD1 | -1.5673906 | 1.58E-06 | 7.25E-06 |
| ILMN_1737612 | ANXA13 | -1.6621561 | 2.25E-12 | 7.30E-11 |
| ILMN_3242004 | ANXA8L1 | -2.0310604 | 7.87E-11 | 1.36E-09 |
| ILMN_1754738 | AP1M1 | 1.9799548 | 5.88E-13 | 2.45E-11 |
| ILMN_1809957 | AP2S1 | 1.6205427 | 3.27E-08 | 2.40E-07 |
| ILMN_1701774 | AP3M1 | -2.4683727 | 9.13E-17 | 2.87E-14 |
| ILMN_1659950 | APC2 | -2.4675647 | 1.45E-08 | 1.18E-07 |
| ILMN_1690884 | APOA1 | 2.819661 | 1.26E-09 | 1.43E-08 |
| ILMN_1688543 | APOA2 | 2.1439297 | 3.78E-08 | 2.73E-07 |
| ILMN_1664024 | APOB | 2.684826 | 7.75E-11 | 1.34E-09 |
| ILMN_1664828 | APOBEC3H | -1.5881029 | 3.50E-12 | 1.06E-10 |
| ILMN_1789007 | APOC1 | 2.1643967 | 2.57E-10 | 3.68E-09 |
| ILMN_1802923 | APOC2 | 2.4368214 | 1.24E-11 | 3.00E-10 |
| ILMN_1722070 | APOC3 | 2.3424273 | 6.18E-10 | 7.76E-09 |
| ILMN_1740938 | APOE | 2.0501576 | 2.90E-07 | 1.62E-06 |
| ILMN_1731941 | APOM | 1.5388503 | 1.28E-09 | 1.45E-08 |
| ILMN_1726410 | APRT | 1.6133108 | 6.79E-08 | 4.53E-07 |
| ILMN_2090004 | AQP10 | -1.5698725 | 1.32E-07 | 8.13E-07 |
| ILMN_1711120 | ARC | -1.5909651 | 3.81E-10 | 5.09E-09 |
| ILMN_1721977 | ARD1A | 1.6292479 | 3.19E-11 | 6.44E-10 |
| ILMN_1802203 | ARF1 | 2.0474779 | 8.82E-15 | 9.16E-13 |
| ILMN_1723884 | ARF4 | 1.873905 | 5.77E-15 | 6.45E-13 |
| ILMN_2297096 | ARHGAP8 | -2.5159556 | 1.12E-19 | 2.73E-16 |
| ILMN_1678143 | ARHGDIB | 1.5360116 | 1.01E-07 | 6.41E-07 |
| ILMN_1698680 | ARL17P1 | -1.9390665 | 1.42E-11 | 3.35E-10 |
| ILMN_1787879 | ARL2 | 1.6276633 | 2.04E-14 | 1.77E-12 |
| ILMN_2183510 | ARMET | 2.0269257 | 3.08E-12 | 9.48E-11 |
| ILMN_1656477 | ARSA | 2.0362543 | 2.16E-10 | 3.18E-09 |
| ILMN_1679919 | ASCC2 | 1.6623305 | 5.84E-13 | 2.44E-11 |
| ILMN_1694966 | ASGR2 | 1.5263976 | 3.79E-08 | 2.73E-07 |
| ILMN_2342638 | ASGR2 | 1.5391373 | 4.43E-10 | 5.81E-09 |
| ILMN_1660749 | ASPSCR1 | 2.9987214 | 1.30E-17 | 6.59E-15 |
| ILMN_1708778 | ASS1 | 1.5202312 | 2.64E-08 | 1.99E-07 |
| ILMN_1672128 | ATF4 | 2.5982772 | 1.86E-11 | 4.16E-10 |
| ILMN_1734655 | ATG9B | -1.5546449 | 5.82E-11 | 1.06E-09 |
| ILMN_1670609 | ATOX1 | 1.8656594 | 2.02E-09 | 2.16E-08 |
| ILMN_2134224 | ATP13A1 | 1.9004316 | 4.95E-15 | 5.76E-13 |
| ILMN_1731783 | ATP1A1 | 1.9886363 | 2.51E-13 | 1.25E-11 |
| ILMN_1674460 | ATP2A1 | -1.8736134 | 3.06E-08 | 2.26E-07 |
| ILMN_1772132 | ATP5B | 1.5308587 | 2.68E-12 | 8.41E-11 |
| ILMN_1676393 | ATP5G1 | 1.9426649 | 2.76E-10 | 3.90E-09 |
| ILMN_1666372 | ATP5H | 2.2783615 | 2.38E-14 | 1.98E-12 |
| ILMN_1726603 | ATP5I | 2.2932489 | 1.42E-11 | 3.35E-10 |
| ILMN_2348093 | ATP5J | 1.5219165 | 2.05E-09 | 2.19E-08 |
| ILMN_1772929 | ATP5J | 1.641942 | 1.89E-10 | 2.83E-09 |
| ILMN_2307883 | ATP5J2 | 1.8541464 | 7.92E-10 | 9.59E-09 |
| ILMN_2310621 | ATP5J2 | 1.9454973 | 9.45E-10 | 1.12E-08 |
| ILMN_1697694 | ATP6AP1 | 1.9208542 | 3.79E-13 | 1.75E-11 |
| ILMN_1752579 | ATP6V0A1 | 1.9856201 | 4.69E-15 | 5.54E-13 |
| ILMN_2394287 | ATP6V0A4 | -1.530276 | 2.00E-09 | 2.14E-08 |
| ILMN_1785095 | ATP6V0E2 | 2.0659621 | 1.38E-13 | 7.65E-12 |
| ILMN_1812073 | ATP6V1B1 | -1.570357 | 6.07E-08 | 4.11E-07 |
| ILMN_1693896 | ATP8A2 | -1.6492395 | 1.38E-09 | 1.54E-08 |
| ILMN_2089073 | ATP9A | 1.6457139 | 3.81E-08 | 2.74E-07 |
| ILMN_2231911 | AUH | 1.7207995 | 4.64E-11 | 8.80E-10 |
| ILMN_1768396 | AURKAIP1 | 2.3767318 | 1.79E-14 | 1.59E-12 |
| ILMN_1721333 | AURKC | -1.6101379 | 1.37E-06 | 6.40E-06 |
| ILMN_1811443 | AVP | -1.5937636 | 7.88E-08 | 5.15E-07 |
| ILMN_1703123 | AXUD1 | 1.7556731 | 3.68E-12 | 1.09E-10 |
| ILMN_1725427 | B2M | 1.6305587 | 6.17E-12 | 1.68E-10 |
| ILMN_1771308 | BAAT | 1.5498074 | 1.56E-10 | 2.41E-09 |
| ILMN_1699727 | BAIAP2 | 1.500259 | 1.39E-06 | 6.45E-06 |
| ILMN_1771652 | BAIAP2L2 | 1.5285735 | 1.06E-06 | 5.07E-06 |
| ILMN_1691410 | BAMBI | 1.7801383 | 1.73E-09 | 1.88E-08 |
| ILMN_2248912 | BCAS4 | -1.9455065 | 1.08E-15 | 1.77E-13 |
| ILMN_1735979 | BCKDHA | 1.8197855 | 1.60E-12 | 5.54E-11 |
| ILMN_1693394 | BCKDK | 2.1624617 | 1.87E-13 | 9.82E-12 |
| ILMN_1719820 | BDNF | -1.8316558 | 1.41E-12 | 5.07E-11 |
| ILMN_1809543 | BDNF | -1.5465241 | 5.11E-11 | 9.58E-10 |
| ILMN_2375032 | BEND3 | -1.6870789 | 3.01E-11 | 6.15E-10 |
| ILMN_3230853 | BEND7 | -2.05812 | 1.55E-11 | 3.58E-10 |
| ILMN_1755796 | BEST2 | -1.8318001 | 1.22E-09 | 1.39E-08 |
| ILMN_2206746 | BGN | 2.7167644 | 2.10E-11 | 4.62E-10 |
| ILMN_3238183 | BMS1P5 | -1.727177 | 9.03E-11 | 1.52E-09 |
| ILMN_1708513 | BNIPL | -1.6179228 | 4.55E-10 | 5.95E-09 |
| ILMN_1758918 | BRD2 | 1.912284 | 2.94E-13 | 1.41E-11 |
| ILMN_1651405 | BRD9 | 2.0326372 | 1.67E-16 | 4.46E-14 |
| ILMN_1750821 | BRDT | -1.7446607 | 6.40E-06 | 2.54E-05 |
| ILMN_1760757 | BRIP1 | -1.6567193 | 3.76E-14 | 2.84E-12 |
| ILMN_1679800 | BRIX1 | 1.6298966 | 4.20E-11 | 8.10E-10 |
| ILMN_2398432 | BRMS1 | 2.3153557 | 5.80E-15 | 6.45E-13 |
| ILMN_1659762 | BTF3 | 1.6688171 | 2.44E-08 | 1.86E-07 |
| ILMN_1663749 | BTNL3 | -2.0361616 | 8.24E-10 | 9.92E-09 |
| ILMN_1770130 | C10orf113 | -1.8665461 | 2.37E-08 | 1.82E-07 |
| ILMN_1680110 | C10orf116 | 2.1422301 | 6.55E-10 | 8.18E-09 |
| ILMN_1674373 | C10orf27 | -1.9055997 | 5.06E-12 | 1.42E-10 |
| ILMN_1681967 | C10orf4 | -2.0611376 | 2.56E-12 | 8.14E-11 |
| ILMN_1802192 | C10orf99 | -2.0956584 | 5.47E-14 | 3.77E-12 |
| ILMN_2077094 | C11orf2 | 2.5812445 | 7.24E-16 | 1.34E-13 |
| ILMN_1680102 | C11orf45 | -2.0178656 | 2.66E-10 | 3.79E-09 |
| ILMN_1664773 | C11orf49 | 1.6358395 | 1.62E-10 | 2.48E-09 |
| ILMN_1715567 | C12orf50 | -1.726975 | 7.03E-12 | 1.88E-10 |
| ILMN_1706186 | C12orf53 | -2.0735834 | 1.07E-08 | 9.11E-08 |
| ILMN_1754410 | C12orf72 | -1.8775362 | 4.59E-13 | 2.02E-11 |
| ILMN_1740165 | C14orf102 | 1.5405072 | 6.96E-09 | 6.29E-08 |
| ILMN_1777708 | C14orf121 | -2.5238319 | 9.45E-12 | 2.41E-10 |
| ILMN_2078724 | C14orf153 | -2.3117901 | 2.25E-14 | 1.93E-12 |
| ILMN_2393450 | C14orf173 | 2.511058 | 1.18E-12 | 4.37E-11 |
| ILMN_2134381 | C14orf85 | -1.7437552 | 1.79E-11 | 4.03E-10 |
| ILMN_1773848 | C15orf32 | -1.5810184 | 7.55E-14 | 4.86E-12 |
| ILMN_2145490 | C15orf60 | -1.6716742 | 7.70E-08 | 5.05E-07 |
| ILMN_1806189 | C16orf3 | -1.7231651 | 3.09E-09 | 3.13E-08 |
| ILMN_1787324 | C16orf48 | 1.7609801 | 1.14E-08 | 9.61E-08 |
| ILMN_1685289 | C16orf58 | 2.0426112 | 1.42E-12 | 5.12E-11 |
| ILMN_1765645 | C17orf50 | -2.3558147 | 2.77E-10 | 3.90E-09 |
| ILMN_1711823 | C17orf70 | 2.1851961 | 9.48E-15 | 9.63E-13 |
| ILMN_1664920 | C19orf12 | 1.6460482 | 8.54E-08 | 5.53E-07 |
| ILMN_1684854 | C19orf41 | -2.0689654 | 3.87E-13 | 1.76E-11 |
| ILMN_1759184 | C19orf48 | 1.9650204 | 2.40E-13 | 1.21E-11 |
| ILMN_1671374 | C19orf53 | 2.529397 | 8.54E-13 | 3.29E-11 |
| ILMN_1762439 | C19orf6 | 1.7176743 | 5.86E-10 | 7.39E-09 |
| ILMN_1750400 | C19orf66 | 2.3572237 | 8.60E-16 | 1.53E-13 |
| ILMN_1656088 | C1orf110 | -3.528594 | 4.13E-16 | 8.76E-14 |
| ILMN_1762204 | C1orf150 | -1.8679111 | 3.49E-16 | 7.81E-14 |
| ILMN_2387496 | C1orf152 | -1.7388687 | 1.49E-15 | 2.24E-13 |
| ILMN_1675024 | C1orf165 | -1.7683893 | 3.37E-10 | 4.61E-09 |
| ILMN_2250853 | C1orf84 | -2.0475743 | 1.84E-15 | 2.70E-13 |
| ILMN_1756236 | C1QL2 | -1.5930276 | 1.10E-06 | 5.26E-06 |
| ILMN_1808117 | C1QL4 | -2.0541825 | 3.74E-07 | 2.02E-06 |
| ILMN_1677198 | C1R | 1.7027004 | 6.73E-12 | 1.81E-10 |
| ILMN_1764109 | C1R | 2.1160075 | 1.79E-10 | 2.70E-09 |
| ILMN_1781626 | C1S | 2.4580336 | 5.18E-16 | 1.02E-13 |
| ILMN_1710740 | C2 | 1.5380468 | 2.95E-10 | 4.11E-09 |
| ILMN_1790136 | C20orf20 | 2.2427162 | 1.08E-12 | 4.07E-11 |
| ILMN_3249045 | C21orf54 | -2.2036433 | 3.72E-13 | 1.73E-11 |
| ILMN_3307877 | C21orf58 | -1.7927768 | 9.61E-18 | 5.12E-15 |
| ILMN_3240717 | C2orf14 | -1.9738453 | 1.56E-17 | 7.39E-15 |
| ILMN_1708906 | C2orf29 | 1.6188071 | 4.23E-11 | 8.14E-10 |
| ILMN_1655269 | C2orf66 | -2.559094 | 9.25E-10 | 1.09E-08 |
| ILMN_3236377 | C2orf69 | -2.0397836 | 9.37E-19 | 9.42E-16 |
| ILMN_2280542 | C2orf78 | -2.0127075 | 2.65E-16 | 6.35E-14 |
| ILMN_1662523 | C3 | 1.6126159 | 5.39E-08 | 3.70E-07 |
| ILMN_1762260 | C3 | 2.4796324 | 1.05E-13 | 6.24E-12 |
| ILMN_3242335 | C3orf51 | -2.2009894 | 7.74E-13 | 3.03E-11 |
| ILMN_1810752 | C4BPA | 1.9013457 | 5.71E-11 | 1.05E-09 |
| ILMN_1746819 | C5 | 1.5649717 | 4.34E-13 | 1.94E-11 |
| ILMN_1662184 | C5orf34 | -1.6616093 | 1.04E-07 | 6.56E-07 |
| ILMN_3240287 | C5orf40 | -1.7825866 | 7.88E-13 | 3.08E-11 |
| ILMN_1790461 | C6orf125 | 1.6100966 | 3.69E-12 | 1.10E-10 |
| ILMN_2307407 | C6orf25 | -2.1829443 | 8.39E-12 | 2.19E-10 |
| ILMN_1712836 | C6orf27 | -1.9352089 | 1.08E-09 | 1.25E-08 |
| ILMN_1739798 | C7orf30 | 1.7347189 | 1.21E-08 | 1.01E-07 |
| ILMN_1718336 | C7orf50 | 1.7653433 | 5.37E-11 | 9.98E-10 |
| ILMN_2198859 | C7orf52 | -1.7753467 | 3.83E-07 | 2.07E-06 |
| ILMN_3243749 | C7orf64 | -1.5972014 | 3.68E-14 | 2.80E-12 |
| ILMN_1790751 | C8orf31 | -1.6335119 | 1.76E-10 | 2.67E-09 |
| ILMN_2113738 | C8orf45 | -1.7677682 | 1.67E-16 | 4.46E-14 |
| ILMN_1777740 | C8orf55 | 1.7255096 | 6.03E-09 | 5.57E-08 |
| ILMN_1777477 | C9orf106 | -1.5411407 | 1.62E-08 | 1.30E-07 |
| ILMN_2077160 | C9orf41 | -1.8564977 | 5.72E-12 | 1.58E-10 |
| ILMN_1725139 | CA9 | -1.8464679 | 6.30E-07 | 3.22E-06 |
| ILMN_1748102 | CACNG5 | -2.4535708 | 1.97E-12 | 6.57E-11 |
| ILMN_1779043 | CACNG6 | -1.9356931 | 1.89E-14 | 1.66E-12 |
| ILMN_1705141 | CACYBP | -1.5088554 | 4.08E-10 | 5.41E-09 |
| ILMN_1666385 | CALM3 | 1.5864364 | 1.16E-08 | 9.70E-08 |
| ILMN_3238986 | CALY | -1.5867213 | 1.22E-07 | 7.58E-07 |
| ILMN_2376194 | CAMK2B | -1.5612856 | 3.16E-07 | 1.75E-06 |
| ILMN_1794863 | CAMK2N1 | 1.6229119 | 7.69E-16 | 1.42E-13 |
| ILMN_1714599 | CAMLG | 1.5662105 | 7.31E-13 | 2.89E-11 |
| ILMN_1765274 | CAPN11 | -1.7419103 | 2.35E-08 | 1.80E-07 |
| ILMN_1737089 | CAPN5 | 1.5776931 | 2.66E-07 | 1.51E-06 |
| ILMN_1731073 | CAPN9 | -2.4986574 | 1.51E-09 | 1.67E-08 |
| ILMN_1690923 | CAPN9 | -1.8861289 | 1.63E-10 | 2.49E-09 |
| ILMN_2393254 | CAPNS1 | 1.9968285 | 1.42E-15 | 2.20E-13 |
| ILMN_1782897 | CAPRIN1 | 1.5028916 | 8.83E-10 | 1.05E-08 |
| ILMN_1809477 | CARHSP1 | 2.1884049 | 3.91E-10 | 5.22E-09 |
| ILMN_2367469 | CARS | 1.9865117 | 7.78E-11 | 1.35E-09 |
| ILMN_1804735 | CBS | 2.2531627 | 9.94E-09 | 8.52E-08 |
| ILMN_1682567 | CCDC106 | 1.8226491 | 2.63E-11 | 5.52E-10 |
| ILMN_1658376 | CCDC114 | -2.0346971 | 2.60E-07 | 1.48E-06 |
| ILMN_2214955 | CCDC127 | -1.6741861 | 8.45E-11 | 1.44E-09 |
| ILMN_1758633 | CCDC130 | 2.4064842 | 1.30E-15 | 2.05E-13 |
| ILMN_3245143 | CCDC19 | -1.7400662 | 4.89E-11 | 9.23E-10 |
| ILMN_1701905 | CCDC62 | -1.7909327 | 3.60E-11 | 7.11E-10 |
| ILMN_1747655 | CCDC74B | -1.575982 | 4.24E-06 | 1.76E-05 |
| ILMN_2232166 | CCDC90B | 1.5461501 | 2.92E-16 | 6.73E-14 |
| ILMN_1653766 | CCL24 | -1.6776575 | 1.43E-07 | 8.71E-07 |
| ILMN_1784352 | CCM2 | 1.7506559 | 2.84E-07 | 1.59E-06 |
| ILMN_2260833 | CCNB1IP1 | -2.3226927 | 5.18E-16 | 1.02E-13 |
| ILMN_1688480 | CCND1 | 1.7658804 | 3.91E-09 | 3.85E-08 |
| ILMN_1715131 | CCR7 | -1.5510218 | 6.57E-10 | 8.20E-09 |
| ILMN_2334989 | CCT3 | 1.5674514 | 1.24E-11 | 3.00E-10 |
| ILMN_1717868 | CCT8 | 1.5780862 | 2.49E-08 | 1.89E-07 |
| ILMN_2326713 | CD151 | 2.6021593 | 1.16E-11 | 2.84E-10 |
| ILMN_2379599 | CD163 | 1.666387 | 3.97E-12 | 1.16E-10 |
| ILMN_1723520 | CD1A | -2.1134821 | 1.81E-10 | 2.73E-09 |
| ILMN_1718754 | CD207 | -2.1577743 | 4.03E-08 | 2.88E-07 |
| ILMN_3240165 | CD300LD | -1.9004492 | 1.88E-16 | 4.84E-14 |
| ILMN_1714861 | CD68 | 2.1848158 | 1.13E-13 | 6.56E-12 |
| ILMN_1761464 | CD74 | 1.6392251 | 4.28E-09 | 4.17E-08 |
| ILMN_1736567 | CD74 | 2.1305071 | 4.63E-16 | 9.47E-14 |
| ILMN_1689953 | CD81 | 2.2106219 | 3.17E-12 | 9.70E-11 |
| ILMN_1698367 | CD84 | -1.5472638 | 1.14E-08 | 9.61E-08 |
| ILMN_2354191 | CD8B | -2.012679 | 8.28E-13 | 3.21E-11 |
| ILMN_2388142 | CD99L2 | 1.7901836 | 3.65E-10 | 4.93E-09 |
| ILMN_1678605 | CDC123 | 2.2612652 | 8.95E-11 | 1.51E-09 |
| ILMN_1668369 | CDC37 | 2.4657302 | 2.77E-11 | 5.75E-10 |
| ILMN_1764927 | CDC42EP1 | 1.5779508 | 6.92E-11 | 1.22E-09 |
| ILMN_1688670 | CDCP1 | -1.659039 | 9.62E-14 | 5.85E-12 |
| ILMN_1791270 | CDH10 | -1.9364164 | 1.02E-13 | 6.11E-12 |
| ILMN_1741459 | CDK10 | 2.0109359 | 2.64E-16 | 6.35E-14 |
| ILMN_1767414 | CDK3 | -1.6628763 | 9.47E-11 | 1.59E-09 |
| ILMN_1784602 | CDKN1A | 1.735115 | 7.41E-11 | 1.30E-09 |
| ILMN_2130078 | CDKN2AIPNL | -1.7397603 | 3.47E-15 | 4.38E-13 |
| ILMN_1801476 | CDS1 | -1.5621822 | 1.74E-07 | 1.03E-06 |
| ILMN_3244898 | CEACAM18 | -1.5495136 | 1.65E-13 | 8.89E-12 |
| ILMN_1715715 | CEBPA | 2.0304538 | 6.69E-14 | 4.38E-12 |
| ILMN_1693014 | CEBPB | 1.8793085 | 4.24E-09 | 4.13E-08 |
| ILMN_1782050 | CEBPD | 1.7845875 | 3.06E-12 | 9.43E-11 |
| ILMN_1664028 | CENPB | 1.970664 | 4.33E-14 | 3.20E-12 |
| ILMN_1738482 | CEP27 | -1.8493054 | 2.62E-15 | 3.52E-13 |
| ILMN_2359945 | CES1 | 1.7256613 | 4.04E-09 | 3.96E-08 |
| ILMN_1696675 | CES2 | 1.5989757 | 2.41E-11 | 5.14E-10 |
| ILMN_2150095 | CES4 | 2.0006854 | 3.40E-09 | 3.41E-08 |
| ILMN_1774287 | CFB | 1.9573335 | 2.51E-10 | 3.61E-09 |
| ILMN_1777190 | CFD | 1.714008 | 8.10E-10 | 9.79E-09 |
| ILMN_1657803 | CFH | 1.5535431 | 1.31E-11 | 3.14E-10 |
| ILMN_1810910 | CFH | 1.5887831 | 6.42E-11 | 1.15E-09 |
| ILMN_2412192 | CFH | 2.1456224 | 2.75E-13 | 1.34E-11 |
| ILMN_1705617 | CFL1 | 2.1791303 | 4.37E-16 | 9.21E-14 |
| ILMN_2163790 | CGB5 | -1.5274101 | 3.71E-08 | 2.68E-07 |
| ILMN_1775997 | CHAT | -2.7045761 | 5.41E-16 | 1.06E-13 |
| ILMN_3249477 | CHIT1 | -2.4332883 | 2.75E-10 | 3.89E-09 |
| ILMN_1729112 | CHPT1 | 2.2693593 | 1.53E-12 | 5.42E-11 |
| ILMN_1761574 | CHRM2 | -1.6572292 | 4.48E-18 | 2.76E-15 |
| ILMN_2361768 | CHRNA1 | -1.9494829 | 3.52E-12 | 1.06E-10 |
| ILMN_1736828 | CHST10 | -1.8556663 | 1.44E-09 | 1.60E-08 |
| ILMN_1734707 | CHST13 | 1.5424295 | 1.68E-09 | 1.84E-08 |
| ILMN_1670881 | CHST6 | -1.9617962 | 5.21E-09 | 4.92E-08 |
| ILMN_1684205 | CIB1 | 2.5477757 | 3.55E-13 | 1.66E-11 |
| ILMN_1788184 | CIDEA | -1.8362912 | 2.47E-13 | 1.24E-11 |
| ILMN_1694731 | CLCN7 | 1.9192118 | 6.37E-13 | 2.62E-11 |
| ILMN_1661194 | CLDN14 | -2.4387794 | 9.22E-19 | 9.42E-16 |
| ILMN_1696284 | CLDN18 | -1.7149848 | 1.14E-09 | 1.31E-08 |
| ILMN_1804531 | CLDN6 | -2.2433425 | 1.06E-08 | 9.03E-08 |
| ILMN_1723115 | CLEC4F | -1.6477996 | 3.60E-06 | 1.52E-05 |
| ILMN_2193817 | CLEC4G | -1.8033184 | 3.05E-08 | 2.26E-07 |
| ILMN_1674609 | CLTB | 1.7314433 | 1.87E-13 | 9.82E-12 |
| ILMN_1710124 | CMTM8 | 2.483113 | 8.31E-11 | 1.42E-09 |
| ILMN_1702383 | CNGB1 | -2.238108 | 2.66E-18 | 1.99E-15 |
| ILMN_1810054 | CNN1 | -1.5551033 | 6.38E-06 | 2.53E-05 |
| ILMN_1770290 | CNN2 | 1.5243403 | 1.30E-14 | 1.22E-12 |
| ILMN_1782439 | CNN3 | 1.5228114 | 7.72E-07 | 3.85E-06 |
| ILMN_1657750 | CNNM1 | -2.0642696 | 5.34E-09 | 5.02E-08 |
| ILMN_1807525 | CNTD2 | -1.6141143 | 6.69E-09 | 6.07E-08 |
| ILMN_1753498 | COASY | 2.2470394 | 1.03E-15 | 1.72E-13 |
| ILMN_1761260 | COBLL1 | 1.6496218 | 1.71E-11 | 3.89E-10 |
| ILMN_1701308 | COL1A1 | 1.6860457 | 4.20E-08 | 2.98E-07 |
| ILMN_1653028 | COL4A1 | 1.5414038 | 2.46E-07 | 1.41E-06 |
| ILMN_1728707 | COLQ | -1.6956328 | 6.23E-11 | 1.12E-09 |
| ILMN_1684385 | COPB2 | 1.6209993 | 4.50E-11 | 8.57E-10 |
| ILMN_1652207 | COX4I1 | 2.9035579 | 2.75E-12 | 8.62E-11 |
| ILMN_1663512 | COX5B | 1.58238 | 1.37E-11 | 3.25E-10 |
| ILMN_1701293 | COX7A2 | 1.7138965 | 4.63E-08 | 3.24E-07 |
| ILMN_1813206 | CP | 2.0529173 | 3.40E-11 | 6.77E-10 |
| ILMN_1744806 | CPB2 | 1.5633089 | 1.55E-11 | 3.59E-10 |
| ILMN_2369603 | CPEB1 | -1.634186 | 5.02E-10 | 6.50E-09 |
| ILMN_1689515 | CPLX3 | -1.7142996 | 3.43E-07 | 1.87E-06 |
| ILMN_1654545 | CPSF1 | 1.6868774 | 1.39E-13 | 7.65E-12 |
| ILMN_1779524 | CREB3L3 | 2.1971553 | 1.70E-12 | 5.81E-11 |
| ILMN_1780575 | CRP | 1.5625502 | 5.49E-05 | 1.75E-04 |
| ILMN_1686362 | CRYBA4 | -1.5937318 | 3.97E-14 | 2.96E-12 |
| ILMN_1779304 | CRYGA | -2.2286092 | 5.58E-12 | 1.55E-10 |
| ILMN_1776266 | CRYGB | -1.7430826 | 5.57E-13 | 2.36E-11 |
| ILMN_2376458 | CSF2RA | -1.8400362 | 1.47E-14 | 1.36E-12 |
| ILMN_1697069 | CSF3 | -2.2191423 | 7.19E-11 | 1.26E-09 |
| ILMN_2391071 | CSH2 | -2.266565 | 3.58E-09 | 3.57E-08 |
| ILMN_1792400 | CSN3 | -2.369172 | 3.19E-09 | 3.22E-08 |
| ILMN_2415235 | CSNK1E | 1.9815144 | 4.52E-14 | 3.30E-12 |
| ILMN_1800461 | CSNK2B | 2.3812319 | 3.38E-14 | 2.60E-12 |
| ILMN_2078995 | CSRNP3 | -1.604028 | 2.52E-13 | 1.25E-11 |
| ILMN_1673843 | CST2 | -1.8945093 | 6.80E-13 | 2.74E-11 |
| ILMN_2088560 | CT45A1 | -1.9813952 | 5.24E-13 | 2.25E-11 |
| ILMN_1661804 | CTF1 | -2.0765352 | 1.40E-07 | 8.55E-07 |
| ILMN_1651430 | CTRB2 | -2.6468304 | 7.13E-15 | 7.65E-13 |
| ILMN_1666269 | CTSZ | 1.7728027 | 4.85E-10 | 6.30E-09 |
| ILMN_1728478 | CXCL16 | 1.5752306 | 1.00E-05 | 3.80E-05 |
| ILMN_1691276 | CXXC1 | 2.2299182 | 5.03E-15 | 5.80E-13 |
| ILMN_2312194 | CYB5A | 1.6798711 | 2.25E-11 | 4.87E-10 |
| ILMN_1714167 | CYB5A | 2.3248796 | 4.39E-11 | 8.38E-10 |
| ILMN_1729237 | CYB5R1 | 1.6971865 | 2.27E-13 | 1.16E-11 |
| ILMN_2061419 | CYCSL1 | -2.0151012 | 1.19E-18 | 1.12E-15 |
| ILMN_3247548 | CYMP | -1.7261533 | 2.12E-14 | 1.82E-12 |
| ILMN_1683607 | CYP1A2 | 1.5112882 | 2.14E-05 | 7.49E-05 |
| ILMN_1665437 | CYP2E1 | 1.9112624 | 6.10E-08 | 4.13E-07 |
| ILMN_1787193 | CYP2S1 | -1.585601 | 3.95E-09 | 3.88E-08 |
| ILMN_1810942 | CYP3A5 | 2.6626167 | 1.92E-12 | 6.46E-11 |
| ILMN_1735816 | CYP4A11 | 1.7041399 | 1.05E-06 | 5.03E-06 |
| ILMN_1708303 | CYP4F22 | -1.8499005 | 1.42E-07 | 8.67E-07 |
| ILMN_1669802 | CYP8B1 | 1.8362398 | 3.65E-06 | 1.53E-05 |
| ILMN_2250344 | DACT1 | -1.5114938 | 2.35E-08 | 1.80E-07 |
| ILMN_2230178 | DAND5 | -2.6567359 | 1.26E-12 | 4.63E-11 |
| ILMN_1696666 | DBF4B | -1.6778648 | 3.84E-10 | 5.14E-09 |
| ILMN_1746220 | DBH | -1.6997629 | 5.16E-07 | 2.70E-06 |
| ILMN_1670000 | DCAF6 | 2.2238888 | 6.60E-08 | 4.42E-07 |
| ILMN_1811648 | DCAKD | 1.5647903 | 8.14E-09 | 7.18E-08 |
| ILMN_1729962 | DCDC5 | -1.7017489 | 1.45E-11 | 3.40E-10 |
| ILMN_2347145 | DCN | 1.569532 | 1.81E-06 | 8.21E-06 |
| ILMN_2412807 | DCTN1 | 2.561298 | 2.44E-15 | 3.33E-13 |
| ILMN_1681437 | DCXR | 2.9317326 | 6.93E-15 | 7.52E-13 |
| ILMN_1690982 | DDT | 1.8468751 | 1.77E-11 | 3.99E-10 |
| ILMN_2371590 | DDX17 | 1.8976492 | 1.16E-08 | 9.71E-08 |
| ILMN_1747303 | DDX39 | 1.5398473 | 7.55E-13 | 2.97E-11 |
| ILMN_2215382 | DDX51 | -1.5211469 | 1.87E-14 | 1.64E-12 |
| ILMN_1812976 | DDX54 | -1.6516445 | 2.88E-07 | 1.62E-06 |
| ILMN_1720838 | DECR1 | 1.5361034 | 8.78E-13 | 3.37E-11 |
| ILMN_2102721 | DEFA1B | -2.424053 | 5.79E-16 | 1.11E-13 |
| ILMN_1731598 | DEFB118 | -1.7659416 | 6.54E-08 | 4.38E-07 |
| ILMN_1706077 | DEFB125 | -1.5544935 | 2.90E-09 | 2.97E-08 |
| ILMN_2229865 | DEFB129 | -1.5435134 | 1.95E-11 | 4.32E-10 |
| ILMN_1780058 | DEGS1 | 1.8243005 | 6.32E-09 | 5.79E-08 |
| ILMN_3242008 | DEM1 | -1.8248755 | 3.86E-16 | 8.32E-14 |
| ILMN_2168952 | DENR | -1.8612897 | 1.02E-10 | 1.70E-09 |
| ILMN_2192683 | DHX37 | 1.9355354 | 4.83E-12 | 1.37E-10 |
| ILMN_1766986 | DIRC1 | -1.5463245 | 5.08E-14 | 3.59E-12 |
| ILMN_1776842 | DKFZp451A211 | -1.9844591 | 1.45E-14 | 1.34E-12 |
| ILMN_1737131 | DKFZp564N2472 | -1.6243262 | 8.85E-05 | 2.69E-04 |
| ILMN_2138622 | DKFZp686I15217 | -2.0212578 | 7.00E-12 | 1.87E-10 |
| ILMN_1736096 | DLL3 | -2.4288483 | 3.79E-13 | 1.75E-11 |
| ILMN_2162367 | DMC1 | -1.694628 | 2.20E-15 | 3.06E-13 |
| ILMN_2178771 | DMRTC2 | -2.1559868 | 1.68E-14 | 1.51E-12 |
| ILMN_2390974 | DNAJB2 | 1.6842392 | 2.66E-09 | 2.75E-08 |
| ILMN_1698258 | DNAJC8 | 2.2767233 | 5.41E-09 | 5.07E-08 |
| ILMN_2052495 | DNHL1 | -2.6067579 | 2.90E-13 | 1.39E-11 |
| ILMN_2252309 | DPP7 | 1.7823325 | 2.33E-11 | 5.02E-10 |
| ILMN_3251278 | DPPA3 | -1.7320759 | 6.52E-13 | 2.65E-11 |
| ILMN_2361810 | DRD3 | -3.081821 | 8.76E-13 | 3.37E-11 |
| ILMN_2268990 | DRD3 | -1.6010671 | 2.34E-09 | 2.45E-08 |
| ILMN_1777917 | DSCR10 | -2.2496954 | 4.74E-11 | 8.99E-10 |
| ILMN_1758066 | DSCR8 | -2.6482108 | 1.03E-11 | 2.59E-10 |
| ILMN_1682960 | DTNBP1 | -1.7500298 | 1.19E-13 | 6.82E-12 |
| ILMN_2335123 | DUB3 | -3.1131289 | 3.19E-16 | 7.29E-14 |
| ILMN_1659462 | DUSP23 | 1.6041 | 5.28E-14 | 3.67E-12 |
| ILMN_2191067 | DUX3 | -3.4352401 | 2.88E-14 | 2.29E-12 |
| ILMN_2159721 | DUX4 | -2.9488301 | 1.28E-10 | 2.07E-09 |
| ILMN_2115813 | DUX5 | -3.3701887 | 1.10E-13 | 6.44E-12 |
| ILMN_1778553 | DUXA | -1.7775346 | 3.04E-12 | 9.40E-11 |
| ILMN_2117809 | DUXAP3 | -1.8920153 | 3.28E-15 | 4.22E-13 |
| ILMN_1656196 | E2F6 | -1.9773935 | 3.83E-12 | 1.13E-10 |
| ILMN_1720287 | E4F1 | 1.5748394 | 5.97E-07 | 3.07E-06 |
| ILMN_2109708 | ECGF1 | 2.8248046 | 4.78E-13 | 2.08E-11 |
| ILMN_1653115 | ECH1 | 2.3128132 | 2.39E-18 | 1.89E-15 |
| ILMN_1671568 | ECHDC2 | 1.8934011 | 3.58E-15 | 4.50E-13 |
| ILMN_1718132 | ECHS1 | 1.6278117 | 2.47E-06 | 1.08E-05 |
| ILMN_1709719 | EDG7 | -1.6652918 | 3.08E-12 | 9.48E-11 |
| ILMN_1343291 | EEF1A1 | 1.9902615 | 7.55E-12 | 2.01E-10 |
| ILMN_2262288 | EEF1G | 2.1134211 | 5.10E-08 | 3.53E-07 |
| ILMN_2371055 | EFNA1 | 2.4476385 | 1.04E-11 | 2.60E-10 |
| ILMN_2379469 | EIF3B | 2.5603951 | 6.20E-16 | 1.19E-13 |
| ILMN_1683660 | EIF3H | 2.3820391 | 8.09E-14 | 5.11E-12 |
| ILMN_1694057 | EIF3K | 1.7970792 | 1.16E-14 | 1.13E-12 |
| ILMN_1722900 | EIF4A1 | 1.5413778 | 1.43E-09 | 1.59E-08 |
| ILMN_1685722 | EIF4A2 | 1.6578938 | 2.48E-08 | 1.89E-07 |
| ILMN_1728083 | EIF4EBP2 | 1.5197633 | 1.53E-11 | 3.55E-10 |
| ILMN_1768470 | EIF4G1 | 1.7244307 | 1.56E-12 | 5.47E-11 |
| ILMN_2279635 | EIF4G2 | 2.2346302 | 1.46E-13 | 7.99E-12 |
| ILMN_1735151 | EIF5A2 | -1.5335807 | 1.39E-08 | 1.14E-07 |
| ILMN_2202747 | ELA2A | -2.1289616 | 5.31E-10 | 6.82E-09 |
| ILMN_2411745 | EML1 | -2.0498726 | 5.46E-12 | 1.52E-10 |
| ILMN_1772644 | EML3 | 2.5745696 | 2.90E-14 | 2.30E-12 |
| ILMN_1735292 | ENKUR | -2.0009397 | 6.59E-10 | 8.22E-09 |
| ILMN_1760509 | EOMES | -1.7011093 | 1.17E-09 | 1.34E-08 |
| ILMN_2286574 | EPHA3 | -1.8270158 | 1.05E-15 | 1.74E-13 |
| ILMN_1772981 | EPN1 | 2.661516 | 4.81E-15 | 5.65E-13 |
| ILMN_2377496 | ERCC1 | 1.9265601 | 1.77E-12 | 6.00E-11 |
| ILMN_3247882 | ERI3 | 2.5939513 | 2.66E-12 | 8.37E-11 |
| ILMN_2323048 | ERP29 | 1.5123201 | 1.49E-07 | 9.05E-07 |
| ILMN_2300970 | ETFB | 2.8480776 | 1.43E-13 | 7.85E-12 |
| ILMN_1702657 | ETV3L | -2.5976812 | 9.23E-10 | 1.09E-08 |
| ILMN_1730622 | EVL | 1.7015838 | 3.28E-07 | 1.81E-06 |
| ILMN_1697735 | EWSR1 | 2.0031567 | 1.39E-12 | 5.03E-11 |
| ILMN_1670218 | EXOSC6 | 1.8605316 | 4.99E-14 | 3.54E-12 |
| ILMN_3272378 | EZR | 2.2116267 | 3.47E-10 | 4.73E-09 |
| ILMN_1671766 | F12 | 1.840788 | 2.30E-07 | 1.33E-06 |
| ILMN_1740559 | F7 | 2.6642486 | 1.20E-12 | 4.44E-11 |
| ILMN_1721559 | FABP6 | -2.4807861 | 3.62E-10 | 4.89E-09 |
| ILMN_2392261 | FABP6 | -2.2424315 | 3.86E-08 | 2.77E-07 |
| ILMN_2102951 | FAHD2A | 1.5507649 | 9.27E-13 | 3.53E-11 |
| ILMN_1803855 | FAIM2 | -1.8167027 | 2.12E-10 | 3.12E-09 |
| ILMN_2205470 | FAM153B | -2.1230749 | 5.15E-11 | 9.63E-10 |
| ILMN_1755677 | FAM158A | 1.5510725 | 1.21E-09 | 1.38E-08 |
| ILMN_3245769 | FAM163A | -1.8260979 | 6.03E-12 | 1.65E-10 |
| ILMN_3237177 | FAM175A | -1.6164492 | 6.82E-12 | 1.83E-10 |
| ILMN_1804993 | FAM178B | -1.7158895 | 4.31E-08 | 3.05E-07 |
| ILMN_3244033 | FAM183B | -2.6774642 | 1.18E-17 | 6.08E-15 |
| ILMN_1739559 | FAM187B | -2.0424994 | 1.13E-09 | 1.30E-08 |
| ILMN_1714271 | FAM19A3 | -1.5515588 | 2.35E-07 | 1.35E-06 |
| ILMN_1800705 | FAM19A4 | -1.8720551 | 2.66E-06 | 1.16E-05 |
| ILMN_2198802 | FAM22G | -2.3361714 | 4.58E-15 | 5.46E-13 |
| ILMN_1783969 | FAM24A | -1.5900107 | 4.72E-13 | 2.07E-11 |
| ILMN_2161286 | FAM40B | -1.7798776 | 3.71E-13 | 1.73E-11 |
| ILMN_1725130 | FAM50A | 2.5578576 | 1.14E-13 | 6.58E-12 |
| ILMN_1680037 | FAM65A | 1.664138 | 1.33E-09 | 1.49E-08 |
| ILMN_3242330 | FAM65C | -1.974703 | 2.92E-08 | 2.17E-07 |
| ILMN_3237067 | FAM75B | -1.7348165 | 4.53E-16 | 9.38E-14 |
| ILMN_1753482 | FAM86C | -1.8464525 | 2.24E-11 | 4.86E-10 |
| ILMN_3238173 | FAM90A7 | -1.8194721 | 2.31E-15 | 3.17E-13 |
| ILMN_1735453 | FAM98A | 1.674486 | 2.21E-10 | 3.24E-09 |
| ILMN_1657634 | FANCD2 | -1.6904994 | 9.24E-09 | 8.01E-08 |
| ILMN_1664614 | FAU | 1.6949726 | 1.97E-12 | 6.57E-11 |
| ILMN_1719205 | FBL | 2.1657691 | 1.78E-10 | 2.69E-09 |
| ILMN_1686952 | FBLN1 | -2.6256908 | 3.87E-14 | 2.90E-12 |
| ILMN_1804005 | FBP2 | -1.7255046 | 8.87E-14 | 5.50E-12 |
| ILMN_1799389 | FBXL6 | 2.1762808 | 1.08E-10 | 1.78E-09 |
| ILMN_1678404 | FBXO11 | 1.6892934 | 4.44E-12 | 1.28E-10 |
| ILMN_2383893 | FBXO24 | -1.5329317 | 2.71E-10 | 3.84E-09 |
| ILMN_2279367 | FCAR | -1.5308893 | 2.13E-11 | 4.66E-10 |
| ILMN_2144088 | FDFT1 | 1.538872 | 5.39E-06 | 2.18E-05 |
| ILMN_1802735 | FFAR3 | -1.6712651 | 8.63E-11 | 1.47E-09 |
| ILMN_1656487 | FGA | 2.0430663 | 1.73E-09 | 1.88E-08 |
| ILMN_2381945 | FGA | 2.3355631 | 2.23E-11 | 4.84E-10 |
| ILMN_1678049 | FGB | 2.0847694 | 6.13E-11 | 1.11E-09 |
| ILMN_2115011 | FGD2 | -2.3582219 | 1.36E-19 | 3.06E-16 |
| ILMN_1812517 | FGF8 | -2.1688949 | 1.10E-11 | 2.72E-10 |
| ILMN_2315044 | FGG | 2.0241376 | 2.23E-08 | 1.72E-07 |
| ILMN_2366192 | FGL1 | 1.6184485 | 3.82E-08 | 2.75E-07 |
| ILMN_1682828 | FGL1 | 1.7926897 | 7.08E-10 | 8.73E-09 |
| ILMN_2326197 | FGL1 | 1.9124514 | 3.35E-09 | 3.37E-08 |
| ILMN_2265783 | FHL2 | -2.3015392 | 3.63E-16 | 7.97E-14 |
| ILMN_1657797 | FIBP | 2.4899659 | 1.11E-13 | 6.48E-12 |
| ILMN_1676307 | FIGN | -1.9582555 | 2.43E-10 | 3.51E-09 |
| ILMN_2053377 | FLJ16734 | -1.6724967 | 8.53E-07 | 4.20E-06 |
| ILMN_1809817 | FLJ23834 | -1.5206693 | 1.29E-07 | 7.95E-07 |
| ILMN_1665260 | FLJ25996 | -2.4919139 | 1.22E-15 | 1.94E-13 |
| ILMN_1723834 | FLJ32011 | -2.1955223 | 6.93E-08 | 4.62E-07 |
| ILMN_1660007 | FLJ32658 | -2.6548581 | 1.21E-14 | 1.16E-12 |
| ILMN_2135456 | FLJ32784 | -2.4194484 | 9.57E-19 | 9.42E-16 |
| ILMN_2162454 | FLJ40113 | -2.3200999 | 1.34E-11 | 3.19E-10 |
| ILMN_3249524 | FLJ40434 | -2.842143 | 2.73E-13 | 1.34E-11 |
| ILMN_2129102 | FLJ40453 | -2.6264678 | 3.18E-17 | 1.24E-14 |
| ILMN_3243223 | FLJ41562 | -2.3297151 | 4.07E-15 | 4.97E-13 |
| ILMN_3238221 | FLJ42627 | -1.5391988 | 6.61E-11 | 1.18E-09 |
| ILMN_1732143 | FLJ42957 | -2.4854796 | 2.26E-10 | 3.31E-09 |
| ILMN_2230162 | FLJ44124 | -1.7768002 | 3.00E-19 | 4.88E-16 |
| ILMN_2149952 | FLJ45256 | -1.9325245 | 1.39E-11 | 3.29E-10 |
| ILMN_1680388 | FLJ45337 | -1.9064512 | 2.16E-12 | 7.05E-11 |
| ILMN_1675282 | FLJ45422 | -2.3763144 | 3.11E-11 | 6.30E-10 |
| ILMN_1702241 | FLJ45445 | -1.7794138 | 8.05E-08 | 5.25E-07 |
| ILMN_2194852 | FLJ46309 | -1.5676042 | 9.53E-16 | 1.61E-13 |
| ILMN_1726222 | FLOT2 | 2.058566 | 1.42E-15 | 2.20E-13 |
| ILMN_2366463 | FN1 | 2.5148684 | 3.04E-12 | 9.40E-11 |
| ILMN_1778237 | FN1 | 2.5198012 | 2.40E-10 | 3.48E-09 |
| ILMN_1682221 | FNDC8 | -1.5022047 | 2.53E-07 | 1.44E-06 |
| ILMN_1661733 | FOLR1 | -2.1644086 | 6.92E-13 | 2.76E-11 |
| ILMN_2173524 | FOXD4 | -1.5590801 | 2.03E-09 | 2.17E-08 |
| ILMN_2188390 | FOXD4L2 | -1.5813562 | 5.91E-12 | 1.62E-10 |
| ILMN_1773809 | FOXP4 | -1.8630226 | 5.23E-07 | 2.73E-06 |
| ILMN_3243065 | FRG2C | -2.197054 | 3.06E-10 | 4.25E-09 |
| ILMN_1661282 | FRMPD2 | -1.5146392 | 5.27E-06 | 2.13E-05 |
| ILMN_1739631 | FRMPD2L2 | -1.5569792 | 1.34E-10 | 2.14E-09 |
| ILMN_1661616 | FSHR | -1.9219835 | 2.98E-15 | 3.93E-13 |
| ILMN_1663569 | FTCD | 2.4220878 | 2.64E-10 | 3.76E-09 |
| ILMN_1740429 | FTL | 1.5198348 | 1.24E-06 | 5.83E-06 |
| ILMN_2309848 | FXYD5 | 1.6081177 | 1.02E-10 | 1.70E-09 |
| ILMN_1703408 | FZD3 | -1.8406168 | 3.69E-10 | 4.97E-09 |
| ILMN_1750029 | GABPA | 1.6209553 | 5.15E-15 | 5.89E-13 |
| ILMN_2161007 | GABPB2 | -1.7403598 | 2.49E-17 | 1.01E-14 |
| ILMN_2159730 | GABRB1 | -1.9663352 | 8.22E-16 | 1.49E-13 |
| ILMN_1718977 | GADD45B | 1.5724475 | 1.30E-10 | 2.09E-09 |
| ILMN_3243856 | GAGE12B | -1.6313336 | 4.42E-06 | 1.83E-05 |
| ILMN_3243851 | GAGE12C | -2.4101102 | 2.01E-07 | 1.18E-06 |
| ILMN_3243333 | GAGE12J | -3.2762576 | 1.61E-10 | 2.47E-09 |
| ILMN_2195385 | GAGE4 | -3.7659643 | 5.89E-11 | 1.07E-09 |
| ILMN_1813775 | GAK | 2.2314173 | 2.03E-09 | 2.17E-08 |
| ILMN_1697081 | GAL3ST3 | -1.8000509 | 8.25E-09 | 7.26E-08 |
| ILMN_1803194 | GALK1 | 2.245376 | 1.43E-11 | 3.37E-10 |
| ILMN_2110857 | GALR3 | -1.6764587 | 1.33E-05 | 4.89E-05 |
| ILMN_1756469 | GAMT | 2.2614223 | 6.45E-14 | 4.26E-12 |
| ILMN_1794552 | GAP43 | -2.3535401 | 1.24E-10 | 2.00E-09 |
| ILMN_1802252 | GAPDH | 1.5706116 | 2.01E-12 | 6.67E-11 |
| ILMN_2406656 | GATA3 | -1.5304563 | 1.47E-11 | 3.43E-10 |
| ILMN_1729180 | GATM | 1.8407863 | 2.28E-10 | 3.33E-09 |
| ILMN_1680320 | GBX2 | -1.6839924 | 6.88E-10 | 8.53E-09 |
| ILMN_1736162 | GC | 2.1633567 | 8.29E-13 | 3.21E-11 |
| ILMN_1725311 | GCGR | 1.5533465 | 2.46E-05 | 8.51E-05 |
| ILMN_1694472 | GCK | -1.6288908 | 8.79E-06 | 3.38E-05 |
| ILMN_1676437 | GCK | -1.5914251 | 6.03E-11 | 1.09E-09 |
| ILMN_1666545 | GCNT1 | -2.0310983 | 9.44E-13 | 3.58E-11 |
| ILMN_1760208 | GDAP1L1 | -1.6825737 | 1.23E-08 | 1.03E-07 |
| ILMN_2210111 | GDF3 | -2.4949718 | 7.69E-13 | 3.02E-11 |
| ILMN_2278908 | GGA1 | -1.5599979 | 5.19E-12 | 1.45E-10 |
| ILMN_2328874 | GH1 | -1.5663808 | 9.95E-14 | 6.00E-12 |
| ILMN_1717492 | GHSR | -2.3498491 | 1.49E-10 | 2.33E-09 |
| ILMN_1760922 | GIT2 | 1.5903501 | 1.34E-11 | 3.19E-10 |
| ILMN_1691743 | GJD3 | -1.5627694 | 1.40E-09 | 1.57E-08 |
| ILMN_1673892 | GK5 | 1.7868484 | 7.72E-09 | 6.88E-08 |
| ILMN_1796165 | GLRX5 | 1.5963208 | 1.39E-13 | 7.65E-12 |
| ILMN_1804283 | GNAS | -1.7410329 | 3.83E-09 | 3.78E-08 |
| ILMN_1769191 | GNAS | 2.1163063 | 6.90E-13 | 2.76E-11 |
| ILMN_1761113 | GNL2 | 1.9110616 | 1.19E-09 | 1.35E-08 |
| ILMN_1736238 | GNMT | 2.232719 | 6.76E-10 | 8.40E-09 |
| ILMN_2110206 | GNPTAB | -1.8120157 | 7.12E-16 | 1.33E-13 |
| ILMN_1744517 | GNS | 1.8147636 | 1.77E-10 | 2.67E-09 |
| ILMN_2186216 | GOLPH4 | -1.5861156 | 9.83E-07 | 4.76E-06 |
| ILMN_2137208 | GOLT1A | 1.7953145 | 1.13E-07 | 7.08E-07 |
| ILMN_1732269 | GP6 | -1.890782 | 1.21E-10 | 1.96E-09 |
| ILMN_1703765 | GPR12 | -2.0515044 | 1.10E-10 | 1.80E-09 |
| ILMN_1803218 | GPR139 | -1.6160753 | 3.87E-13 | 1.76E-11 |
| ILMN_1659982 | GPR152 | -3.0292352 | 5.59E-13 | 2.36E-11 |
| ILMN_1653648 | GPR63 | -1.6300019 | 2.58E-08 | 1.95E-07 |
| ILMN_2352090 | GPRC5C | 1.5099118 | 7.37E-14 | 4.77E-12 |
| ILMN_1795876 | GPS1 | 2.7799506 | 4.64E-15 | 5.50E-13 |
| ILMN_1795257 | GPT | 1.5219243 | 9.72E-09 | 8.36E-08 |
| ILMN_1684158 | GPT2 | 2.2602442 | 2.27E-09 | 2.40E-08 |
| ILMN_1726666 | GPX3 | 1.5690927 | 1.16E-14 | 1.13E-12 |
| ILMN_2378952 | GPX4 | 1.8985475 | 6.58E-09 | 5.99E-08 |
| ILMN_2385416 | GPX5 | -2.3686129 | 2.07E-12 | 6.83E-11 |
| ILMN_2385410 | GPX5 | -2.2349157 | 1.66E-12 | 5.71E-11 |
| ILMN_2060145 | GRHL2 | -1.694666 | 7.28E-09 | 6.53E-08 |
| ILMN_1659199 | GRIN1 | -1.7921817 | 1.51E-11 | 3.52E-10 |
| ILMN_2078975 | GRM3 | -3.1943496 | 2.98E-14 | 2.35E-12 |
| ILMN_2260756 | GSDMB | -1.9197759 | 2.42E-13 | 1.22E-11 |
| ILMN_1665428 | GSDMD | 1.8612309 | 2.06E-08 | 1.61E-07 |
| ILMN_2127416 | GSR | -2.5467486 | 2.18E-18 | 1.77E-15 |
| ILMN_1750790 | GSTM5 | -2.2072089 | 7.98E-11 | 1.37E-09 |
| ILMN_2227573 | GSTO1 | 1.9024139 | 1.56E-09 | 1.72E-08 |
| ILMN_1673917 | GTF2I | 2.0640048 | 1.92E-13 | 9.98E-12 |
| ILMN_2180997 | GTF2IRD2B | -1.7815631 | 9.86E-12 | 2.49E-10 |
| ILMN_1776080 | GTPBP6 | 1.9806203 | 3.80E-10 | 5.08E-09 |
| ILMN_2245686 | GYG2 | -2.302596 | 2.89E-20 | 1.06E-16 |
| ILMN_1815908 | GYPE | -2.4809057 | 1.86E-17 | 8.11E-15 |
| ILMN_1695187 | GYPE | -2.0345488 | 1.07E-13 | 6.37E-12 |
| ILMN_1757467 | H1F0 | 1.935995 | 5.79E-13 | 2.43E-11 |
| ILMN_2198823 | H6PD | -1.5471532 | 3.07E-15 | 4.02E-13 |
| ILMN_1723414 | HACL1 | 1.5601734 | 5.13E-11 | 9.60E-10 |
| ILMN_1712751 | HADHA | 1.5693813 | 6.52E-11 | 1.16E-09 |
| ILMN_1680987 | HAND1 | -2.178558 | 1.23E-10 | 1.99E-09 |
| ILMN_2358272 | HAP1 | -1.7396159 | 2.87E-08 | 2.14E-07 |
| ILMN_1763523 | HARS | 1.8033812 | 3.44E-14 | 2.63E-12 |
| ILMN_2127842 | HBA2 | 1.8151435 | 3.83E-09 | 3.79E-08 |
| ILMN_2182101 | HCN2 | -2.0099096 | 1.77E-07 | 1.05E-06 |
| ILMN_1790810 | HCN4 | -2.2710848 | 2.75E-10 | 3.89E-09 |
| ILMN_2396991 | HCST | 1.5472224 | 3.72E-10 | 5.01E-09 |
| ILMN_1726466 | HDHD3 | 1.9877708 | 3.74E-12 | 1.11E-10 |
| ILMN_1726636 | HECTD2 | -1.5761494 | 8.94E-17 | 2.85E-14 |
| ILMN_1653466 | HES4 | 2.0129048 | 3.50E-13 | 1.65E-11 |
| ILMN_1806692 | HEXB | 1.8765856 | 6.43E-09 | 5.87E-08 |
| ILMN_2324584 | HFE2 | 2.1614903 | 7.21E-09 | 6.47E-08 |
| ILMN_2198239 | HGD | 1.95505 | 5.81E-13 | 2.44E-11 |
| ILMN_1737964 | HIATL1 | 1.5444513 | 7.03E-10 | 8.69E-09 |
| ILMN_1807710 | HINT1 | 1.5663007 | 8.78E-15 | 9.16E-13 |
| ILMN_1694166 | HIST1H2AA | -1.8621514 | 8.66E-09 | 7.56E-08 |
| ILMN_2203950 | HLA-A | 1.9976465 | 6.47E-12 | 1.75E-10 |
| ILMN_2165753 | HLA-A29.1 | 1.6337274 | 1.73E-04 | 4.93E-04 |
| ILMN_1778401 | HLA-B | 2.8437982 | 8.25E-13 | 3.20E-11 |
| ILMN_1695311 | HLA-DMA | 1.6430856 | 1.12E-10 | 1.83E-09 |
| ILMN_1761733 | HLA-DMB | 1.8753481 | 7.82E-10 | 9.49E-09 |
| ILMN_1689655 | HLA-DRA | 1.661891 | 3.81E-13 | 1.75E-11 |
| ILMN_1765258 | HLA-E | 2.1363423 | 7.47E-15 | 7.95E-13 |
| ILMN_1807833 | HM13 | 1.7560169 | 4.37E-14 | 3.22E-12 |
| ILMN_1797728 | HMGCS1 | 1.5623899 | 2.41E-08 | 1.84E-07 |
| ILMN_1815203 | HMGCS2 | 1.8683268 | 6.41E-10 | 8.02E-09 |
| ILMN_2335718 | HNRNPAB | 1.8104324 | 7.34E-12 | 1.95E-10 |
| ILMN_2321451 | HNRNPD | 2.0797672 | 1.77E-10 | 2.68E-09 |
| ILMN_3179371 | HNRNPK | 1.9001408 | 5.59E-09 | 5.21E-08 |
| ILMN_2369682 | HNRPA2B1 | 2.0010082 | 2.12E-12 | 6.95E-11 |
| ILMN_1799836 | HOXA2 | -1.9831763 | 2.25E-10 | 3.30E-09 |
| ILMN_1760647 | HOXA3 | -1.9707201 | 9.57E-15 | 9.63E-13 |
| ILMN_1702479 | HOXA9 | -1.8724558 | 9.22E-09 | 7.99E-08 |
| ILMN_1718079 | HOXB1 | -1.8921583 | 7.69E-10 | 9.37E-09 |
| ILMN_1812433 | HP | 2.3019125 | 2.77E-08 | 2.07E-07 |
| ILMN_1815033 | HPCA | -1.9297702 | 8.75E-13 | 3.37E-11 |
| ILMN_1745688 | HPD | 2.1325615 | 1.79E-12 | 6.04E-11 |
| ILMN_1668593 | HPX | 1.7001057 | 1.39E-06 | 6.45E-06 |
| ILMN_1705324 | HRNBP3 | -2.3811103 | 1.28E-10 | 2.07E-09 |
| ILMN_1721429 | HS6ST3 | -2.1722385 | 5.13E-14 | 3.60E-12 |
| ILMN_2373515 | HSP90AA1 | 2.1884036 | 2.23E-10 | 3.27E-09 |
| ILMN_1789074 | HSPA1A | 1.6956184 | 6.87E-09 | 6.22E-08 |
| ILMN_2395043 | HSPA4 | 1.8369729 | 2.87E-08 | 2.14E-07 |
| ILMN_1686367 | HSPA8 | 1.5534238 | 2.70E-11 | 5.64E-10 |
| ILMN_1679209 | HSPA9 | 1.7316747 | 2.12E-11 | 4.64E-10 |
| ILMN_1674236 | HSPB1 | 1.9835462 | 6.99E-13 | 2.78E-11 |
| ILMN_2078547 | HSPC268 | -1.9818097 | 9.65E-19 | 9.42E-16 |
| ILMN_3242063 | HTA | -1.8559744 | 1.65E-12 | 5.69E-11 |
| ILMN_1715496 | HTR2A | -2.9168894 | 1.54E-18 | 1.40E-15 |
| ILMN_1739275 | HTR3C | -1.7318773 | 1.33E-09 | 1.50E-08 |
| ILMN_3241495 | HULC | 1.6750136 | 9.05E-09 | 7.86E-08 |
| ILMN_1739813 | HYAL1 | 1.6865986 | 3.15E-09 | 3.19E-08 |
| ILMN_1681296 | ICAM4 | -1.781734 | 2.41E-14 | 2.00E-12 |
| ILMN_1751753 | IDH2 | 1.5769499 | 2.20E-13 | 1.13E-11 |
| ILMN_1758626 | IDS | 1.507914 | 2.20E-08 | 1.71E-07 |
| ILMN_2058782 | IFI27 | 1.7023857 | 9.08E-08 | 5.84E-07 |
| ILMN_1801246 | IFITM1 | 1.6672147 | 7.30E-09 | 6.54E-08 |
| ILMN_1673352 | IFITM2 | 1.7794612 | 1.46E-11 | 3.42E-10 |
| ILMN_1805750 | IFITM3 | 2.0128928 | 5.83E-13 | 2.44E-11 |
| ILMN_1744635 | IGDCC3 | -2.8878216 | 1.85E-12 | 6.25E-11 |
| ILMN_2387385 | IGFBP1 | 1.5373032 | 1.72E-04 | 4.90E-04 |
| ILMN_1679897 | IGFL3 | -3.3446374 | 9.78E-14 | 5.93E-12 |
| ILMN_1707308 | IKBKG | 1.5274839 | 5.36E-09 | 5.03E-08 |
| ILMN_2073307 | IL10 | -1.7937783 | 5.07E-10 | 6.56E-09 |
| ILMN_2188247 | IL17F | -2.4087393 | 1.25E-11 | 3.03E-10 |
| ILMN_2407851 | IL17RD | -1.5015372 | 1.56E-12 | 5.47E-11 |
| ILMN_1778457 | IL18 | -1.5892628 | 8.78E-12 | 2.27E-10 |
| ILMN_2160428 | IL1RAPL1 | -2.3242616 | 1.08E-13 | 6.40E-12 |
| ILMN_1715603 | IL23A | -1.792156 | 7.81E-14 | 5.00E-12 |
| ILMN_1662302 | IL28A | -1.7083625 | 4.92E-07 | 2.59E-06 |
| ILMN_2149624 | IL29 | -1.6963731 | 2.36E-14 | 1.97E-12 |
| ILMN_1683774 | IL2RA | -1.6551895 | 4.39E-09 | 4.25E-08 |
| ILMN_2368530 | IL32 | 2.4301276 | 8.75E-12 | 2.27E-10 |
| ILMN_1794686 | IL9R | -1.5490922 | 7.95E-12 | 2.10E-10 |
| ILMN_1769451 | ILVBL | 1.5735675 | 2.42E-09 | 2.53E-08 |
| ILMN_1756070 | INSC | -2.0219956 | 8.40E-14 | 5.26E-12 |
| ILMN_2392286 | IP6K1 | 1.6126257 | 3.13E-14 | 2.46E-12 |
| ILMN_1654598 | IQCA1 | -1.6637492 | 3.83E-15 | 4.75E-13 |
| ILMN_1681576 | IQCF2 | -1.7272631 | 1.41E-09 | 1.58E-08 |
| ILMN_1659913 | ISG20 | 1.5210086 | 1.16E-08 | 9.73E-08 |
| ILMN_1792679 | ITGA5 | 1.8111199 | 2.99E-10 | 4.16E-09 |
| ILMN_2383934 | ITGB1 | 2.1766524 | 6.50E-13 | 2.65E-11 |
| ILMN_1798373 | ITPRIPL1 | -2.7145065 | 5.94E-14 | 4.03E-12 |
| ILMN_1687921 | JMJD8 | 2.7742484 | 3.39E-15 | 4.30E-13 |
| ILMN_1806023 | JUN | 1.5563748 | 3.63E-08 | 2.63E-07 |
| ILMN_1774565 | KAAG1 | -1.8929355 | 6.35E-12 | 1.72E-10 |
| ILMN_1692156 | KCNC2 | -2.0533963 | 1.29E-11 | 3.10E-10 |
| ILMN_1704063 | KCNH3 | -2.0883054 | 1.96E-12 | 6.54E-11 |
| ILMN_1729963 | KCNH5 | -2.7203051 | 8.55E-12 | 2.22E-10 |
| ILMN_1700199 | KCNH5 | -1.5297376 | 1.02E-09 | 1.19E-08 |
| ILMN_1709847 | KCNJ13 | -2.0159923 | 1.25E-12 | 4.59E-11 |
| ILMN_1711988 | KCNK12 | -2.608776 | 5.78E-13 | 2.43E-11 |
| ILMN_1685636 | KCNN2 | -1.8764081 | 1.06E-11 | 2.65E-10 |
| ILMN_1703500 | KCNQ2 | -2.424401 | 6.48E-10 | 8.09E-09 |
| ILMN_1666776 | KCNQ2 | -1.6341917 | 2.00E-10 | 2.98E-09 |
| ILMN_1761903 | KCNS1 | -1.71279 | 2.85E-11 | 5.88E-10 |
| ILMN_1705562 | KIAA0802 | -1.557663 | 9.94E-10 | 1.17E-08 |
| ILMN_3187852 | KIAA1310 | 1.5549092 | 1.74E-07 | 1.03E-06 |
| ILMN_1727526 | KIAA1407 | -1.5267371 | 2.15E-08 | 1.67E-07 |
| ILMN_2415979 | KIAA1751 | -1.5403534 | 6.78E-13 | 2.74E-11 |
| ILMN_1666625 | KIF17 | -1.5963545 | 9.03E-10 | 1.07E-08 |
| ILMN_1735552 | KIF1B | 1.7929901 | 2.89E-10 | 4.05E-09 |
| ILMN_1667232 | KIR2DL3 | -2.8435981 | 4.31E-11 | 8.27E-10 |
| ILMN_1691803 | KIR2DS5 | -2.3742774 | 6.73E-15 | 7.33E-13 |
| ILMN_2131828 | KIR3DL1 | -1.8465137 | 3.11E-13 | 1.48E-11 |
| ILMN_2190842 | KIR3DL2 | -2.7451856 | 4.76E-13 | 2.08E-11 |
| ILMN_2082593 | KIR3DL3 | -2.06404 | 2.17E-15 | 3.04E-13 |
| ILMN_1674541 | KIRREL3 | -1.7020788 | 1.52E-07 | 9.19E-07 |
| ILMN_1735014 | KLF6 | 1.5032335 | 5.67E-10 | 7.21E-09 |
| ILMN_1730940 | KLHDC3 | 2.2896842 | 4.56E-12 | 1.31E-10 |
| ILMN_1666191 | KLK13 | -1.8793458 | 2.79E-14 | 2.24E-12 |
| ILMN_1750850 | KLK14 | -1.8279682 | 6.11E-12 | 1.67E-10 |
| ILMN_1663787 | KLK3 | -2.0628109 | 3.51E-10 | 4.77E-09 |
| ILMN_2395496 | KLK7 | -1.5294022 | 3.17E-09 | 3.20E-08 |
| ILMN_1705252 | KLK8 | -1.8953265 | 3.94E-11 | 7.68E-10 |
| ILMN_1753729 | KNG1 | 2.149327 | 1.33E-10 | 2.12E-09 |
| ILMN_1780649 | KPRP | -2.6097931 | 1.55E-12 | 5.46E-11 |
| ILMN_1721218 | KRT13 | -1.8693227 | 2.81E-11 | 5.82E-10 |
| ILMN_2228162 | KRT16 | -1.9291681 | 3.26E-12 | 9.96E-11 |
| ILMN_1680828 | KRT27 | -1.7665137 | 1.11E-10 | 1.81E-09 |
| ILMN_1695621 | KRT3 | -2.1400334 | 7.16E-14 | 4.67E-12 |
| ILMN_1801632 | KRT5 | -1.7681991 | 3.48E-12 | 1.05E-10 |
| ILMN_1754576 | KRT6C | -1.6957689 | 3.58E-11 | 7.08E-10 |
| ILMN_1716136 | KRT71 | -1.8463981 | 1.35E-11 | 3.21E-10 |
| ILMN_1695812 | KRT72 | -1.560826 | 1.46E-12 | 5.22E-11 |
| ILMN_1721247 | KRT75 | -1.5999762 | 7.88E-09 | 6.99E-08 |
| ILMN_1765072 | KRT85 | -1.5046325 | 6.22E-10 | 7.81E-09 |
| ILMN_2135306 | KRTAP10-10 | -1.7421188 | 2.43E-08 | 1.86E-07 |
| ILMN_1776412 | KRTAP10-11 | -1.5479698 | 5.06E-06 | 2.06E-05 |
| ILMN_2206732 | KRTAP10-3 | -1.8147097 | 4.00E-11 | 7.77E-10 |
| ILMN_2209260 | KRTAP1-3 | -1.6239522 | 4.54E-11 | 8.64E-10 |
| ILMN_1791951 | KRTAP13-1 | -1.9577782 | 3.50E-11 | 6.96E-10 |
| ILMN_1666823 | KRTAP23-1 | -1.6957831 | 2.30E-09 | 2.42E-08 |
| ILMN_1652439 | KRTAP3-1 | -1.8073289 | 1.12E-08 | 9.41E-08 |
| ILMN_2108025 | KRTAP3-2 | -2.6471653 | 6.35E-12 | 1.72E-10 |
| ILMN_1703708 | KRTAP5-10 | -1.6623375 | 1.13E-07 | 7.11E-07 |
| ILMN_2197990 | KRTAP5-8 | -1.5372973 | 2.44E-10 | 3.52E-09 |
| ILMN_1694419 | KRTAP8-1 | -2.3683354 | 6.76E-11 | 1.20E-09 |
| ILMN_1658802 | KRTCAP2 | 2.119869 | 3.30E-13 | 1.56E-11 |
| ILMN_1701581 | LAD1 | -1.6451603 | 1.58E-08 | 1.27E-07 |
| ILMN_1782389 | LAD1 | 1.8448866 | 8.66E-09 | 7.57E-08 |
| ILMN_1728255 | LALBA | -2.0081663 | 3.26E-14 | 2.53E-12 |
| ILMN_1696434 | LAMA1 | -1.5842472 | 3.67E-08 | 2.66E-07 |
| ILMN_1665657 | LCA5L | -2.5331111 | 1.94E-15 | 2.80E-13 |
| ILMN_1815102 | LCAT | 2.0795018 | 1.81E-10 | 2.73E-09 |
| ILMN_3238649 | LCE6A | -2.3611241 | 1.73E-13 | 9.26E-12 |
| ILMN_1692223 | LCN2 | 1.767307 | 1.53E-08 | 1.24E-07 |
| ILMN_3249748 | LDHA | 1.7783656 | 1.63E-07 | 9.76E-07 |
| ILMN_2234956 | LEPR | 1.5209755 | 7.28E-08 | 4.80E-07 |
| ILMN_1754266 | LHX9 | -1.5623416 | 6.72E-10 | 8.37E-09 |
| ILMN_1661631 | LILRA3 | -1.5226783 | 4.98E-06 | 2.03E-05 |
| ILMN_2058841 | LILRA6 | -2.2566208 | 2.77E-18 | 1.99E-15 |
| ILMN_2316974 | LILRB1 | -1.743582 | 1.24E-21 | 1.82E-17 |
| ILMN_2183687 | LIME1 | 2.2724265 | 9.05E-12 | 2.33E-10 |
| ILMN_1714880 | LIMS3 | -1.8510851 | 4.71E-17 | 1.66E-14 |
| ILMN_1748903 | LIN28 | -2.6083908 | 1.80E-19 | 3.11E-16 |
| ILMN_1695978 | LINGO2 | -1.7523645 | 9.60E-14 | 5.85E-12 |
| ILMN_1652866 | LMX1B | -2.1386172 | 4.69E-16 | 9.54E-14 |
| ILMN_1733559 | LOC100008589 | 1.5730152 | 5.60E-06 | 2.25E-05 |
| ILMN_3262250 | LOC100125556 | -1.5654004 | 1.39E-11 | 3.28E-10 |
| ILMN_3247452 | LOC100128731 | 1.9124682 | 2.01E-11 | 4.43E-10 |
| ILMN_3294106 | LOC100190938 | -1.8115024 | 1.73E-16 | 4.57E-14 |
| ILMN_1665740 | LOC149950 | -1.5483044 | 1.82E-13 | 9.66E-12 |
| ILMN_1735172 | LOC151121 | -1.7678004 | 1.13E-08 | 9.50E-08 |
| ILMN_2135740 | LOC152667 | -2.5287861 | 1.48E-12 | 5.29E-11 |
| ILMN_3246453 | LOC162632 | -1.9730562 | 1.59E-12 | 5.52E-11 |
| ILMN_2413377 | LOC222967 | -2.0961335 | 2.56E-09 | 2.66E-08 |
| ILMN_2297662 | LOC222967 | -1.6376322 | 4.82E-14 | 3.45E-12 |
| ILMN_3206804 | LOC255167 | -2.3535876 | 8.14E-18 | 4.59E-15 |
| ILMN_2096442 | LOC260339 | -2.3614052 | 1.67E-16 | 4.46E-14 |
| ILMN_1745409 | LOC340529 | -1.6093255 | 2.94E-10 | 4.11E-09 |
| ILMN_3244755 | LOC342994 | -1.5697616 | 2.80E-17 | 1.11E-14 |
| ILMN_1792437 | LOC348174 | -2.1578497 | 1.80E-15 | 2.66E-13 |
| ILMN_1733757 | LOC374395 | 3.1573926 | 1.46E-12 | 5.22E-11 |
| ILMN_2057408 | LOC388503 | 1.9882563 | 4.19E-07 | 2.24E-06 |
| ILMN_2170625 | LOC389286 | -1.7753489 | 3.99E-10 | 5.31E-09 |
| ILMN_1659851 | LOC389832 | -1.5569384 | 1.11E-13 | 6.48E-12 |
| ILMN_1701314 | LOC399900 | -1.5678024 | 4.23E-09 | 4.13E-08 |
| ILMN_2046856 | LOC401019 | 1.6608064 | 9.89E-11 | 1.65E-09 |
| ILMN_2125590 | LOC401252 | -2.1136353 | 4.81E-12 | 1.36E-10 |
| ILMN_1784493 | LOC401286 | -1.5309988 | 2.73E-08 | 2.04E-07 |
| ILMN_1664289 | LOC440570 | -3.2380824 | 1.81E-14 | 1.61E-12 |
| ILMN_1675258 | LOC441268 | -2.0976792 | 1.42E-13 | 7.80E-12 |
| ILMN_1665210 | LOC441426 | -2.2178585 | 3.33E-16 | 7.51E-14 |
| ILMN_1719826 | LOC441956 | -2.2071615 | 5.23E-11 | 9.75E-10 |
| ILMN_1762170 | LOC441956 | -2.0361989 | 7.35E-12 | 1.96E-10 |
| ILMN_1749341 | LOC554226 | -2.298345 | 2.14E-16 | 5.32E-14 |
| ILMN_1757747 | LOC554235 | 1.8089148 | 7.68E-08 | 5.04E-07 |
| ILMN_3237703 | LOC645431 | -2.7408251 | 2.72E-16 | 6.43E-14 |
| ILMN_3247256 | LOC646996 | -1.9909868 | 1.83E-16 | 4.74E-14 |
| ILMN_1797544 | LOC648148 | -2.3961897 | 5.91E-11 | 1.07E-09 |
| ILMN_1652170 | LOC649159 | -2.8406325 | 1.88E-11 | 4.19E-10 |
| ILMN_2211910 | LOC649159 | -1.7569145 | 4.00E-12 | 1.16E-10 |
| ILMN_3238130 | LOC653544 | -2.5031372 | 1.32E-10 | 2.11E-09 |
| ILMN_3235234 | LOC727924 | -1.9899127 | 8.97E-16 | 1.55E-13 |
| ILMN_3242226 | LOC729384 | -3.0431653 | 1.42E-15 | 2.20E-13 |
| ILMN_1710410 | LOC730413 | -1.7001583 | 8.16E-10 | 9.85E-09 |
| ILMN_2219618 | LOC90586 | -2.1692039 | 6.87E-19 | 8.64E-16 |
| ILMN_1718387 | LOR | -2.0841188 | 3.56E-11 | 7.06E-10 |
| ILMN_1656802 | LOXL2 | -1.6403375 | 5.98E-11 | 1.08E-09 |
| ILMN_2179083 | LOXL4 | 1.5592253 | 3.00E-07 | 1.67E-06 |
| ILMN_1670767 | LPO | -1.6037132 | 3.92E-11 | 7.65E-10 |
| ILMN_1758924 | LRFN2 | -1.6779097 | 1.00E-07 | 6.38E-07 |
| ILMN_1684339 | LRIT1 | -1.6700465 | 5.69E-10 | 7.23E-09 |
| ILMN_2127605 | LRP3 | 2.6862555 | 3.23E-16 | 7.32E-14 |
| ILMN_1675268 | LRP4 | -1.7122785 | 2.87E-07 | 1.61E-06 |
| ILMN_2393693 | LRRC37A4 | -1.5824171 | 2.92E-12 | 9.07E-11 |
| ILMN_3245600 | LRRC37B2 | -2.1320093 | 2.29E-15 | 3.17E-13 |
| ILMN_1697054 | LRRN4CL | -1.5246664 | 4.54E-07 | 2.41E-06 |
| ILMN_1811102 | LRSAM1 | 1.6989954 | 2.20E-11 | 4.79E-10 |
| ILMN_1702698 | LSM11 | -1.5944075 | 2.25E-10 | 3.30E-09 |
| ILMN_1777725 | LSM14B | 1.891394 | 1.26E-13 | 7.11E-12 |
| ILMN_1763467 | LY6G5C | -2.1949846 | 9.33E-12 | 2.39E-10 |
| ILMN_1682935 | LYPLAL1 | 1.6349154 | 1.12E-13 | 6.50E-12 |
| ILMN_2162972 | LYZ | 1.71484 | 1.02E-06 | 4.91E-06 |
| ILMN_1815205 | LYZ | 2.389298 | 4.07E-10 | 5.40E-09 |
| ILMN_1789162 | MAB21L2 | -1.6739058 | 3.71E-08 | 2.68E-07 |
| ILMN_1737935 | MACF1 | 2.0171511 | 1.17E-12 | 4.35E-11 |
| ILMN_1719543 | MAF | 1.8127594 | 1.46E-10 | 2.28E-09 |
| ILMN_1803773 | MAG | -2.0010312 | 8.42E-12 | 2.19E-10 |
| ILMN_1741430 | MAGEA10 | -2.9896668 | 4.39E-13 | 1.96E-11 |
| ILMN_2320330 | MAL | -1.9785828 | 2.83E-10 | 3.98E-09 |
| ILMN_2141650 | MALAT1 | -2.0860734 | 1.74E-15 | 2.61E-13 |
| ILMN_1663640 | MAOA | 1.9155378 | 5.34E-08 | 3.67E-07 |
| ILMN_1776188 | MAP1LC3A | 1.9061343 | 5.64E-14 | 3.88E-12 |
| ILMN_1711331 | MAP1LC3C | -2.6684231 | 1.37E-13 | 7.63E-12 |
| ILMN_1708064 | MAP4 | 1.5162987 | 3.33E-09 | 3.35E-08 |
| ILMN_1723625 | MAP4K2 | 1.6274611 | 2.32E-10 | 3.39E-09 |
| ILMN_1788002 | MAPK14 | 1.9857371 | 2.85E-12 | 8.91E-11 |
| ILMN_1667260 | MAPK3 | 1.6787318 | 8.11E-09 | 7.17E-08 |
| ILMN_1800049 | MAPT | -1.8396409 | 8.15E-14 | 5.11E-12 |
| ILMN_3236231 | MBD3L5 | -1.7942063 | 6.93E-13 | 2.77E-11 |
| ILMN_1667754 | MC3R | -1.9258027 | 3.88E-14 | 2.90E-12 |
| ILMN_1798581 | MCM8 | -1.8530497 | 1.08E-11 | 2.68E-10 |
| ILMN_1793386 | MED12 | 1.5688529 | 4.17E-13 | 1.88E-11 |
| ILMN_1800451 | MED16 | 2.5219418 | 1.76E-12 | 6.00E-11 |
| ILMN_1811823 | MED25 | 1.8270165 | 2.11E-11 | 4.62E-10 |
| ILMN_1654543 | MED6 | 1.6936066 | 7.33E-10 | 8.99E-09 |
| ILMN_1810254 | MEI1 | -1.7152386 | 5.79E-09 | 5.38E-08 |
| ILMN_2277877 | MEN1 | -1.814343 | 6.25E-08 | 4.22E-07 |
| ILMN_1656285 | METTL7A | 1.593321 | 5.43E-09 | 5.08E-08 |
| ILMN_1756071 | MFGE8 | 1.7630274 | 3.30E-07 | 1.81E-06 |
| ILMN_1692477 | MGAT5B | -1.6252961 | 4.65E-10 | 6.07E-09 |
| ILMN_2147251 | MGC10997 | -1.8951175 | 3.88E-12 | 1.14E-10 |
| ILMN_2050434 | MGC16703 | -1.5722112 | 2.10E-11 | 4.62E-10 |
| ILMN_1776121 | MGC42367 | -1.5290323 | 2.89E-10 | 4.05E-09 |
| ILMN_1795639 | MGMT | 1.6837821 | 2.64E-11 | 5.52E-10 |
| ILMN_2355168 | MGST1 | 2.3087938 | 9.92E-14 | 5.99E-12 |
| ILMN_1781952 | MGST1 | 2.3173726 | 3.66E-08 | 2.65E-07 |
| ILMN_1783843 | MIIP | 2.4496469 | 7.36E-15 | 7.86E-13 |
| ILMN_1674353 | MIOX | -2.7515235 | 1.04E-13 | 6.19E-12 |
| ILMN_3310673 | MIR1208 | -1.5360065 | 1.17E-09 | 1.34E-08 |
| ILMN_3308118 | MIR125B1 | -2.2487564 | 4.35E-17 | 1.57E-14 |
| ILMN_3308535 | MIR1289-2 | -1.769311 | 2.16E-12 | 7.05E-11 |
| ILMN_3310491 | MIR1978 | -1.5523808 | 2.35E-09 | 2.46E-08 |
| ILMN_3308713 | MIR370 | -1.9668351 | 1.43E-11 | 3.36E-10 |
| ILMN_3308540 | MIR548I1 | -2.114796 | 1.01E-10 | 1.68E-09 |
| ILMN_3311115 | MIR663B | -2.2051282 | 1.70E-12 | 5.81E-11 |
| ILMN_3308550 | MIR933 | -1.5382601 | 4.57E-07 | 2.42E-06 |
| ILMN_1663793 | MIST | -2.5972213 | 2.32E-14 | 1.97E-12 |
| ILMN_1668969 | MIXL1 | -1.7264413 | 2.25E-11 | 4.87E-10 |
| ILMN_1734827 | MKI67 | -1.802838 | 1.00E-07 | 6.37E-07 |
| ILMN_1693987 | MLXIP | 1.5273924 | 2.74E-13 | 1.34E-11 |
| ILMN_2399919 | MLXIPL | 1.6681588 | 4.13E-07 | 2.21E-06 |
| ILMN_1660114 | MMRN1 | -1.871515 | 5.35E-11 | 9.96E-10 |
| ILMN_2323801 | MOCS1 | 2.3404899 | 1.01E-13 | 6.06E-12 |
| ILMN_1654722 | MPV17L | -2.0789967 | 6.31E-13 | 2.60E-11 |
| ILMN_2055165 | MRFAP1 | 1.5338206 | 1.51E-08 | 1.22E-07 |
| ILMN_1699603 | MRPL12 | 1.5232852 | 3.05E-08 | 2.26E-07 |
| ILMN_2041327 | MRPL37 | 2.3208832 | 7.51E-14 | 4.84E-12 |
| ILMN_2141523 | MRPL44 | -1.5559147 | 9.41E-17 | 2.93E-14 |
| ILMN_2371964 | MRPS12 | 2.0744634 | 1.68E-14 | 1.51E-12 |
| ILMN_1723874 | MRPS6 | 1.6489418 | 2.94E-10 | 4.10E-09 |
| ILMN_2077130 | MSH3 | -1.6491156 | 1.82E-16 | 4.74E-14 |
| ILMN_1657977 | MSRB2 | 2.0588017 | 7.35E-10 | 9.00E-09 |
| ILMN_1688154 | MST1R | -1.6437498 | 4.70E-09 | 4.50E-08 |
| ILMN_1715401 | MT1G | 1.6676252 | 6.94E-08 | 4.62E-07 |
| ILMN_2175831 | MTFMT | -1.9778705 | 1.37E-16 | 4.01E-14 |
| ILMN_1785324 | MTHFD1 | 2.752059 | 3.40E-13 | 1.60E-11 |
| ILMN_2293322 | MTHFD2 | -2.4960042 | 1.09E-13 | 6.43E-12 |
| ILMN_1810978 | MUCL1 | -1.8640426 | 1.04E-09 | 1.21E-08 |
| ILMN_1724805 | MUPCDH | 1.5623455 | 4.07E-12 | 1.18E-10 |
| ILMN_1756541 | MXD4 | 1.9202037 | 4.11E-09 | 4.02E-08 |
| ILMN_1752075 | MYBPC1 | -1.7643247 | 1.09E-11 | 2.70E-10 |
| ILMN_1722872 | MYH9 | 2.2983781 | 8.75E-16 | 1.53E-13 |
| ILMN_1675848 | MYL12A | 1.9468397 | 6.05E-08 | 4.10E-07 |
| ILMN_1798992 | MYL3 | -1.7286935 | 4.51E-07 | 2.39E-06 |
| ILMN_2326071 | MYL6 | 1.5418546 | 1.59E-08 | 1.28E-07 |
| ILMN_1809013 | MYL6 | 1.79084 | 1.79E-09 | 1.94E-08 |
| ILMN_1704245 | MYO18B | -1.501937 | 1.50E-11 | 3.50E-10 |
| ILMN_1680273 | MYOCD | -1.8650004 | 2.31E-08 | 1.78E-07 |
| ILMN_1731157 | MYOZ1 | -1.6471183 | 2.13E-08 | 1.66E-07 |
| ILMN_2222101 | N4BP2 | -1.5369566 | 3.18E-15 | 4.12E-13 |
| ILMN_2315569 | N6AMT1 | -1.6615962 | 1.26E-09 | 1.42E-08 |
| ILMN_2090949 | NAG18 | -1.5113589 | 4.30E-10 | 5.67E-09 |
| ILMN_1753111 | NAMPT | 2.393945 | 8.37E-16 | 1.50E-13 |
| ILMN_1694414 | NANOS2 | -2.0430957 | 1.48E-07 | 8.98E-07 |
| ILMN_1710752 | NAPRT1 | 2.1894958 | 6.38E-13 | 2.62E-11 |
| ILMN_1689097 | NAT5 | 1.9021424 | 5.72E-10 | 7.26E-09 |
| ILMN_2106725 | NCF1B | -1.668462 | 4.32E-12 | 1.25E-10 |
| ILMN_3237632 | NCLN | 1.6247613 | 1.88E-14 | 1.64E-12 |
| ILMN_1773906 | NCOA4 | 1.8346008 | 8.14E-14 | 5.11E-12 |
| ILMN_1751452 | NDFIP1 | 1.5655505 | 2.37E-11 | 5.09E-10 |
| ILMN_1784641 | NDUFA3 | 1.7064171 | 3.43E-04 | 9.11E-04 |
| ILMN_2179018 | NDUFAB1 | 1.540897 | 2.62E-08 | 1.98E-07 |
| ILMN_1813604 | NDUFB7 | 1.9891359 | 5.30E-08 | 3.65E-07 |
| ILMN_1661170 | NDUFB8 | 2.4685843 | 9.05E-11 | 1.53E-09 |
| ILMN_1733603 | NDUFC1 | 1.7369327 | 3.84E-13 | 1.76E-11 |
| ILMN_1669966 | NDUFS7 | 2.8119249 | 1.46E-16 | 4.10E-14 |
| ILMN_2058070 | NEDD8 | 2.2133366 | 1.71E-09 | 1.87E-08 |
| ILMN_1659086 | NEFL | -2.7151104 | 3.67E-11 | 7.23E-10 |
| ILMN_2253145 | NEK3 | -1.7342873 | 9.35E-06 | 3.57E-05 |
| ILMN_2142554 | NENF | 1.8015361 | 8.57E-10 | 1.03E-08 |
| ILMN_1677608 | NEUROD4 | -1.8422108 | 4.78E-16 | 9.65E-14 |
| ILMN_1805457 | NHLH1 | -1.6330992 | 1.52E-09 | 1.68E-08 |
| ILMN_1807211 | NICN1 | 1.8246605 | 4.31E-13 | 1.94E-11 |
| ILMN_2394841 | NKX2-1 | -3.1188531 | 4.45E-10 | 5.84E-09 |
| ILMN_1658632 | NLRP7 | -1.5589006 | 6.13E-14 | 4.14E-12 |
| ILMN_2075794 | NLRP8 | -1.6515786 | 1.38E-17 | 6.74E-15 |
| ILMN_1800634 | NME4 | 1.5391322 | 3.42E-18 | 2.33E-15 |
| ILMN_1784783 | NME5 | -1.5449159 | 2.25E-11 | 4.87E-10 |
| ILMN_1742968 | NMNAT2 | -1.5094727 | 7.13E-15 | 7.65E-13 |
| ILMN_1691413 | NNAT | -2.125508 | 1.46E-14 | 1.35E-12 |
| ILMN_1652287 | NOG | -2.8463217 | 1.33E-15 | 2.09E-13 |
| ILMN_1803853 | NOL7 | 1.8529336 | 1.30E-10 | 2.08E-09 |
| ILMN_1740976 | NONO | 1.7299995 | 1.08E-14 | 1.06E-12 |
| ILMN_1803030 | NOS1 | -1.801031 | 1.31E-10 | 2.10E-09 |
| ILMN_1658926 | NOTCH3 | 1.5390803 | 5.17E-09 | 4.89E-08 |
| ILMN_1716678 | NPC2 | 2.146494 | 1.88E-11 | 4.20E-10 |
| ILMN_1783336 | NPFFR1 | -1.6500052 | 5.12E-08 | 3.54E-07 |
| ILMN_1654534 | NPSR1 | -1.689145 | 8.25E-10 | 9.94E-09 |
| ILMN_2336982 | NPTN | 1.6491223 | 1.54E-08 | 1.25E-07 |
| ILMN_1763750 | NPTX1 | -2.9920451 | 1.04E-19 | 2.73E-16 |
| ILMN_1814022 | NR1H3 | 2.1396627 | 7.62E-14 | 4.89E-12 |
| ILMN_1733248 | NRBP2 | 1.7263067 | 1.33E-10 | 2.12E-09 |
| ILMN_1670809 | NRM | -1.6428832 | 5.84E-12 | 1.61E-10 |
| ILMN_1692163 | NSDHL | 1.5051484 | 1.22E-06 | 5.73E-06 |
| ILMN_1806432 | NT5C | 2.2177352 | 5.65E-16 | 1.10E-13 |
| ILMN_1749827 | NT5C1B | -1.8166242 | 4.05E-12 | 1.18E-10 |
| ILMN_1806448 | NTNG2 | -1.8931846 | 3.55E-07 | 1.93E-06 |
| ILMN_2326075 | NTRK1 | -1.5529266 | 2.66E-08 | 2.00E-07 |
| ILMN_1722634 | NUCB1 | 2.3178793 | 2.43E-14 | 2.00E-12 |
| ILMN_1655913 | NUCB2 | 1.600191 | 2.83E-11 | 5.85E-10 |
| ILMN_3251404 | NUCKS1 | 2.4293597 | 2.47E-12 | 7.88E-11 |
| ILMN_2097546 | NUDC | 2.0715255 | 2.57E-12 | 8.15E-11 |
| ILMN_3251356 | NUDT4P1 | -1.573422 | 1.96E-10 | 2.92E-09 |
| ILMN_1741055 | NUP210L | -1.7476582 | 4.85E-07 | 2.55E-06 |
| ILMN_3236858 | NYNRIN | -1.7399388 | 1.82E-07 | 1.07E-06 |
| ILMN_1668345 | OAF | 1.7691184 | 1.18E-07 | 7.40E-07 |
| ILMN_1773080 | OAZ1 | 2.0288897 | 4.03E-11 | 7.81E-10 |
| ILMN_3245811 | ODF3 | -2.0718414 | 1.17E-09 | 1.34E-08 |
| ILMN_2336094 | ODZ3 | -3.5098603 | 6.42E-15 | 7.10E-13 |
| ILMN_2352609 | OGG1 | 2.1542321 | 1.33E-16 | 3.95E-14 |
| ILMN_1795963 | OKL38 | 2.7516828 | 1.87E-13 | 9.82E-12 |
| ILMN_1711030 | OPLAH | 2.003471 | 6.75E-10 | 8.39E-09 |
| ILMN_1677153 | OPN1LW | -1.8471477 | 1.98E-14 | 1.72E-12 |
| ILMN_1689129 | OPRM1 | -1.5700183 | 1.13E-11 | 2.78E-10 |
| ILMN_1731717 | OR10G8 | -2.6281705 | 3.55E-12 | 1.07E-10 |
| ILMN_1653073 | OR10Q1 | -1.5247973 | 4.67E-08 | 3.27E-07 |
| ILMN_1759087 | OR10V1 | -2.734196 | 6.68E-14 | 4.38E-12 |
| ILMN_2342651 | OR11H12 | -3.2060119 | 3.92E-15 | 4.80E-13 |
| ILMN_1679870 | OR11H12 | -2.1647474 | 1.49E-14 | 1.37E-12 |
| ILMN_1767244 | OR13A1 | -1.6538501 | 4.48E-12 | 1.29E-10 |
| ILMN_1753884 | OR13J1 | -2.2357377 | 2.65E-08 | 2.00E-07 |
| ILMN_1674997 | OR14C36 | -1.9658691 | 1.91E-15 | 2.77E-13 |
| ILMN_1813390 | OR1C1 | -1.5202955 | 5.60E-08 | 3.83E-07 |
| ILMN_1705077 | OR1J1 | -1.931545 | 2.25E-13 | 1.16E-11 |
| ILMN_1716249 | OR1K1 | -1.6037893 | 1.40E-10 | 2.21E-09 |
| ILMN_2067813 | OR1L4 | -1.9875388 | 5.27E-13 | 2.25E-11 |
| ILMN_1766401 | OR1M1 | -1.8237428 | 1.32E-09 | 1.49E-08 |
| ILMN_2074432 | OR2A1 | -2.194693 | 3.15E-14 | 2.46E-12 |
| ILMN_1714891 | OR2A14 | -1.5327759 | 8.63E-10 | 1.03E-08 |
| ILMN_1732024 | OR2A5 | -1.8915023 | 1.29E-14 | 1.22E-12 |
| ILMN_1749168 | OR2AE1 | -1.5019718 | 1.72E-10 | 2.61E-09 |
| ILMN_1779412 | OR2AT4 | -1.8451652 | 6.59E-10 | 8.22E-09 |
| ILMN_1799282 | OR2B11 | -2.5097015 | 2.62E-11 | 5.50E-10 |
| ILMN_1772179 | OR2G2 | -2.6109706 | 1.16E-15 | 1.88E-13 |
| ILMN_1793403 | OR2G3 | -2.1461388 | 4.16E-14 | 3.09E-12 |
| ILMN_1766087 | OR2G6 | -2.1006397 | 2.54E-16 | 6.21E-14 |
| ILMN_1804722 | OR2J3 | -1.6869347 | 1.25E-14 | 1.20E-12 |
| ILMN_1701454 | OR2L13 | -1.9385913 | 1.67E-09 | 1.84E-08 |
| ILMN_2129658 | OR2L8 | -1.7477718 | 9.59E-12 | 2.43E-10 |
| ILMN_1795920 | OR2M4 | -2.2991017 | 1.46E-11 | 3.41E-10 |
| ILMN_1656221 | OR2T27 | -1.9168042 | 2.86E-13 | 1.38E-11 |
| ILMN_1735906 | OR2T3 | -1.5149654 | 1.61E-11 | 3.70E-10 |
| ILMN_1813776 | OR4D6 | -1.8802274 | 3.95E-09 | 3.88E-08 |
| ILMN_1714841 | OR4D9 | -1.5681137 | 2.45E-07 | 1.40E-06 |
| ILMN_1702807 | OR4E2 | -1.6378937 | 4.88E-11 | 9.22E-10 |
| ILMN_3236198 | OR4F17 | -2.6775555 | 1.69E-11 | 3.86E-10 |
| ILMN_2103646 | OR4F21 | -1.6272087 | 1.94E-10 | 2.90E-09 |
| ILMN_3242693 | OR4F29 | -1.7796364 | 4.29E-14 | 3.17E-12 |
| ILMN_1765445 | OR51Q1 | -2.4892745 | 2.23E-11 | 4.84E-10 |
| ILMN_1726969 | OR51S1 | -1.9130621 | 5.05E-09 | 4.80E-08 |
| ILMN_1811599 | OR52B6 | -2.810342 | 2.68E-11 | 5.60E-10 |
| ILMN_1743226 | OR52I1 | -2.2007735 | 3.98E-14 | 2.97E-12 |
| ILMN_1757795 | OR52K1 | -2.7394867 | 1.61E-14 | 1.46E-12 |
| ILMN_1762400 | OR52R1 | -2.6126916 | 1.03E-14 | 1.02E-12 |
| ILMN_1702824 | OR52W1 | -2.5121316 | 1.33E-11 | 3.18E-10 |
| ILMN_1705551 | OR56A4 | -2.4699954 | 3.70E-13 | 1.73E-11 |
| ILMN_1758663 | OR5AR1 | -1.8758194 | 1.09E-10 | 1.79E-09 |
| ILMN_2171733 | OR5AR1 | -1.6977617 | 8.14E-14 | 5.11E-12 |
| ILMN_1724677 | OR5B21 | -1.6917557 | 9.81E-08 | 6.24E-07 |
| ILMN_2070530 | OR5F1 | -1.6740962 | 9.22E-11 | 1.55E-09 |
| ILMN_1774715 | OR5P3 | -1.9684391 | 1.76E-11 | 3.98E-10 |
| ILMN_1691080 | OR6B1 | -1.5242574 | 3.26E-11 | 6.56E-10 |
| ILMN_1703910 | OR6C3 | -1.7653874 | 1.48E-09 | 1.64E-08 |
| ILMN_1815093 | OR6M1 | -2.6076493 | 8.81E-11 | 1.49E-09 |
| ILMN_2101270 | OR6S1 | -1.5649172 | 1.49E-11 | 3.48E-10 |
| ILMN_2199926 | OR7E37P | -1.5680256 | 1.70E-12 | 5.82E-11 |
| ILMN_2134723 | OR7E91P | -3.115054 | 6.40E-14 | 4.24E-12 |
| ILMN_1696584 | ORM1 | 1.8342296 | 3.47E-07 | 1.90E-06 |
| ILMN_1802151 | OSBPL5 | -1.6028928 | 8.43E-09 | 7.40E-08 |
| ILMN_2405078 | OSBPL8 | 1.5814483 | 2.60E-11 | 5.47E-10 |
| ILMN_1749114 | OTC | 1.6005464 | 3.80E-07 | 2.05E-06 |
| ILMN_2259155 | OTOL1 | -2.2253348 | 1.67E-08 | 1.34E-07 |
| ILMN_1715612 | OTOP2 | -1.8567662 | 1.17E-13 | 6.76E-12 |
| ILMN_1659270 | OTP | -2.8359963 | 4.56E-09 | 4.40E-08 |
| ILMN_1731851 | OXA1L | 2.0849511 | 5.20E-14 | 3.63E-12 |
| ILMN_2322446 | PABPC1L2B | -1.6220523 | 6.44E-11 | 1.15E-09 |
| ILMN_2280441 | PACRG | -2.2347961 | 1.26E-11 | 3.04E-10 |
| ILMN_3230157 | PACRGL | -2.2303213 | 3.81E-19 | 5.87E-16 |
| ILMN_3297013 | PACRGL | -1.5369694 | 8.07E-14 | 5.11E-12 |
| ILMN_1748093 | PAFAH1B3 | 1.6807402 | 7.47E-11 | 1.31E-09 |
| ILMN_1662214 | PAH | 2.0028509 | 1.49E-12 | 5.32E-11 |
| ILMN_2330382 | PAQR6 | -1.6500806 | 4.26E-07 | 2.27E-06 |
| ILMN_2262044 | PARP10 | 2.5309343 | 1.60E-12 | 5.54E-11 |
| ILMN_2397954 | PARP3 | 1.5196582 | 1.21E-08 | 1.01E-07 |
| ILMN_1797295 | PASD1 | -1.9646549 | 1.47E-15 | 2.24E-13 |
| ILMN_1759297 | PATZ1 | 2.2641318 | 3.77E-16 | 8.17E-14 |
| ILMN_1789653 | PBLD | 1.526861 | 8.84E-11 | 1.50E-09 |
| ILMN_2067303 | PBOV1 | -1.9371558 | 1.41E-14 | 1.31E-12 |
| ILMN_3239648 | PCA3 | -1.53592 | 3.98E-10 | 5.30E-09 |
| ILMN_1715458 | PCDH10 | -1.8881976 | 7.68E-15 | 8.15E-13 |
| ILMN_2185866 | PCDHB17 | -2.2131905 | 1.52E-10 | 2.36E-09 |
| ILMN_2047885 | PCDHB9 | -2.1028014 | 1.81E-15 | 2.66E-13 |
| ILMN_1671621 | PCMT1 | 2.2896972 | 1.61E-10 | 2.48E-09 |
| ILMN_1710070 | PCSK6 | 1.5177558 | 1.46E-12 | 5.22E-11 |
| ILMN_1676384 | PCSK6 | 2.0150097 | 7.35E-12 | 1.96E-10 |
| ILMN_1680339 | PDGFRL | -1.5914558 | 3.75E-08 | 2.70E-07 |
| ILMN_1771376 | PEA15 | 1.8548979 | 6.42E-11 | 1.15E-09 |
| ILMN_1728684 | PELP1 | 1.5169817 | 3.31E-08 | 2.42E-07 |
| ILMN_2151817 | PFN1 | 2.5340155 | 1.11E-14 | 1.09E-12 |
| ILMN_1717572 | PGA5 | -2.5716689 | 5.71E-10 | 7.25E-09 |
| ILMN_1733831 | PGBD5 | -2.4400352 | 4.27E-13 | 1.92E-11 |
| ILMN_3307921 | PGLYRP2 | 2.3103778 | 2.60E-08 | 1.96E-07 |
| ILMN_1684771 | PGRMC1 | 1.9800309 | 2.79E-14 | 2.24E-12 |
| ILMN_1666222 | PHACTR3 | -1.560767 | 1.28E-08 | 1.06E-07 |
| ILMN_2380101 | PHACTR4 | -2.1490216 | 8.60E-17 | 2.78E-14 |
| ILMN_1673461 | PHC2 | -1.6536955 | 4.77E-08 | 3.33E-07 |
| ILMN_3245476 | PHRF1 | 1.873959 | 1.41E-07 | 8.63E-07 |
| ILMN_2282352 | PHYH | 1.6487096 | 6.90E-13 | 2.76E-11 |
| ILMN_1773073 | PHYH | 1.7923365 | 1.12E-09 | 1.29E-08 |
| ILMN_1701413 | PIGQ | 2.2215044 | 4.63E-17 | 1.66E-14 |
| ILMN_1738263 | PIGU | 1.5226994 | 3.56E-09 | 3.55E-08 |
| ILMN_2178186 | PIGW | -1.9819384 | 4.86E-15 | 5.70E-13 |
| ILMN_1766275 | PIK3CD | -1.5049312 | 1.09E-09 | 1.26E-08 |
| ILMN_2110422 | PKD1L1 | -1.5566352 | 5.42E-12 | 1.51E-10 |
| ILMN_1716424 | PKP1 | -1.7453452 | 1.47E-13 | 8.02E-12 |
| ILMN_1808487 | PLA2G12B | 1.51281 | 2.88E-10 | 4.04E-09 |
| ILMN_2233050 | PLA2G2D | -1.686852 | 2.45E-13 | 1.23E-11 |
| ILMN_1710590 | PLA2G2E | -1.6340913 | 1.58E-11 | 3.64E-10 |
| ILMN_1697629 | PLA2G4B | 1.7179382 | 1.11E-12 | 4.13E-11 |
| ILMN_1657344 | PLAG1 | -1.6759572 | 7.65E-12 | 2.03E-10 |
| ILMN_1808379 | PLCD4 | -1.5756936 | 1.52E-07 | 9.17E-07 |
| ILMN_1666976 | PLD3 | 1.7347967 | 2.13E-12 | 6.96E-11 |
| ILMN_1659290 | PLEKHB2 | -1.7218565 | 7.11E-09 | 6.40E-08 |
| ILMN_1714446 | PLG | 1.9979302 | 1.28E-08 | 1.06E-07 |
| ILMN_2138765 | PLIN2 | 1.5004249 | 7.85E-09 | 6.97E-08 |
| ILMN_2041788 | PLS3 | 1.8908488 | 1.35E-07 | 8.31E-07 |
| ILMN_3245239 | PNMAL2 | -1.7069884 | 2.84E-10 | 3.99E-09 |
| ILMN_1710027 | PNMT | -1.9104612 | 6.56E-08 | 4.39E-07 |
| ILMN_2297511 | PODXL | -1.8653292 | 9.54E-12 | 2.43E-10 |
| ILMN_1677138 | POLR2J3 | 1.5575552 | 1.22E-12 | 4.51E-11 |
| ILMN_3249444 | POM121L4P | -2.1496312 | 1.47E-09 | 1.63E-08 |
| ILMN_2287707 | POT1 | -2.1660766 | 1.09E-17 | 5.72E-15 |
| ILMN_2370916 | POTE2 | -1.5391971 | 1.16E-10 | 1.88E-09 |
| ILMN_1738691 | POU4F1 | -2.9458968 | 1.63E-18 | 1.40E-15 |
| ILMN_1796969 | POU4F3 | -2.3669249 | 6.54E-09 | 5.95E-08 |
| ILMN_1773914 | POU6F2 | -2.4515451 | 6.17E-14 | 4.14E-12 |
| ILMN_2342455 | PPA2 | -1.5016659 | 3.80E-18 | 2.53E-15 |
| ILMN_1776094 | PPCS | 2.2523551 | 4.74E-13 | 2.07E-11 |
| ILMN_2382127 | PPFIA1 | 1.8252251 | 1.77E-12 | 6.00E-11 |
| ILMN_1803318 | PPFIA2 | -2.5305287 | 2.68E-13 | 1.31E-11 |
| ILMN_1667791 | PPFIA4 | -2.2370331 | 5.37E-11 | 9.98E-10 |
| ILMN_3237769 | PPIAL4G | -2.5504685 | 3.89E-12 | 1.14E-10 |
| ILMN_2285490 | PPIL3 | -1.5487967 | 4.11E-14 | 3.06E-12 |
| ILMN_1806867 | PPM1G | 1.8363448 | 1.78E-09 | 1.93E-08 |
| ILMN_2070043 | PPM1K | -1.719403 | 1.37E-13 | 7.63E-12 |
| ILMN_1761968 | PPP1R14A | 2.0411942 | 1.88E-08 | 1.49E-07 |
| ILMN_1757882 | PPP1R16A | 1.6938072 | 7.74E-09 | 6.89E-08 |
| ILMN_1722858 | PPP2CA | 2.212124 | 8.19E-13 | 3.18E-11 |
| ILMN_2298365 | PPP2R2B | -2.7331363 | 8.65E-17 | 2.78E-14 |
| ILMN_1669365 | PPP2R2B | -1.5225715 | 1.52E-08 | 1.24E-07 |
| ILMN_1729123 | PPP2R4 | 2.6249584 | 1.13E-13 | 6.55E-12 |
| ILMN_1789283 | PPP2R5C | 2.1614377 | 2.68E-16 | 6.38E-14 |
| ILMN_1798620 | PQLC1 | 1.7424868 | 3.85E-10 | 5.15E-09 |
| ILMN_1801832 | PRAC | -1.9328375 | 9.16E-15 | 9.42E-13 |
| ILMN_3248384 | PRAC | -1.5542977 | 8.02E-13 | 3.13E-11 |
| ILMN_1742068 | PRAMEF10 | -2.4715096 | 2.70E-10 | 3.82E-09 |
| ILMN_3241950 | PRAMEF18 | -2.4194682 | 2.62E-11 | 5.50E-10 |
| ILMN_2045270 | PRAMEF6 | -1.6138907 | 2.50E-10 | 3.59E-09 |
| ILMN_2104821 | PRAMEF7 | -3.1062406 | 1.90E-18 | 1.59E-15 |
| ILMN_1815556 | PRAP1 | 2.5536864 | 6.72E-11 | 1.20E-09 |
| ILMN_1769091 | PRCP | 1.7678625 | 9.66E-10 | 1.14E-08 |
| ILMN_1815120 | PRDM14 | -1.798018 | 7.65E-11 | 1.33E-09 |
| ILMN_1732023 | PRDM7 | -2.2151087 | 4.84E-12 | 1.37E-10 |
| ILMN_2366388 | PRDX1 | 1.8190203 | 3.60E-09 | 3.58E-08 |
| ILMN_2395969 | PRDX3 | 1.5733266 | 1.98E-12 | 6.58E-11 |
| ILMN_1741356 | PRICKLE1 | -1.7070389 | 1.06E-07 | 6.70E-07 |
| ILMN_2389590 | PRKAR1A | 1.5852652 | 3.41E-09 | 3.41E-08 |
| ILMN_1806908 | PRKCB1 | -1.6200546 | 1.63E-07 | 9.73E-07 |
| ILMN_1769517 | PRKDC | 1.5509438 | 3.54E-06 | 1.49E-05 |
| ILMN_1655711 | PRLHR | -1.5853603 | 1.24E-07 | 7.69E-07 |
| ILMN_1692473 | PRMT1 | 1.5735918 | 1.42E-16 | 4.04E-14 |
| ILMN_1687721 | PROC | 2.0377092 | 1.44E-11 | 3.37E-10 |
| ILMN_1704751 | PROKR1 | -1.564863 | 3.80E-07 | 2.05E-06 |
| ILMN_3245936 | PRR20B | -3.0628099 | 2.36E-12 | 7.62E-11 |
| ILMN_3246071 | PRR20C | -2.9860502 | 9.21E-10 | 1.09E-08 |
| ILMN_1734773 | PRSS1 | -2.639987 | 4.30E-10 | 5.67E-09 |
| ILMN_1749109 | PSAP | 2.1216136 | 4.93E-10 | 6.39E-09 |
| ILMN_2355559 | PSAP | 2.4319198 | 2.70E-10 | 3.82E-09 |
| ILMN_1771538 | PSCA | -2.2658658 | 2.28E-11 | 4.93E-10 |
| ILMN_2309615 | PSG6 | -2.0439406 | 8.14E-14 | 5.11E-12 |
| ILMN_1709611 | PSMA1 | 1.6133172 | 8.19E-11 | 1.40E-09 |
| ILMN_1759952 | PSMA5 | 1.7211683 | 2.35E-11 | 5.05E-10 |
| ILMN_1683026 | PSMB10 | 1.5051558 | 2.07E-07 | 1.21E-06 |
| ILMN_3235768 | PSMB11 | -1.5437386 | 5.91E-08 | 4.01E-07 |
| ILMN_2344130 | PSMD4 | 1.5180412 | 1.78E-13 | 9.54E-12 |
| ILMN_1728355 | PSMD4 | 1.6063794 | 1.55E-12 | 5.46E-11 |
| ILMN_2088410 | PSMG2 | 2.3235188 | 3.96E-12 | 1.16E-10 |
| ILMN_1799669 | PSORS1C1 | -1.9471652 | 3.34E-10 | 4.58E-09 |
| ILMN_1653447 | PSORS1C2 | -2.6256441 | 2.60E-11 | 5.49E-10 |
| ILMN_1655154 | PTBP1 | 1.9360145 | 1.64E-10 | 2.50E-09 |
| ILMN_2333319 | PTBP1 | 2.5106233 | 6.19E-14 | 4.14E-12 |
| ILMN_2345015 | PTGES2 | 2.0856088 | 9.87E-12 | 2.49E-10 |
| ILMN_1764508 | PTK6 | -2.1107095 | 1.87E-09 | 2.01E-08 |
| ILMN_1700109 | PTOV1 | 1.8506563 | 4.55E-14 | 3.31E-12 |
| ILMN_1725791 | PTPLA | -1.6745578 | 1.28E-10 | 2.06E-09 |
| ILMN_1690114 | PTPLAD2 | -1.6482724 | 9.37E-08 | 6.01E-07 |
| ILMN_2170353 | PTPLB | 1.5703859 | 5.58E-09 | 5.21E-08 |
| ILMN_1743175 | PTPRT | -1.6137907 | 3.57E-07 | 1.95E-06 |
| ILMN_1698885 | PTPRT | -1.6022616 | 2.44E-10 | 3.52E-09 |
| ILMN_1779404 | PUF60 | 1.5523827 | 2.44E-09 | 2.54E-08 |
| ILMN_2401155 | PUM1 | 1.7229872 | 4.46E-16 | 9.34E-14 |
| ILMN_1681634 | PXMP2 | 1.9075092 | 2.44E-06 | 1.07E-05 |
| ILMN_1700268 | QPRT | 2.591454 | 5.68E-11 | 1.04E-09 |
| ILMN_3241626 | QRFPR | -1.6724005 | 1.84E-13 | 9.70E-12 |
| ILMN_2291619 | RAB3IP | -2.5057117 | 2.04E-15 | 2.89E-13 |
| ILMN_1712705 | RAB40C | 1.7199997 | 1.59E-12 | 5.52E-11 |
| ILMN_1763765 | RAD1 | -2.307415 | 4.95E-10 | 6.41E-09 |
| ILMN_2363027 | RAD51 | -1.7864532 | 1.29E-10 | 2.08E-09 |
| ILMN_1781345 | RAD52 | -1.8285549 | 2.45E-12 | 7.83E-11 |
| ILMN_2164007 | RAET1E | -1.7618903 | 4.16E-10 | 5.50E-09 |
| ILMN_1659836 | RAET1G | -1.8030794 | 4.98E-12 | 1.40E-10 |
| ILMN_1733115 | RALGAPB | 1.6949617 | 3.89E-15 | 4.80E-13 |
| ILMN_1662198 | RANGAP1 | 1.5990802 | 1.61E-14 | 1.46E-12 |
| ILMN_1776519 | RAP1GAP | 1.6426858 | 1.03E-08 | 8.77E-08 |
| ILMN_1691787 | RAPH1 | 2.8150522 | 4.29E-19 | 6.13E-16 |
| ILMN_1659206 | RARA | 2.038694 | 8.46E-10 | 1.02E-08 |
| ILMN_1810844 | RARRES2 | 1.6905288 | 1.39E-11 | 3.28E-10 |
| ILMN_1733110 | RASSF7 | 1.994324 | 1.06E-16 | 3.19E-14 |
| ILMN_3239653 | RAX2 | -2.3409632 | 2.27E-20 | 1.05E-16 |
| ILMN_1653412 | RAXL1 | -2.0781016 | 2.43E-12 | 7.78E-11 |
| ILMN_1782745 | RBL1 | -1.7511068 | 2.13E-11 | 4.66E-10 |
| ILMN_2414014 | RBM10 | 2.3281368 | 7.91E-16 | 1.44E-13 |
| ILMN_1666739 | RBM15 | 1.5414032 | 1.07E-04 | 3.20E-04 |
| ILMN_2411963 | RBM39 | 2.3499033 | 6.29E-13 | 2.60E-11 |
| ILMN_1745130 | RBM9 | 2.2674244 | 2.29E-12 | 7.43E-11 |
| ILMN_2386732 | RCHY1 | -1.725045 | 9.13E-16 | 1.56E-13 |
| ILMN_1676795 | REG3G | -1.6088518 | 5.90E-14 | 4.01E-12 |
| ILMN_1706301 | RET | -1.8923779 | 6.98E-11 | 1.23E-09 |
| ILMN_3307777 | RETNLB | -1.5231372 | 2.71E-08 | 2.03E-07 |
| ILMN_1753008 | REXO1 | 2.1075222 | 1.98E-16 | 4.96E-14 |
| ILMN_1810639 | REXO1L1 | -3.9177113 | 2.09E-13 | 1.08E-11 |
| ILMN_3242842 | REXO1L2P | -4.0941414 | 2.17E-13 | 1.12E-11 |
| ILMN_2396287 | RFX2 | -1.7333893 | 1.26E-07 | 7.79E-07 |
| ILMN_2116827 | RGPD1 | -1.6670923 | 3.21E-14 | 2.50E-12 |
| ILMN_1711288 | RGS20 | -1.6236441 | 6.47E-08 | 4.34E-07 |
| ILMN_1802205 | RHOB | 1.6580626 | 9.81E-11 | 1.64E-09 |
| ILMN_1756880 | RIBC1 | -1.5645916 | 7.11E-15 | 7.65E-13 |
| ILMN_1700759 | RIMS1 | -2.0316616 | 4.25E-09 | 4.14E-08 |
| ILMN_3242405 | RMRP | 2.618981 | 1.71E-11 | 3.89E-10 |
| ILMN_1739423 | RN7SK | 1.7198685 | 4.60E-09 | 4.43E-08 |
| ILMN_1712849 | RNASE7 | -1.6217929 | 1.77E-07 | 1.05E-06 |
| ILMN_2356031 | RNF121 | 1.603964 | 5.23E-15 | 5.94E-13 |
| ILMN_1737698 | RNF126P1 | -2.2732974 | 6.46E-13 | 2.64E-11 |
| ILMN_1767446 | RNF150 | -1.7117658 | 6.03E-08 | 4.09E-07 |
| ILMN_1792389 | RNF165 | -2.497533 | 7.82E-11 | 1.35E-09 |
| ILMN_3225534 | RNF216L | -2.0826112 | 4.01E-11 | 7.78E-10 |
| ILMN_1711862 | RNF7 | 1.6238346 | 5.74E-09 | 5.33E-08 |
| ILMN_3240155 | RNU105A | 1.7418665 | 1.26E-08 | 1.05E-07 |
| ILMN_3309453 | RNU4-1 | 2.6590614 | 1.82E-08 | 1.45E-07 |
| ILMN_3308138 | RNU4-2 | 2.348465 | 2.79E-10 | 3.94E-09 |
| ILMN_3240594 | RNU4ATAC | 1.8123672 | 3.21E-11 | 6.47E-10 |
| ILMN_3237617 | RNU5A | 2.4677485 | 7.15E-09 | 6.44E-08 |
| ILMN_3246433 | RNY5 | 1.9184819 | 9.70E-07 | 4.71E-06 |
| ILMN_1672446 | RPL11 | 1.5480714 | 7.29E-09 | 6.53E-08 |
| ILMN_2116366 | RPL12 | 1.7124035 | 2.79E-10 | 3.94E-09 |
| ILMN_2230624 | RPL18 | 2.8168637 | 7.88E-16 | 1.44E-13 |
| ILMN_1701832 | RPL19 | 1.9685996 | 8.03E-14 | 5.11E-12 |
| ILMN_1755115 | RPL23 | 1.6977236 | 1.96E-13 | 1.02E-11 |
| ILMN_1731546 | RPL26 | 2.1105399 | 3.89E-11 | 7.60E-10 |
| ILMN_2319994 | RPL3 | 1.6414359 | 6.46E-15 | 7.12E-13 |
| ILMN_1754195 | RPL31 | 1.6911948 | 3.01E-08 | 2.23E-07 |
| ILMN_2142815 | RPL35 | 1.5354447 | 2.17E-09 | 2.30E-08 |
| ILMN_2189936 | RPL36AL | 1.7608571 | 7.67E-10 | 9.36E-09 |
| ILMN_1765043 | RPL38 | 2.3519662 | 9.12E-10 | 1.08E-08 |
| ILMN_2343775 | RPL38 | 2.8340817 | 1.86E-14 | 1.64E-12 |
| ILMN_2087080 | RPL5 | 2.2303406 | 1.88E-11 | 4.19E-10 |
| ILMN_2408415 | RPL9 | 1.6236562 | 9.90E-07 | 4.79E-06 |
| ILMN_1755733 | RPLP2 | 2.5660762 | 1.07E-10 | 1.76E-09 |
| ILMN_1693421 | RPN2 | 2.1284579 | 3.01E-11 | 6.15E-10 |
| ILMN_1777906 | RPRC1 | 1.8167803 | 9.35E-09 | 8.09E-08 |
| ILMN_1740587 | RPS11 | 1.5596573 | 7.37E-13 | 2.91E-11 |
| ILMN_2338785 | RPS14 | 1.5784228 | 2.78E-09 | 2.87E-08 |
| ILMN_1651850 | RPS16 | 2.4132056 | 1.26E-11 | 3.04E-10 |
| ILMN_1784717 | RPS19 | 2.2703648 | 4.67E-13 | 2.05E-11 |
| ILMN_1746516 | RPS25 | 2.9665041 | 1.97E-11 | 4.37E-10 |
| ILMN_2126802 | RPS27L | -2.6350988 | 2.10E-20 | 1.05E-16 |
| ILMN_1738243 | RPS29 | 2.9214589 | 4.09E-11 | 7.91E-10 |
| ILMN_1808939 | RPS6 | 2.319661 | 1.19E-14 | 1.15E-12 |
| ILMN_2364357 | RPS6KB2 | 2.4539777 | 8.90E-16 | 1.54E-13 |
| ILMN_1730082 | RPUSD4 | 1.5696669 | 6.64E-14 | 4.37E-12 |
| ILMN_2360784 | RRBP1 | 2.3537793 | 2.10E-15 | 2.96E-13 |
| ILMN_1803810 | RRBP1 | 2.8747456 | 1.81E-14 | 1.61E-12 |
| ILMN_1688178 | RRP7A | 2.0657446 | 4.53E-18 | 2.76E-15 |
| ILMN_1730611 | RTN4 | 1.6769833 | 1.19E-08 | 9.95E-08 |
| ILMN_1678627 | RUFY4 | -1.7041698 | 4.80E-07 | 2.53E-06 |
| ILMN_3230683 | RUNDC2B | -1.5476547 | 2.18E-09 | 2.31E-08 |
| ILMN_1669066 | RUNX2 | -1.6182423 | 1.21E-04 | 3.58E-04 |
| ILMN_1774074 | RXRB | 1.7898719 | 6.30E-16 | 1.20E-13 |
| ILMN_1728262 | SAA2 | 1.6650065 | 3.59E-06 | 1.51E-05 |
| ILMN_2287168 | SAR1B | -1.8549429 | 2.91E-13 | 1.40E-11 |
| ILMN_3246489 | SBK2 | -1.9288617 | 5.25E-08 | 3.63E-07 |
| ILMN_2402499 | SC4MOL | -1.7392236 | 6.87E-13 | 2.76E-11 |
| ILMN_3235325 | SCARNA13 | 2.3086085 | 7.62E-13 | 3.00E-11 |
| ILMN_3245822 | SCARNA17 | 2.4047481 | 1.83E-13 | 9.67E-12 |
| ILMN_3246869 | SCARNA21 | 1.511123 | 3.95E-09 | 3.88E-08 |
| ILMN_3240069 | SCARNA4 | 1.7061658 | 6.42E-13 | 2.63E-11 |
| ILMN_3236408 | SCARNA5 | 1.8523366 | 1.53E-12 | 5.42E-11 |
| ILMN_3247159 | SCARNA8 | 2.1929998 | 3.27E-10 | 4.49E-09 |
| ILMN_2303912 | SCD5 | -1.7981327 | 2.50E-08 | 1.90E-07 |
| ILMN_1655347 | SCGB1A1 | -2.6879165 | 6.22E-11 | 1.12E-09 |
| ILMN_1754757 | SCNN1D | 2.1880704 | 1.96E-15 | 2.81E-13 |
| ILMN_1726516 | SCRIB | 1.8982275 | 2.36E-08 | 1.81E-07 |
| ILMN_1741617 | SCRT1 | -1.573983 | 1.16E-06 | 5.49E-06 |
| ILMN_1753523 | SDC3 | 1.8639072 | 4.07E-11 | 7.88E-10 |
| ILMN_1749213 | SDF2L1 | 2.1518692 | 4.07E-10 | 5.40E-09 |
| ILMN_2148679 | SDHAP3 | -1.9146642 | 2.19E-12 | 7.11E-11 |
| ILMN_1676709 | SDK2 | -2.1356086 | 4.74E-09 | 4.54E-08 |
| ILMN_1811114 | SDS | 1.8266771 | 2.99E-07 | 1.67E-06 |
| ILMN_3249074 | SEC1 | -1.5712792 | 1.53E-11 | 3.56E-10 |
| ILMN_1659564 | SEC61A1 | 2.0000228 | 4.87E-12 | 1.37E-10 |
| ILMN_1651429 | SELM | 1.8038205 | 6.93E-08 | 4.61E-07 |
| ILMN_1655374 | SELV | -2.0701387 | 2.95E-11 | 6.06E-10 |
| ILMN_2289887 | SEPN1 | -2.1625049 | 2.13E-12 | 6.97E-11 |
| ILMN_2204826 | SEPP1 | 1.9576965 | 2.85E-08 | 2.13E-07 |
| ILMN_1769118 | Septin 9 | 1.9562322 | 2.41E-14 | 2.00E-12 |
| ILMN_1789136 | SERF2 | 1.7636962 | 9.26E-14 | 5.69E-12 |
| ILMN_2338452 | SERPINA1 | 2.1994205 | 7.08E-10 | 8.73E-09 |
| ILMN_2404154 | SERPINA1 | 2.899752 | 5.72E-14 | 3.92E-12 |
| ILMN_1788874 | SERPINA3 | 2.7020829 | 7.72E-13 | 3.03E-11 |
| ILMN_1705243 | SERPINA7 | 1.536821 | 7.85E-09 | 6.97E-08 |
| ILMN_1712400 | SERPINB6 | 2.7533993 | 8.06E-17 | 2.68E-14 |
| ILMN_2184250 | SERPINB9 | -1.6218155 | 9.86E-11 | 1.65E-09 |
| ILMN_1762605 | SERPINC1 | 2.3749946 | 2.98E-11 | 6.10E-10 |
| ILMN_1707975 | SERPIND1 | 1.5176445 | 9.83E-09 | 8.44E-08 |
| ILMN_2141482 | SERPINF1 | 2.5889378 | 1.49E-12 | 5.29E-11 |
| ILMN_1670305 | SERPING1 | 1.753139 | 4.51E-13 | 2.00E-11 |
| ILMN_1684320 | SEZ6L | -2.2406835 | 1.78E-08 | 1.42E-07 |
| ILMN_1705151 | SF3A3 | 2.0537867 | 8.92E-11 | 1.51E-09 |
| ILMN_2378868 | SFRS5 | 1.5085143 | 7.76E-10 | 9.43E-09 |
| ILMN_1727017 | SFTPB | -1.8071949 | 9.55E-17 | 2.94E-14 |
| ILMN_3245175 | SH2D7 | -1.7756693 | 4.33E-13 | 1.94E-11 |
| ILMN_1760990 | SH3GL3 | -1.712089 | 3.18E-06 | 1.36E-05 |
| ILMN_2182482 | SHCBP1 | -1.7814654 | 6.50E-13 | 2.65E-11 |
| ILMN_1661264 | SHMT2 | 1.7684839 | 2.04E-12 | 6.74E-11 |
| ILMN_1655549 | SIGLEC10 | -2.485763 | 5.26E-14 | 3.66E-12 |
| ILMN_1678729 | SIL1 | 2.1916563 | 3.56E-12 | 1.07E-10 |
| ILMN_1673886 | SIM2 | -1.6320432 | 1.28E-14 | 1.22E-12 |
| ILMN_1682930 | SIPA1 | 2.0391051 | 4.79E-14 | 3.44E-12 |
| ILMN_3250659 | SKA2 | -2.0855258 | 2.04E-15 | 2.89E-13 |
| ILMN_2409720 | SLA2 | -2.3976623 | 3.65E-17 | 1.39E-14 |
| ILMN_1792744 | SLC13A2 | -1.639056 | 4.53E-07 | 2.40E-06 |
| ILMN_1676192 | SLC13A5 | 2.2200334 | 2.93E-07 | 1.64E-06 |
| ILMN_1732410 | SLC16A9 | -1.6567302 | 1.81E-05 | 6.44E-05 |
| ILMN_2390017 | SLC22A6 | -1.6341094 | 1.36E-07 | 8.34E-07 |
| ILMN_1711826 | SLC24A2 | -1.9388122 | 3.08E-11 | 6.27E-10 |
| ILMN_1743911 | SLC25A39 | 2.2818638 | 6.24E-11 | 1.12E-09 |
| ILMN_2222880 | SLC25A42 | 1.6756039 | 2.48E-08 | 1.89E-07 |
| ILMN_1785252 | SLC26A6 | 1.6008254 | 7.23E-12 | 1.93E-10 |
| ILMN_1672575 | SLC26A8 | -1.7046876 | 5.68E-10 | 7.22E-09 |
| ILMN_1755720 | SLC2A2 | 1.846449 | 2.48E-09 | 2.59E-08 |
| ILMN_2151168 | SLC30A6 | -1.7145934 | 2.30E-14 | 1.95E-12 |
| ILMN_1794959 | SLC35F3 | -3.0352151 | 1.42E-17 | 6.82E-15 |
| ILMN_1793182 | SLC36A2 | -2.9115773 | 1.40E-12 | 5.06E-11 |
| ILMN_1678678 | SLC37A4 | 1.5474211 | 1.01E-11 | 2.53E-10 |
| ILMN_1651799 | SLC38A2 | 2.035366 | 5.84E-09 | 5.41E-08 |
| ILMN_1764629 | SLC39A14 | 1.6333149 | 3.40E-10 | 4.64E-09 |
| ILMN_2234970 | SLC39A3 | 2.0939448 | 7.23E-14 | 4.70E-12 |
| ILMN_1808152 | SLC41A3 | -1.7845146 | 2.53E-09 | 2.63E-08 |
| ILMN_2330307 | SLC43A3 | 1.5819178 | 1.16E-11 | 2.84E-10 |
| ILMN_1771987 | SLC44A2 | 1.597449 | 1.98E-11 | 4.37E-10 |
| ILMN_2274775 | SLC44A4 | -1.8027011 | 2.89E-11 | 5.94E-10 |
| ILMN_1670325 | SLC47A1 | 1.9033053 | 6.28E-09 | 5.76E-08 |
| ILMN_2273224 | SLC4A5 | -2.0996305 | 1.10E-12 | 4.11E-11 |
| ILMN_1738300 | SLC4A8 | -1.7449903 | 1.18E-13 | 6.78E-12 |
| ILMN_1811221 | SLC5A8 | -1.8615123 | 7.83E-15 | 8.28E-13 |
| ILMN_1798577 | SLC6A11 | -1.6817137 | 3.20E-05 | 1.08E-04 |
| ILMN_1698846 | SLC8A2 | -2.5819932 | 3.24E-12 | 9.92E-11 |
| ILMN_2087656 | SLCO2B1 | 1.5041117 | 1.39E-11 | 3.28E-10 |
| ILMN_2215862 | SLFN13 | -1.8701568 | 9.36E-15 | 9.58E-13 |
| ILMN_1791702 | SMARCA2 | 1.8090786 | 1.79E-11 | 4.03E-10 |
| ILMN_3240236 | SMCR5 | -2.6979801 | 2.51E-20 | 1.05E-16 |
| ILMN_2093389 | SNAPC1 | -2.0129193 | 1.98E-15 | 2.83E-13 |
| ILMN_3236955 | SNORA11 | 1.6411596 | 2.18E-08 | 1.69E-07 |
| ILMN_3246465 | SNORA16A | 1.7676529 | 3.58E-11 | 7.08E-10 |
| ILMN_3243129 | SNORA17 | 2.4965893 | 5.08E-15 | 5.83E-13 |
| ILMN_3244348 | SNORA18 | 1.9860287 | 3.52E-11 | 6.99E-10 |
| ILMN_3245228 | SNORA20 | 2.3510581 | 4.54E-12 | 1.30E-10 |
| ILMN_3246713 | SNORA22 | 1.6129273 | 1.82E-13 | 9.64E-12 |
| ILMN_3247939 | SNORA23 | 2.4641468 | 4.39E-15 | 5.26E-13 |
| ILMN_3248890 | SNORA24 | 2.5819499 | 3.80E-11 | 7.44E-10 |
| ILMN_1682038 | SNORA25 | 1.5322935 | 2.33E-11 | 5.02E-10 |
| ILMN_3238613 | SNORA26 | 1.5903043 | 1.34E-10 | 2.14E-09 |
| ILMN_3239108 | SNORA3 | 2.1940663 | 7.89E-10 | 9.55E-09 |
| ILMN_2085525 | SNORA32 | 1.8445228 | 1.40E-12 | 5.05E-11 |
| ILMN_3240231 | SNORA34 | 1.9220064 | 1.70E-06 | 7.77E-06 |
| ILMN_3247494 | SNORA36B | 2.120439 | 1.96E-11 | 4.34E-10 |
| ILMN_3235270 | SNORA38B | 1.8917139 | 8.41E-13 | 3.25E-11 |
| ILMN_3245764 | SNORA39 | 1.9294413 | 3.02E-09 | 3.07E-08 |
| ILMN_3241798 | SNORA41 | 1.7930935 | 1.29E-08 | 1.07E-07 |
| ILMN_3243908 | SNORA43 | 2.1928129 | 5.50E-07 | 2.85E-06 |
| ILMN_3238078 | SNORA45 | 2.8690948 | 4.10E-13 | 1.85E-11 |
| ILMN_3238670 | SNORA47 | 2.1270799 | 2.70E-08 | 2.03E-07 |
| ILMN_3235231 | SNORA49 | 2.3454707 | 5.07E-11 | 9.52E-10 |
| ILMN_3242825 | SNORA50 | 1.852881 | 1.09E-09 | 1.26E-08 |
| ILMN_3235316 | SNORA51 | 2.470716 | 2.36E-14 | 1.97E-12 |
| ILMN_3237035 | SNORA54 | 2.8085193 | 5.79E-15 | 6.45E-13 |
| ILMN_3238745 | SNORA55 | 2.0131789 | 1.75E-11 | 3.95E-10 |
| ILMN_3235404 | SNORA57 | 2.1571366 | 5.76E-09 | 5.35E-08 |
| ILMN_3236942 | SNORA5A | 1.5960006 | 1.14E-09 | 1.31E-08 |
| ILMN_3245458 | SNORA61 | 1.9702767 | 4.59E-10 | 5.99E-09 |
| ILMN_3249167 | SNORA63 | 1.5706749 | 3.99E-11 | 7.77E-10 |
| ILMN_1787326 | SNORA65 | 3.1150828 | 8.34E-13 | 3.23E-11 |
| ILMN_1689616 | SNORA66 | 2.1097162 | 1.97E-10 | 2.93E-09 |
| ILMN_3240418 | SNORA72 | 1.6818036 | 7.76E-10 | 9.43E-09 |
| ILMN_3240022 | SNORA73A | 1.7867859 | 8.07E-10 | 9.76E-09 |
| ILMN_3243677 | SNORA73B | 2.1642611 | 1.25E-11 | 3.02E-10 |
| ILMN_3236224 | SNORA74A | 2.2800267 | 1.44E-09 | 1.60E-08 |
| ILMN_3240150 | SNORA75 | 2.3272644 | 4.36E-13 | 1.95E-11 |
| ILMN_3243428 | SNORA77 | 1.8198803 | 1.13E-10 | 1.85E-09 |
| ILMN_3238707 | SNORA8 | 1.8153429 | 1.61E-10 | 2.48E-09 |
| ILMN_3235969 | SNORA9 | 1.9563769 | 1.20E-12 | 4.44E-11 |
| ILMN_3238955 | SNORD10 | 2.0143388 | 8.09E-12 | 2.12E-10 |
| ILMN_1673181 | SNORD100 | 1.7476688 | 6.98E-09 | 6.30E-08 |
| ILMN_3235397 | SNORD110 | 2.2609583 | 2.61E-14 | 2.12E-12 |
| ILMN_3242908 | SNORD117 | 1.9582259 | 3.69E-13 | 1.72E-11 |
| ILMN_3236049 | SNORD12 | 2.6036925 | 3.94E-13 | 1.79E-11 |
| ILMN_3240002 | SNORD12B | 1.7245946 | 1.41E-14 | 1.31E-12 |
| ILMN_1713832 | SNORD15B | 2.1052472 | 1.22E-09 | 1.39E-08 |
| ILMN_1669210 | SNORD16 | 2.4048273 | 2.90E-13 | 1.39E-11 |
| ILMN_1774973 | SNORD21 | 2.1352709 | 6.36E-15 | 7.06E-13 |
| ILMN_1784328 | SNORD25 | 1.765092 | 1.76E-12 | 6.00E-11 |
| ILMN_2072391 | SNORD31 | 1.9174112 | 8.96E-09 | 7.79E-08 |
| ILMN_1682354 | SNORD33 | 3.0189651 | 7.37E-14 | 4.77E-12 |
| ILMN_2165762 | SNORD34 | 1.9087843 | 5.14E-14 | 3.60E-12 |
| ILMN_1667609 | SNORD35A | 1.8999886 | 3.62E-07 | 1.97E-06 |
| ILMN_1694367 | SNORD35B | 1.7480265 | 4.23E-06 | 1.75E-05 |
| ILMN_2135175 | SNORD36A | 1.8593995 | 1.11E-09 | 1.28E-08 |
| ILMN_2160160 | SNORD36C | 1.9041207 | 8.41E-12 | 2.19E-10 |
| ILMN_1684278 | SNORD38A | 2.1814838 | 3.39E-11 | 6.77E-10 |
| ILMN_3239574 | SNORD3A | 2.1993139 | 2.08E-08 | 1.62E-07 |
| ILMN_3242315 | SNORD3D | 1.649483 | 2.54E-06 | 1.11E-05 |
| ILMN_2157020 | SNORD48 | 3.1422484 | 1.91E-16 | 4.87E-14 |
| ILMN_3248562 | SNORD49A | 1.892595 | 5.73E-12 | 1.58E-10 |
| ILMN_3246391 | SNORD52 | 1.5715563 | 1.62E-11 | 3.72E-10 |
| ILMN_2209515 | SNORD56 | 2.1122832 | 2.10E-10 | 3.10E-09 |
| ILMN_3241139 | SNORD57 | 1.6940351 | 2.27E-09 | 2.40E-08 |
| ILMN_3238481 | SNORD60 | 1.7557297 | 5.88E-07 | 3.03E-06 |
| ILMN_3248270 | SNORD62B | 1.7451618 | 1.79E-08 | 1.42E-07 |
| ILMN_3246353 | SNORD65 | 1.5226388 | 2.77E-06 | 1.20E-05 |
| ILMN_3247671 | SNORD66 | 2.5386166 | 9.44E-12 | 2.41E-10 |
| ILMN_3248712 | SNORD67 | 1.9550591 | 3.71E-12 | 1.10E-10 |
| ILMN_2082762 | SNORD68 | 1.8070723 | 9.60E-12 | 2.43E-10 |
| ILMN_3239272 | SNORD71 | 3.1476323 | 1.43E-13 | 7.85E-12 |
| ILMN_3248874 | SNORD8 | 2.0679726 | 1.11E-11 | 2.75E-10 |
| ILMN_3244157 | SNORD83B | 1.5705252 | 2.99E-05 | 1.02E-04 |
| ILMN_3245672 | SNORD84 | 1.9501467 | 2.81E-09 | 2.89E-08 |
| ILMN_3247303 | SNORD85 | 2.399707 | 1.92E-11 | 4.26E-10 |
| ILMN_3238662 | SNORD89 | 2.3648673 | 1.57E-11 | 3.63E-10 |
| ILMN_3242443 | SNORD91B | 1.9452663 | 1.10E-08 | 9.30E-08 |
| ILMN_3246529 | SNORD93 | 1.6949446 | 1.38E-08 | 1.14E-07 |
| ILMN_3243452 | SNORD95 | 2.5151415 | 1.43E-10 | 2.25E-09 |
| ILMN_3244640 | SNORD96A | 1.6910924 | 7.00E-12 | 1.87E-10 |
| ILMN_3242144 | SNORD97 | 1.990347 | 3.27E-09 | 3.30E-08 |
| ILMN_3237516 | SNRNP48 | -1.7425257 | 1.33E-17 | 6.61E-15 |
| ILMN_1732053 | SNRNP70 | 2.5352098 | 3.92E-18 | 2.55E-15 |
| ILMN_1774661 | SNRPB | 1.8666605 | 1.51E-10 | 2.35E-09 |
| ILMN_2369785 | SNRPD2 | 2.6383329 | 3.33E-12 | 1.01E-10 |
| ILMN_1727975 | SNTG1 | -1.5212744 | 4.09E-08 | 2.91E-07 |
| ILMN_2404320 | SNTN | -2.2631995 | 1.76E-17 | 7.91E-15 |
| ILMN_1683737 | SNX32 | -1.8875821 | 6.97E-11 | 1.23E-09 |
| ILMN_1662438 | SOD1 | 1.8985133 | 1.64E-11 | 3.76E-10 |
| ILMN_2336781 | SOD2 | 1.8660871 | 5.95E-13 | 2.47E-11 |
| ILMN_2406501 | SOD2 | 2.1548936 | 6.65E-13 | 2.70E-11 |
| ILMN_1792922 | SOD2 | 2.8521459 | 8.81E-16 | 1.54E-13 |
| ILMN_1703427 | SON | 1.5462392 | 4.90E-16 | 9.83E-14 |
| ILMN_2258689 | SORBS1 | -2.1862245 | 4.95E-19 | 6.59E-16 |
| ILMN_1680251 | SORCS1 | -2.5372332 | 5.29E-15 | 5.98E-13 |
| ILMN_1773459 | SOX11 | -2.2646422 | 5.88E-10 | 7.42E-09 |
| ILMN_1671092 | SOX17 | -1.9453007 | 1.96E-11 | 4.33E-10 |
| ILMN_1793213 | SOX30 | -2.099639 | 1.08E-07 | 6.80E-07 |
| ILMN_2181306 | SPACA5B | -2.1597121 | 1.14E-11 | 2.81E-10 |
| ILMN_2118201 | SPAG11A | -3.0074124 | 1.85E-15 | 2.70E-13 |
| ILMN_1800838 | SPATA12 | -1.5425739 | 2.49E-15 | 3.36E-13 |
| ILMN_1687247 | SPATA20 | 1.89022 | 5.43E-12 | 1.51E-10 |
| ILMN_2149400 | SPC25 | -2.0179673 | 1.32E-13 | 7.41E-12 |
| ILMN_1665280 | SPCS1 | 1.5159306 | 2.69E-07 | 1.52E-06 |
| ILMN_2329171 | SPDYE1 | -2.4727918 | 4.84E-18 | 2.90E-15 |
| ILMN_2381476 | SPG3A | -1.820278 | 6.85E-10 | 8.50E-09 |
| ILMN_1754529 | SPG7 | 1.5027687 | 7.08E-08 | 4.69E-07 |
| ILMN_1690017 | SPINK5 | -1.5001667 | 1.39E-08 | 1.14E-07 |
| ILMN_2211018 | SPRR2E | -2.0500466 | 1.06E-10 | 1.75E-09 |
| ILMN_1810835 | SPRR3 | -1.5417081 | 4.49E-11 | 8.56E-10 |
| ILMN_1662618 | SQSTM1 | 1.6418945 | 8.15E-09 | 7.19E-08 |
| ILMN_1734602 | SRRM2 | 2.3055333 | 1.65E-17 | 7.53E-15 |
| ILMN_1811820 | SRRM4 | -1.9143913 | 3.36E-15 | 4.28E-13 |
| ILMN_1796407 | SS18L2 | 1.9198833 | 9.32E-11 | 1.57E-09 |
| ILMN_2292723 | SSX2 | -1.5188029 | 6.21E-12 | 1.69E-10 |
| ILMN_3301818 | ST20 | -1.8614148 | 6.38E-16 | 1.20E-13 |
| ILMN_1756501 | ST6GAL1 | 2.0701378 | 9.57E-12 | 2.43E-10 |
| ILMN_1735584 | ST8SIA5 | -2.5449415 | 4.94E-15 | 5.76E-13 |
| ILMN_1677497 | STAB2 | -1.6648985 | 3.75E-10 | 5.03E-09 |
| ILMN_2362832 | STAG3L1 | -2.0496717 | 1.48E-10 | 2.31E-09 |
| ILMN_2281529 | STAP2 | -1.6308798 | 2.07E-12 | 6.83E-11 |
| ILMN_1777325 | STAT1 | 1.6616239 | 1.01E-06 | 4.88E-06 |
| ILMN_1690105 | STAT1 | 1.8871278 | 6.23E-14 | 4.14E-12 |
| ILMN_1690921 | STAT2 | 1.8570181 | 2.27E-09 | 2.39E-08 |
| ILMN_2281089 | STEAP3 | -2.1944794 | 1.18E-15 | 1.90E-13 |
| ILMN_1737313 | STK31 | -2.1476196 | 4.56E-07 | 2.41E-06 |
| ILMN_1693538 | STK36 | 1.5581032 | 5.14E-11 | 9.62E-10 |
| ILMN_1769634 | STRA13 | 1.6616642 | 4.77E-11 | 9.03E-10 |
| ILMN_1749345 | STX5 | 1.5652949 | 2.75E-06 | 1.19E-05 |
| ILMN_1779616 | SUCLG1 | 2.267855 | 1.41E-10 | 2.22E-09 |
| ILMN_2333594 | SUMO2 | 1.8825243 | 5.40E-09 | 5.06E-08 |
| ILMN_1693270 | SUSD2 | 1.5702914 | 1.43E-08 | 1.17E-07 |
| ILMN_2089175 | SYAP1 | -1.633447 | 8.86E-15 | 9.16E-13 |
| ILMN_1756374 | SYCP1 | -1.5261722 | 7.91E-17 | 2.66E-14 |
| ILMN_1783837 | SYN1 | -1.6185054 | 1.31E-12 | 4.77E-11 |
| ILMN_1690253 | SYNPO2L | -1.7644747 | 2.45E-15 | 3.33E-13 |
| ILMN_1690545 | TAF11 | -1.6089515 | 2.32E-12 | 7.48E-11 |
| ILMN_2061327 | TAF13 | 1.8351332 | 4.64E-08 | 3.25E-07 |
| ILMN_2061318 | TAF13 | 1.8520599 | 2.05E-08 | 1.60E-07 |
| ILMN_1727281 | TAF6L | 1.7562289 | 3.79E-10 | 5.08E-09 |
| ILMN_1691892 | TAGLN2 | 1.5984226 | 2.98E-12 | 9.25E-11 |
| ILMN_1791678 | TAT | 2.0203011 | 2.37E-08 | 1.82E-07 |
| ILMN_1793118 | TAX1BP1 | 1.5922419 | 1.28E-13 | 7.23E-12 |
| ILMN_1726239 | TBCA | 1.9775252 | 1.24E-11 | 3.00E-10 |
| ILMN_2357770 | TCEA1 | 1.5984416 | 1.71E-11 | 3.89E-10 |
| ILMN_3251393 | TCEAL6 | -1.6503067 | 1.53E-11 | 3.55E-10 |
| ILMN_1774705 | TCEB3B | -2.2434072 | 5.03E-11 | 9.45E-10 |
| ILMN_1810590 | TCEB3C | -1.5005699 | 1.59E-06 | 7.31E-06 |
| ILMN_3237069 | TCEB3CL | -2.1017415 | 1.00E-13 | 6.03E-12 |
| ILMN_2175094 | TDRD1 | -1.5880044 | 4.48E-15 | 5.35E-13 |
| ILMN_3241282 | TDRD12 | -1.8590573 | 2.67E-14 | 2.15E-12 |
| ILMN_2357377 | TERF1 | -2.6001342 | 4.40E-19 | 6.13E-16 |
| ILMN_1685490 | TEX14 | -1.6879746 | 2.30E-09 | 2.42E-08 |
| ILMN_1768425 | TF | 1.7517964 | 9.66E-08 | 6.16E-07 |
| ILMN_2275760 | TFAP2A | -1.9691321 | 7.31E-09 | 6.55E-08 |
| ILMN_1659378 | TFAP2D | -1.6714544 | 3.39E-14 | 2.60E-12 |
| ILMN_1722489 | TFF1 | -1.6051794 | 1.15E-09 | 1.32E-08 |
| ILMN_1695000 | TFIP11 | -1.959592 | 1.92E-10 | 2.87E-09 |
| ILMN_1662619 | TFPI | 1.5576457 | 2.43E-14 | 2.00E-12 |
| ILMN_1663866 | TGFBI | 1.5982109 | 9.66E-15 | 9.66E-13 |
| ILMN_2356578 | TH | -1.7841465 | 6.40E-09 | 5.85E-08 |
| ILMN_1677273 | TH | -1.6804589 | 2.95E-09 | 3.01E-08 |
| ILMN_2358560 | TIAM2 | -1.638161 | 7.53E-08 | 4.95E-07 |
| ILMN_1711566 | TIMP1 | 2.0634934 | 4.36E-10 | 5.73E-09 |
| ILMN_1749078 | TIMP2 | 1.6282238 | 1.29E-10 | 2.07E-09 |
| ILMN_1663399 | TIMP4 | -1.9653437 | 3.83E-12 | 1.13E-10 |
| ILMN_1743763 | TJAP1 | 1.5991191 | 2.91E-08 | 2.17E-07 |
| ILMN_1736597 | TKT | 1.8031382 | 3.42E-12 | 1.03E-10 |
| ILMN_1696643 | TLN1 | 1.5957911 | 1.38E-10 | 2.19E-09 |
| ILMN_1770338 | TM4SF1 | 1.7561469 | 1.75E-09 | 1.91E-08 |
| ILMN_1792404 | TM4SF4 | 2.7448509 | 1.34E-11 | 3.19E-10 |
| ILMN_2167808 | TM4SF5 | 2.0631877 | 6.85E-09 | 6.21E-08 |
| ILMN_1785765 | TM9SF2 | 1.9053093 | 2.84E-14 | 2.27E-12 |
| ILMN_1693311 | TMBIM6 | 1.5733255 | 3.08E-11 | 6.26E-10 |
| ILMN_1793829 | TMCO1 | 1.812503 | 3.72E-13 | 1.73E-11 |
| ILMN_1693045 | TMED1 | 1.7249889 | 1.28E-09 | 1.45E-08 |
| ILMN_1654939 | TMED2 | 1.5129622 | 4.79E-09 | 4.59E-08 |
| ILMN_1730645 | TMEFF2 | -1.6648886 | 9.79E-09 | 8.41E-08 |
| ILMN_3249244 | TMEM106A | -1.7325487 | 8.30E-11 | 1.42E-09 |
| ILMN_1712035 | TMEM115 | 2.3014053 | 1.32E-12 | 4.81E-11 |
| ILMN_1724139 | TMEM123 | 1.6376773 | 3.75E-12 | 1.11E-10 |
| ILMN_1721563 | TMEM127 | 1.870981 | 1.19E-11 | 2.91E-10 |
| ILMN_1772514 | TMEM132E | -1.7186739 | 2.17E-09 | 2.30E-08 |
| ILMN_1774066 | TMEM141 | 1.6115008 | 2.48E-10 | 3.57E-09 |
| ILMN_3243788 | TMEM146 | -1.8107435 | 5.89E-11 | 1.07E-09 |
| ILMN_2095660 | TMEM156 | -1.7075584 | 1.05E-09 | 1.22E-08 |
| ILMN_2085012 | TMEM176B | 1.5141262 | 4.35E-05 | 1.43E-04 |
| ILMN_1730734 | TMEM205 | 3.1303803 | 1.22E-14 | 1.17E-12 |
| ILMN_1749516 | TMEM225 | -1.5514147 | 1.06E-10 | 1.76E-09 |
| ILMN_1692754 | TMEM49 | 1.8595026 | 4.71E-14 | 3.41E-12 |
| ILMN_1731742 | TNFRSF13C | -2.2311566 | 5.03E-12 | 1.41E-10 |
| ILMN_1697409 | TNFRSF14 | 2.1423068 | 1.54E-10 | 2.39E-09 |
| ILMN_2349633 | TNFRSF18 | -1.8289531 | 1.74E-06 | 7.92E-06 |
| ILMN_1770711 | TNFRSF8 | -1.9398939 | 1.40E-10 | 2.21E-09 |
| ILMN_1707591 | TNIP3 | -2.2664023 | 8.62E-10 | 1.03E-08 |
| ILMN_1753789 | TNN | -1.9396831 | 3.30E-09 | 3.33E-08 |
| ILMN_1806492 | TNR | -1.9116718 | 3.49E-20 | 1.14E-16 |
| ILMN_1739573 | TNRC6A | 1.5220231 | 5.31E-14 | 3.68E-12 |
| ILMN_1672004 | TOB1 | 1.5553117 | 4.59E-10 | 5.99E-09 |
| ILMN_2082314 | TOM1 | 2.4062303 | 6.70E-13 | 2.71E-11 |
| ILMN_1679796 | TOMM20 | 1.8811593 | 2.82E-12 | 8.83E-11 |
| ILMN_2087060 | TOMM7 | 2.5049295 | 5.03E-07 | 2.64E-06 |
| ILMN_1716687 | TPM1 | 1.5543382 | 7.58E-08 | 4.98E-07 |
| ILMN_2360710 | TPM1 | 1.6744285 | 8.41E-09 | 7.39E-08 |
| ILMN_1727479 | TPRG1L | 1.8179282 | 1.32E-09 | 1.48E-08 |
| ILMN_1769219 | TPSG1 | -2.2559746 | 5.55E-09 | 5.18E-08 |
| ILMN_2094718 | TPT1 | 2.3309816 | 7.92E-12 | 2.09E-10 |
| ILMN_1755737 | TRABD | 2.3508574 | 5.00E-15 | 5.79E-13 |
| ILMN_1780397 | TRAF3IP3 | -1.6961271 | 1.34E-08 | 1.11E-07 |
| ILMN_1737146 | TRAM1 | 1.661612 | 9.40E-11 | 1.58E-09 |
| ILMN_1751627 | TRAPPC3 | 1.5508032 | 5.02E-05 | 1.62E-04 |
| ILMN_1690783 | TREML1 | -1.6454458 | 1.25E-07 | 7.76E-07 |
| ILMN_2262275 | TRIM13 | -1.5047356 | 3.51E-12 | 1.06E-10 |
| ILMN_2193443 | TRIM16L | -2.2920319 | 2.67E-18 | 1.99E-15 |
| ILMN_2255142 | TRIM34 | -1.7185741 | 2.99E-12 | 9.26E-11 |
| ILMN_2241775 | TROVE2 | 1.9283838 | 2.16E-09 | 2.29E-08 |
| ILMN_1679401 | TRPM4 | 1.7382533 | 5.72E-10 | 7.26E-09 |
| ILMN_1714216 | TSC2 | 2.2707525 | 4.08E-17 | 1.53E-14 |
| ILMN_3246534 | TSG1 | -1.7669141 | 7.94E-13 | 3.10E-11 |
| ILMN_1777881 | TSPAN17 | 1.9556389 | 7.60E-10 | 9.28E-09 |
| ILMN_1749403 | TSPAN33 | 1.780942 | 4.20E-11 | 8.10E-10 |
| ILMN_1718607 | TSPAN4 | 2.4138253 | 6.63E-15 | 7.27E-13 |
| ILMN_1729453 | TSPAN9 | 1.7328318 | 2.50E-08 | 1.90E-07 |
| ILMN_1681679 | TSPO | 2.0902658 | 5.04E-11 | 9.45E-10 |
| ILMN_2063254 | TSPY2 | -1.7401191 | 6.63E-06 | 2.62E-05 |
| ILMN_1691572 | TST | 1.6216057 | 2.64E-08 | 1.99E-07 |
| ILMN_1715332 | TTC21A | -1.5672874 | 9.21E-10 | 1.09E-08 |
| ILMN_2217955 | TTC21B | -2.3524689 | 1.22E-21 | 1.82E-17 |
| ILMN_1799329 | TTLL10 | -1.5445312 | 3.36E-11 | 6.71E-10 |
| ILMN_2198912 | TTR | 2.9370669 | 2.45E-12 | 7.85E-11 |
| ILMN_1680874 | TUBB2B | -1.5859516 | 4.01E-07 | 2.15E-06 |
| ILMN_1657893 | TXNRD2 | 1.5848569 | 3.44E-11 | 6.86E-10 |
| ILMN_1676955 | TYK2 | 1.8859215 | 2.46E-13 | 1.24E-11 |
| ILMN_1768930 | U2AF2 | 1.6289445 | 2.36E-10 | 3.43E-09 |
| ILMN_1782977 | UBA52 | 3.01424 | 1.89E-13 | 9.84E-12 |
| ILMN_1794612 | UBA7 | 2.1080756 | 3.63E-12 | 1.08E-10 |
| ILMN_1678841 | UBD | 2.1468001 | 1.29E-09 | 1.45E-08 |
| ILMN_2320853 | UBE2D3 | 1.7587883 | 3.56E-09 | 3.55E-08 |
| ILMN_1806778 | UBE2E1 | 1.6782089 | 1.94E-08 | 1.53E-07 |
| ILMN_1769520 | UBE2L6 | 1.5914917 | 2.40E-12 | 7.73E-11 |
| ILMN_1711470 | UBE2T | -1.6762981 | 8.64E-09 | 7.56E-08 |
| ILMN_1806394 | UBL4B | -2.4384821 | 1.62E-17 | 7.53E-15 |
| ILMN_1748907 | UBQLNL | -1.9610052 | 2.29E-10 | 3.34E-09 |
| ILMN_3248990 | UBTFL2 | -1.5130233 | 5.48E-09 | 5.13E-08 |
| ILMN_3239254 | UCA1 | -3.037371 | 8.52E-14 | 5.31E-12 |
| ILMN_2048414 | UGT2B15 | 1.779311 | 2.43E-09 | 2.54E-08 |
| ILMN_1655565 | UGT3A2 | -2.1924036 | 7.25E-09 | 6.51E-08 |
| ILMN_3238676 | ULBP2 | -1.7600261 | 9.56E-09 | 8.25E-08 |
| ILMN_2288254 | UNC45A | -1.7728149 | 1.87E-08 | 1.48E-07 |
| ILMN_2099301 | UNC84B | 2.1749195 | 1.77E-14 | 1.59E-12 |
| ILMN_1746652 | UNC93A | -1.5498285 | 2.75E-07 | 1.55E-06 |
| ILMN_2383693 | UPF2 | 1.7549748 | 2.03E-10 | 3.02E-09 |
| ILMN_1671191 | UQCRC1 | 2.1358993 | 7.82E-12 | 2.07E-10 |
| ILMN_1797384 | UROS | 1.5555147 | 8.96E-12 | 2.31E-10 |
| ILMN_3238990 | USP17 | -2.9250365 | 1.19E-13 | 6.82E-12 |
| ILMN_1680279 | USP49 | -1.7378256 | 1.04E-13 | 6.19E-12 |
| ILMN_2102580 | UTP20 | -1.9723958 | 7.37E-14 | 4.77E-12 |
| ILMN_2307903 | VCAM1 | 1.6379961 | 3.98E-07 | 2.14E-06 |
| ILMN_2278413 | VCX2 | -1.7033232 | 1.23E-12 | 4.54E-11 |
| ILMN_3223807 | VENTXP1 | -2.0229951 | 2.01E-10 | 2.99E-09 |
| ILMN_2203753 | VENTXP7 | -2.1551332 | 1.24E-13 | 7.05E-12 |
| ILMN_2058251 | VIM | 1.6548611 | 3.63E-11 | 7.15E-10 |
| ILMN_2111255 | VIT | -1.6479866 | 4.80E-12 | 1.36E-10 |
| ILMN_1691127 | VTN | 1.8605497 | 6.81E-11 | 1.21E-09 |
| ILMN_3309759 | VTRNA1-1 | 1.9559918 | 2.69E-08 | 2.02E-07 |
| ILMN_3310441 | VTRNA1-2 | 1.5294888 | 3.63E-06 | 1.53E-05 |
| ILMN_3236561 | VWA5B1 | -1.8534931 | 7.49E-10 | 9.16E-09 |
| ILMN_1752755 | VWF | 2.4711925 | 8.95E-12 | 2.31E-10 |
| ILMN_1788604 | WBP2 | 2.0107705 | 1.13E-11 | 2.80E-10 |
| ILMN_2367440 | WDR17 | -2.131008 | 1.40E-16 | 4.03E-14 |
| ILMN_1694479 | WDR18 | 2.4555563 | 2.50E-12 | 7.98E-11 |
| ILMN_1809866 | WDR74 | 1.9045247 | 2.81E-16 | 6.54E-14 |
| ILMN_2120429 | WFDC10A | -1.679097 | 1.66E-16 | 4.46E-14 |
| ILMN_1804328 | WWP1 | 2.3498508 | 8.63E-16 | 1.53E-13 |
| ILMN_1742618 | XAF1 | 1.6196024 | 5.48E-13 | 2.33E-11 |
| ILMN_2258977 | XAGE1 | -1.6655572 | 2.53E-11 | 5.38E-10 |
| ILMN_1773238 | XCR1 | -2.5535916 | 1.65E-19 | 3.11E-16 |
| ILMN_1705865 | XIRP2 | -2.4389743 | 8.52E-14 | 5.31E-12 |
| ILMN_1728512 | YWHAH | 1.5817358 | 1.18E-07 | 7.40E-07 |
| ILMN_1801101 | ZBTB48 | 2.3249544 | 5.26E-14 | 3.66E-12 |
| ILMN_1702389 | ZC3H3 | 2.3157681 | 1.67E-12 | 5.72E-11 |
| ILMN_1714402 | ZCCHC2 | 1.7078282 | 1.40E-16 | 4.03E-14 |
| ILMN_1795228 | ZFAND5 | 1.8943618 | 8.36E-09 | 7.35E-08 |
| ILMN_1759219 | ZMAT5 | -2.2317537 | 1.78E-17 | 7.91E-15 |
| ILMN_2403823 | ZMYM2 | 1.5984825 | 8.20E-12 | 2.15E-10 |
| ILMN_2184869 | ZMYM6 | -2.0570041 | 3.25E-15 | 4.19E-13 |
| ILMN_2127936 | ZNF100 | -1.6421284 | 7.37E-10 | 9.03E-09 |
| ILMN_2222786 | ZNF192 | -1.9382303 | 4.75E-14 | 3.44E-12 |
| ILMN_1670895 | ZNF207 | 1.5142298 | 7.60E-07 | 3.80E-06 |
| ILMN_1815578 | ZNF223 | -1.9330963 | 9.87E-11 | 1.65E-09 |
| ILMN_2288740 | ZNF266 | -2.4597509 | 9.48E-20 | 2.73E-16 |
| ILMN_2346562 | ZNF273 | -2.667615 | 1.22E-20 | 1.05E-16 |
| ILMN_1710268 | ZNF385D | -1.5224187 | 6.09E-07 | 3.13E-06 |
| ILMN_2158164 | ZNF430 | -1.8378863 | 8.52E-18 | 4.71E-15 |
| ILMN_1786079 | ZNF471 | -1.8310342 | 1.25E-09 | 1.42E-08 |
| ILMN_3240605 | ZNF488 | -2.4144315 | 1.46E-15 | 2.24E-13 |
| ILMN_1815118 | ZNF554 | -2.0644397 | 2.33E-14 | 1.97E-12 |
| ILMN_2084489 | ZNF595 | -2.1590324 | 5.77E-13 | 2.43E-11 |
| ILMN_1684032 | ZNF613 | -1.509415 | 8.05E-15 | 8.48E-13 |
| ILMN_2290732 | ZNF626 | -1.8908397 | 5.12E-14 | 3.60E-12 |
| ILMN_3237839 | ZNF674 | -1.7814975 | 1.01E-11 | 2.53E-10 |
| ILMN_1747943 | ZNF695 | -1.8149511 | 2.42E-13 | 1.22E-11 |
| ILMN_1700583 | ZNF750 | -2.5272105 | 2.26E-15 | 3.13E-13 |
| ILMN_2078404 | ZNF765 | -2.0963644 | 5.23E-15 | 5.94E-13 |
| ILMN_1708632 | ZNF771 | -2.1089368 | 4.97E-16 | 9.89E-14 |
| ILMN_1713706 | ZNF786 | -1.6550117 | 1.60E-19 | 3.11E-16 |
| ILMN_1655644 | ZNF804A | -1.8236346 | 3.01E-11 | 6.15E-10 |
| ILMN_1694080 | ZNF804B | -2.369752 | 8.36E-12 | 2.18E-10 |
| ILMN_2175737 | ZNF826 | -1.7956873 | 7.23E-18 | 4.15E-15 |
| ILMN_1727574 | ZNF827 | -2.2206366 | 7.39E-10 | 9.05E-09 |
| ILMN_1658016 | ZNF831 | -1.767199 | 2.56E-14 | 2.09E-12 |
| ILMN_1804268 | ZNF835 | -2.1053054 | 5.88E-13 | 2.45E-11 |
| ILMN_1744398 | ZSCAN10 | -2.0990452 | 5.27E-13 | 2.25E-11 |
| ILMN_1684960 | ZSWIM3 | -1.5271649 | 2.06E-08 | 1.61E-07 |

Abbreviation: HCC, hepatocellular carcinoma

Table S2. Functions of sorafenib-response targets, as determined by performing GO analysis.

| ID | Description | P value | FDR |
| --- | --- | --- | --- |
| GO:0072378 | blood coagulation, fibrin clot formation | 4.43E-09 | 1.03E-05 |
| GO:0034367 | protein-containing complex remodeling | 7.75E-09 | 1.03E-05 |
| GO:0034368 | protein-lipid complex remodeling | 7.75E-09 | 1.03E-05 |
| GO:0034369 | plasma lipoprotein particle remodeling | 7.75E-09 | 1.03E-05 |
| GO:0006413 | translational initiation | 1.59E-08 | 1.69E-05 |
| GO:0034375 | high-density lipoprotein particle remodeling | 2.28E-08 | 2.02E-05 |
| GO:0002526 | acute inflammatory response | 2.04E-07 | 0.000154467 |
| GO:0006614 | SRP-dependent cotranslational protein targeting to membrane | 2.58E-07 | 0.000170998 |
| GO:0002576 | platelet degranulation | 3.22E-07 | 0.000189677 |
| GO:0006613 | cotranslational protein targeting to membrane | 4.46E-07 | 0.000236457 |
| GO:0071827 | plasma lipoprotein particle organization | 6.07E-07 | 0.000270657 |
| GO:0045047 | protein targeting to ER | 6.34E-07 | 0.000270657 |
| GO:0016054 | organic acid catabolic process | 7.14E-07 | 0.000270657 |
| GO:0046395 | carboxylic acid catabolic process | 7.14E-07 | 0.000270657 |
| GO:0000184 | nuclear-transcribed mRNA catabolic process, nonsense-mediated decay | 8.83E-07 | 0.000312348 |
| GO:0072599 | establishment of protein localization to endoplasmic reticulum | 1.24E-06 | 0.000412439 |
| GO:0016999 | antibiotic metabolic process | 1.57E-06 | 0.000466124 |
| GO:0033700 | phospholipid efflux | 1.58E-06 | 0.000466124 |
| GO:0071825 | protein-lipid complex subunit organization | 1.97E-06 | 0.000551251 |
| GO:0034377 | plasma lipoprotein particle assembly | 3.56E-06 | 0.000944923 |
| GO:0070972 | protein localization to endoplasmic reticulum | 4.01E-06 | 0.001014018 |
| GO:0072594 | establishment of protein localization to organelle | 6.70E-06 | 0.001615396 |
| GO:0034370 | triglyceride-rich lipoprotein particle remodeling | 7.29E-06 | 0.001682399 |
| GO:0044282 | small molecule catabolic process | 1.02E-05 | 0.002251106 |
| GO:0034378 | chylomicron assembly | 1.51E-05 | 0.002942676 |
| GO:0009991 | response to extracellular stimulus | 1.51E-05 | 0.002942676 |
| GO:0065005 | protein-lipid complex assembly | 1.57E-05 | 0.002942676 |
| GO:0043691 | reverse cholesterol transport | 1.58E-05 | 0.002942676 |
| GO:0009636 | response to toxic substance | 1.61E-05 | 0.002942676 |
| GO:0006402 | mRNA catabolic process | 1.91E-05 | 0.003370395 |
| GO:0006631 | fatty acid metabolic process | 2.05E-05 | 0.003510496 |
| GO:0072376 | protein activation cascade | 2.45E-05 | 0.004058007 |
| GO:0006605 | protein targeting | 2.66E-05 | 0.004272114 |
| GO:0019080 | viral gene expression | 3.09E-05 | 0.004753849 |
| GO:0034372 | very-low-density lipoprotein particle remodeling | 3.14E-05 | 0.004753849 |
| GO:0042737 | drug catabolic process | 3.46E-05 | 0.00501314 |
| GO:0006401 | RNA catabolic process | 3.50E-05 | 0.00501314 |
| GO:0034433 | steroid esterification | 4.06E-05 | 0.005389218 |
| GO:0034434 | sterol esterification | 4.06E-05 | 0.005389218 |
| GO:0034435 | cholesterol esterification | 4.06E-05 | 0.005389218 |
| GO:0006953 | acute-phase response | 4.65E-05 | 0.006016347 |
| GO:0006612 | protein targeting to membrane | 5.06E-05 | 0.00638636 |
| GO:1903035 | negative regulation of response to wounding | 5.78E-05 | 0.006861193 |
| GO:0006069 | ethanol oxidation | 5.92E-05 | 0.006861193 |
| GO:0010872 | regulation of cholesterol esterification | 5.92E-05 | 0.006861193 |
| GO:0043062 | extracellular structure organization | 5.95E-05 | 0.006861193 |
| GO:0031667 | response to nutrient levels | 6.28E-05 | 0.007089935 |
| GO:0030195 | negative regulation of blood coagulation | 7.25E-05 | 0.008007865 |
| GO:1900047 | negative regulation of hemostasis | 8.96E-05 | 0.009696671 |
| GO:0007597 | blood coagulation, intrinsic pathway | 0.000100318 | 0.010567526 |
| GO:0031424 | keratinization | 0.000101592 | 0.010567526 |
| GO:0034374 | low-density lipoprotein particle remodeling | 0.000103603 | 0.010569531 |
| GO:0007584 | response to nutrient | 0.000112364 | 0.011247019 |
| GO:0002437 | inflammatory response to antigenic stimulus | 0.000117909 | 0.011372885 |
| GO:0048483 | autonomic nervous system development | 0.000117909 | 0.011372885 |
| GO:0002700 | regulation of production of molecular mediator of immune response | 0.000130844 | 0.012395177 |
| GO:0019083 | viral transcription | 0.000135718 | 0.012631263 |
| GO:0097006 | regulation of plasma lipoprotein particle levels | 0.000141412 | 0.012934289 |
| GO:0001825 | blastocyst formation | 0.000151038 | 0.013580643 |
| GO:0070268 | cornification | 0.000160535 | 0.014193951 |
| GO:0050819 | negative regulation of coagulation | 0.000163222 | 0.014195002 |
| GO:0050911 | detection of chemical stimulus involved in sensory perception of smell | 0.000171101 | 0.014640201 |
| GO:0071276 | cellular response to cadmium ion | 0.000185605 | 0.015629103 |
| GO:0007608 | sensory perception of smell | 0.000192983 | 0.015996492 |
| GO:0015711 | organic anion transport | 0.000202277 | 0.016508922 |
| GO:0006959 | humoral immune response | 0.000219363 | 0.017632097 |
| GO:0009063 | cellular amino acid catabolic process | 0.000229046 | 0.01813565 |
| GO:0034447 | very-low-density lipoprotein particle clearance | 0.00025768 | 0.020102854 |
| GO:0015914 | phospholipid transport | 0.000281032 | 0.021015745 |
| GO:0019915 | lipid storage | 0.000281032 | 0.021015745 |
| GO:0033344 | cholesterol efflux | 0.000283391 | 0.021015745 |
| GO:0045907 | positive regulation of vasoconstriction | 0.000285228 | 0.021015745 |
| GO:0010038 | response to metal ion | 0.000292397 | 0.021248876 |
| GO:0061045 | negative regulation of wound healing | 0.000312197 | 0.022381154 |
| GO:0000956 | nuclear-transcribed mRNA catabolic process | 0.00032453 | 0.02295511 |
| GO:0046686 | response to cadmium ion | 0.000337304 | 0.023411499 |
| GO:0072175 | epithelial tube formation | 0.000341271 | 0.023411499 |
| GO:0009593 | detection of chemical stimulus | 0.000344222 | 0.023411499 |
| GO:0030193 | regulation of blood coagulation | 0.000361602 | 0.024282277 |
| GO:0002718 | regulation of cytokine production involved in immune response | 0.000379599 | 0.024941678 |
| GO:0050907 | detection of chemical stimulus involved in sensory perception | 0.000380825 | 0.024941678 |
| GO:0002739 | regulation of cytokine secretion involved in immune response | 0.000405738 | 0.025933036 |
| GO:0060192 | negative regulation of lipase activity | 0.000405738 | 0.025933036 |
| GO:1900046 | regulation of hemostasis | 0.000417538 | 0.026369533 |
| GO:0097305 | response to alcohol | 0.000430604 | 0.026874764 |
| GO:0055090 | acylglycerol homeostasis | 0.000488293 | 0.029774665 |
| GO:0070328 | triglyceride homeostasis | 0.000488293 | 0.029774665 |
| GO:0015748 | organophosphate ester transport | 0.000495285 | 0.0298578 |
| GO:0046394 | carboxylic acid biosynthetic process | 0.000557336 | 0.032997846 |
| GO:0009913 | epidermal cell differentiation | 0.000563071 | 0.032997846 |
| GO:0002720 | positive regulation of cytokine production involved in immune response | 0.000566033 | 0.032997846 |
| GO:0051004 | regulation of lipoprotein lipase activity | 0.000574048 | 0.033101383 |
| GO:0016053 | organic acid biosynthetic process | 0.000586638 | 0.033385098 |
| GO:0034384 | high-density lipoprotein particle clearance | 0.000591555 | 0.033385098 |
| GO:0043648 | dicarboxylic acid metabolic process | 0.000638408 | 0.035650034 |
| GO:0072329 | monocarboxylic acid catabolic process | 0.000648153 | 0.035817198 |
| GO:0016125 | sterol metabolic process | 0.000694299 | 0.037736231 |
| GO:0015850 | organic hydroxy compound transport | 0.000697107 | 0.037736231 |
| GO:0050818 | regulation of coagulation | 0.000721031 | 0.038344274 |
| GO:0034380 | high-density lipoprotein particle assembly | 0.000722795 | 0.038344274 |
| GO:0006656 | phosphatidylcholine biosynthetic process | 0.000748768 | 0.039204125 |
| GO:0015918 | sterol transport | 0.000761173 | 0.039204125 |
| GO:1990748 | cellular detoxification | 0.000761173 | 0.039204125 |
| GO:0030100 | regulation of endocytosis | 0.000770294 | 0.039292393 |
| GO:0030301 | cholesterol transport | 0.000794578 | 0.040145104 |
| GO:0042026 | protein refolding | 0.000837173 | 0.041898155 |
| GO:0098869 | cellular oxidant detoxification | 0.000889807 | 0.044116149 |
| GO:0044242 | cellular lipid catabolic process | 0.000898642 | 0.044141626 |
| GO:0030216 | keratinocyte differentiation | 0.00091408 | 0.044488007 |
| GO:0090150 | establishment of protein localization to membrane | 0.000923872 | 0.044555805 |
| GO:0008202 | steroid metabolic process | 0.000939908 | 0.044920827 |
| GO:0019216 | regulation of lipid metabolic process | 0.000969439 | 0.045918534 |
| GO:0043312 | neutrophil degranulation | 0.000995105 | 0.046123735 |
| GO:0042730 | fibrinolysis | 0.000999855 | 0.046123735 |
| GO:1900120 | regulation of receptor binding | 0.000999855 | 0.046123735 |
| GO:0016042 | lipid catabolic process | 0.001029288 | 0.047072188 |
| GO:1901654 | response to ketone | 0.00104701 | 0.047473412 |
| GO:0043434 | response to peptide hormone | 0.001071871 | 0.047884272 |
| GO:0001838 | embryonic epithelial tube formation | 0.001079844 | 0.047884272 |
| GO:0006898 | receptor-mediated endocytosis | 0.001091521 | 0.047884272 |
| GO:0042119 | neutrophil activation | 0.001092177 | 0.047884272 |
| GO:0002283 | neutrophil activation involved in immune response | 0.001139679 | 0.049557371 |
| GO:0072562 | blood microparticle | 3.53E-12 | 2.16E-09 |
| GO:0060205 | cytoplasmic vesicle lumen | 3.63E-10 | 8.29E-08 |
| GO:0031983 | vesicle lumen | 4.07E-10 | 8.29E-08 |
| GO:0034774 | secretory granule lumen | 3.68E-09 | 5.62E-07 |
| GO:0022626 | cytosolic ribosome | 3.49E-08 | 4.26E-06 |
| GO:0005925 | focal adhesion | 1.18E-07 | 1.21E-05 |
| GO:0005924 | cell-substrate adherens junction | 1.40E-07 | 1.22E-05 |
| GO:0031093 | platelet alpha granule lumen | 1.96E-07 | 1.44E-05 |
| GO:0030055 | cell-substrate junction | 2.12E-07 | 1.44E-05 |
| GO:0031091 | platelet alpha granule | 1.41E-06 | 8.63E-05 |
| GO:0062023 | collagen-containing extracellular matrix | 1.73E-06 | 9.61E-05 |
| GO:0044391 | ribosomal subunit | 2.83E-06 | 0.000136353 |
| GO:0034364 | high-density lipoprotein particle | 2.90E-06 | 0.000136353 |
| GO:0022625 | cytosolic large ribosomal subunit | 4.32E-06 | 0.000188429 |
| GO:0005912 | adherens junction | 6.84E-06 | 0.000278613 |
| GO:0031012 | extracellular matrix | 1.97E-05 | 0.000710553 |
| GO:0034361 | very-low-density lipoprotein particle | 2.09E-05 | 0.000710553 |
| GO:0034385 | triglyceride-rich plasma lipoprotein particle | 2.09E-05 | 0.000710553 |
| GO:0044445 | cytosolic part | 3.67E-05 | 0.001178588 |
| GO:0042627 | chylomicron | 5.20E-05 | 0.001587553 |
| GO:0005788 | endoplasmic reticulum lumen | 5.46E-05 | 0.001587553 |
| GO:0034358 | plasma lipoprotein particle | 9.79E-05 | 0.002600815 |
| GO:1990777 | lipoprotein particle | 9.79E-05 | 0.002600815 |
| GO:0042611 | MHC protein complex | 0.000133733 | 0.003359203 |
| GO:0005775 | vacuolar lumen | 0.000137447 | 0.003359203 |
| GO:0032994 | protein-lipid complex | 0.000158431 | 0.003723138 |
| GO:0071682 | endocytic vesicle lumen | 0.000188201 | 0.004258925 |
| GO:0120111 | neuron projection cytoplasm | 0.000195433 | 0.00426462 |
| GO:0015934 | large ribosomal subunit | 0.000224716 | 0.004734526 |
| GO:0005840 | ribosome | 0.000262686 | 0.005350047 |
| GO:0045095 | keratin filament | 0.000553604 | 0.010911362 |
| GO:0022627 | cytosolic small ribosomal subunit | 0.000778052 | 0.014855936 |
| GO:0043202 | lysosomal lumen | 0.001157079 | 0.021423489 |
| GO:0034362 | low-density lipoprotein particle | 0.001472374 | 0.026459432 |
| GO:0048786 | presynaptic active zone | 0.001636966 | 0.027977547 |
| GO:0030134 | COPII-coated ER to Golgi transport vesicle | 0.001648432 | 0.027977547 |
| GO:0005759 | mitochondrial matrix | 0.001769777 | 0.029225241 |
| GO:0035580 | specific granule lumen | 0.002153342 | 0.034623477 |
| GO:0035578 | azurophil granule lumen | 0.00237548 | 0.037147232 |
| GO:1904115 | axon cytoplasm | 0.002431897 | 0.037147232 |
| GO:0012507 | ER to Golgi transport vesicle membrane | 0.002800762 | 0.04012889 |
| GO:1904724 | tertiary granule lumen | 0.002800762 | 0.04012889 |
| GO:0097197 | tetraspanin-enriched microdomain | 0.002824128 | 0.04012889 |
| GO:0004857 | enzyme inhibitor activity | 1.24E-06 | 0.001023835 |
| GO:0050839 | cell adhesion molecule binding | 8.61E-06 | 0.003555154 |
| GO:0016209 | antioxidant activity | 1.54E-05 | 0.004243613 |
| GO:0055102 | lipase inhibitor activity | 2.09E-05 | 0.004318586 |
| GO:0023026 | MHC class II protein complex binding | 3.51E-05 | 0.005793588 |
| GO:0061134 | peptidase regulator activity | 5.84E-05 | 0.008039416 |
| GO:0023023 | MHC protein complex binding | 0.000133749 | 0.01578235 |
| GO:0048037 | cofactor binding | 0.000182436 | 0.018836532 |
| GO:0030414 | peptidase inhibitor activity | 0.00024174 | 0.02107616 |
| GO:0051087 | chaperone binding | 0.000262985 | 0.02107616 |
| GO:0004866 | endopeptidase inhibitor activity | 0.000292099 | 0.02107616 |
| GO:0015485 | cholesterol binding | 0.000306191 | 0.02107616 |
| GO:0061135 | endopeptidase regulator activity | 0.000466806 | 0.029660156 |
| GO:0045296 | cadherin binding | 0.000572254 | 0.030859083 |
| GO:0003735 | structural constituent of ribosome | 0.000610545 | 0.030859083 |
| GO:0043177 | organic acid binding | 0.000623789 | 0.030859083 |
| GO:0008188 | neuropeptide receptor activity | 0.000635114 | 0.030859083 |
| GO:0032934 | sterol binding | 0.000798763 | 0.036654354 |
| GO:0005496 | steroid binding | 0.000957585 | 0.038475076 |
| GO:0072341 | modified amino acid binding | 0.000958988 | 0.038475076 |
| GO:0016769 | transferase activity, transferring nitrogenous groups | 0.00102476 | 0.038475076 |
| GO:0017127 | cholesterol transporter activity | 0.00102476 | 0.038475076 |
| GO:0031406 | carboxylic acid binding | 0.001232506 | 0.044263039 |

Abbreviation: GO, Gene Ontology

Table S3. Differentially expressed genes in the tissues of patients with HCC.

| Gene | logFC | P value | FDR |
| --- | --- | --- | --- |
| AKR1C2 | 1.624406096 | 5.01E-06 | 7.61E-06 |
| BRIP1 | 2.14420469 | 7.25E-17 | 2.69E-16 |
| FBLN1 | 2.273494891 | 1.30E-05 | 1.91E-05 |
| RPN2 | 1.13289279 | 3.35E-26 | 7.52E-25 |
| PSCA | 4.961837879 | 1.77E-12 | 4.39E-12 |
| MGAT5B | 2.743088548 | 5.48E-05 | 7.79E-05 |
| BNIPL | 2.384066618 | 1.75E-21 | 1.14E-20 |
| RAET1G | 1.339637503 | 3.14E-07 | 5.28E-07 |
| GSDMB | 1.436087638 | 1.66E-13 | 4.68E-13 |
| RBL1 | 2.007746968 | 2.87E-20 | 1.59E-19 |
| HSPA4 | 1.230240905 | 4.84E-26 | 1.03E-24 |
| FCAR | -1.33201377 | 3.34E-11 | 7.69E-11 |
| CAPN11 | 2.671335093 | 2.86E-26 | 6.58E-25 |
| LRRN4CL | 2.912747284 | 0.038987702 | 0.044391938 |
| FAM50A | 1.696804068 | 9.82E-24 | 1.01E-22 |
| PPIL3 | 1.060883449 | 9.37E-20 | 4.87E-19 |
| SAA2 | -1.863614097 | 1.92E-06 | 3.04E-06 |
| CAPRIN1 | 1.009894897 | 1.14E-18 | 5.12E-18 |
| CNNM1 | 3.290812824 | 0.022097796 | 0.025540166 |
| CCDC74B | 2.815755002 | 0.000660465 | 0.000873029 |
| RPL23 | 1.130946726 | 5.38E-18 | 2.26E-17 |
| CD151 | 1.355607949 | 1.78E-16 | 6.48E-16 |
| SOX11 | 4.587976927 | 1.13E-08 | 2.11E-08 |
| LYZ | 2.74250725 | 2.09E-07 | 3.60E-07 |
| ARL2 | 1.663955863 | 2.72E-19 | 1.31E-18 |
| EIF3H | 1.259962814 | 1.17E-21 | 7.87E-21 |
| INSC | 2.470845994 | 3.99E-09 | 7.77E-09 |
| HECTD2 | 1.312133774 | 4.29E-12 | 1.03E-11 |
| BEND3 | 1.908887784 | 2.17E-18 | 9.44E-18 |
| BCAS4 | 2.374391184 | 1.58E-17 | 6.38E-17 |
| PHACTR3 | -1.319321191 | 5.20E-25 | 7.47E-24 |
| TYK2 | 1.028318829 | 2.85E-23 | 2.62E-22 |
| RXRB | 1.178768859 | 1.28E-25 | 2.32E-24 |
| NT5C | 1.242622127 | 8.44E-19 | 3.86E-18 |
| SHCBP1 | 4.083711127 | 1.24E-25 | 2.27E-24 |
| RPS14 | 1.001491988 | 1.31E-15 | 4.42E-15 |
| LOXL2 | 2.317139646 | 1.17E-24 | 1.49E-23 |
| FABP6 | 5.416594713 | 4.64E-06 | 7.08E-06 |
| LSM11 | 1.485440045 | 4.67E-22 | 3.36E-21 |
| MAPT | 3.728369377 | 3.34E-25 | 5.12E-24 |
| DLL3 | 5.197557783 | 1.60E-06 | 2.56E-06 |
| PAFAH1B3 | 2.676106759 | 2.23E-19 | 1.08E-18 |
| TNR | -1.069109728 | 3.79E-13 | 1.02E-12 |
| UBE2T | 3.919652977 | 6.28E-29 | 1.16E-26 |
| SIM2 | 1.514224793 | 2.22E-05 | 3.24E-05 |
| SLC26A8 | 1.338732025 | 6.00E-05 | 8.50E-05 |
| ZNF786 | 1.043327904 | 1.00E-19 | 5.15E-19 |
| CAMK2B | -1.020499186 | 3.22E-13 | 8.74E-13 |
| TAF11 | 1.200970727 | 2.44E-26 | 6.15E-25 |
| PPFIA4 | 3.445226561 | 1.52E-08 | 2.82E-08 |
| SLC41A3 | 1.499637306 | 1.20E-27 | 6.68E-26 |
| CDC37 | 1.152095514 | 9.68E-28 | 6.26E-26 |
| MXD4 | 1.183862957 | 1.50E-20 | 8.71E-20 |
| GGA1 | 1.13556259 | 4.87E-24 | 5.40E-23 |
| LYPLAL1 | 1.099293683 | 2.81E-18 | 1.21E-17 |
| RPLP2 | 1.28620473 | 7.65E-19 | 3.54E-18 |
| RAD51 | 3.083486593 | 3.17E-25 | 5.10E-24 |
| GPR63 | 2.284189861 | 9.03E-07 | 1.46E-06 |
| HES4 | 1.889874662 | 1.32E-13 | 3.75E-13 |
| ZMAT5 | 1.153641646 | 1.92E-23 | 1.86E-22 |
| ACLY | 1.72916242 | 4.16E-25 | 6.17E-24 |
| IL1RAPL1 | 2.507360925 | 0.018498847 | 0.021597639 |
| PAQR6 | 2.219052458 | 5.20E-17 | 1.96E-16 |
| RET | -1.524776585 | 2.42E-15 | 7.95E-15 |
| ATP2A1 | 2.243839983 | 1.45E-17 | 5.91E-17 |
| CDKN2AIPNL | 1.617157731 | 4.54E-27 | 1.90E-25 |
| RGS20 | 3.351108563 | 1.57E-12 | 3.94E-12 |
| GJD3 | 1.32579556 | 0.000480186 | 0.000638398 |
| TRPM4 | 1.053285272 | 2.85E-10 | 6.10E-10 |
| FIBP | 1.181233751 | 1.12E-26 | 3.68E-25 |
| GRIN1 | 3.794455245 | 6.27E-08 | 1.10E-07 |
| ADH1B | -1.255601077 | 3.38E-17 | 1.31E-16 |
| KCNQ2 | 2.659480083 | 0.000227683 | 0.00030895 |
| SLC8A2 | 2.767441927 | 7.26E-06 | 1.08E-05 |
| CSNK2B | 1.203898167 | 1.79E-21 | 1.16E-20 |
| PGBD5 | 1.446448471 | 0.004866242 | 0.006025494 |
| DBH | -2.5796475 | 1.78E-24 | 2.15E-23 |
| UTP20 | 1.073140735 | 1.97E-15 | 6.58E-15 |
| NDUFA3 | 1.026477301 | 5.85E-15 | 1.89E-14 |
| SPC25 | 3.876644781 | 4.19E-27 | 1.84E-25 |
| TUBB2B | 1.464381528 | 4.69E-07 | 7.76E-07 |
| PIGU | 1.783745638 | 6.36E-28 | 4.50E-26 |
| IL9R | 1.653043821 | 6.09E-06 | 9.17E-06 |
| CPSF1 | 1.488820768 | 5.96E-26 | 1.19E-24 |
| SLC26A6 | 3.264917543 | 1.25E-29 | 1.15E-26 |
| RANGAP1 | 1.094041645 | 1.45E-14 | 4.49E-14 |
| SLC38A2 | -1.01427383 | 1.82E-12 | 4.47E-12 |
| ABCG1 | 1.219196584 | 6.94E-05 | 9.78E-05 |
| BRMS1 | 1.32587185 | 2.80E-24 | 3.26E-23 |
| IQCA1 | 2.278007448 | 0.040462024 | 0.045956867 |
| ZNF430 | 1.075160066 | 0.001315145 | 0.001706535 |
| TMCO1 | 1.222554776 | 1.23E-27 | 6.68E-26 |
| GNS | 1.024769477 | 3.56E-19 | 1.69E-18 |
| MAP4K2 | 1.373798476 | 1.10E-21 | 7.47E-21 |
| RASSF7 | 1.338459318 | 4.03E-16 | 1.40E-15 |
| MAG | -2.280274115 | 2.50E-06 | 3.93E-06 |
| GAPDH | 1.248414671 | 5.02E-20 | 2.72E-19 |
| SDF2L1 | 1.481545202 | 1.81E-20 | 1.03E-19 |
| LCAT | -2.01751558 | 2.17E-26 | 5.87E-25 |
| PODXL | 2.245678287 | 1.93E-21 | 1.24E-20 |
| GADD45B | -1.378552154 | 6.51E-15 | 2.09E-14 |
| ZC3H3 | 1.781449862 | 2.51E-26 | 6.15E-25 |
| CLCN7 | 1.496307991 | 2.92E-23 | 2.62E-22 |
| STEAP3 | -1.126360647 | 2.11E-19 | 1.04E-18 |
| LAD1 | 1.696933142 | 2.63E-05 | 3.82E-05 |
| STK36 | 1.324664367 | 8.75E-20 | 4.57E-19 |
| UBD | 3.043177952 | 4.29E-18 | 1.82E-17 |
| PSMD4 | 1.708901336 | 2.02E-28 | 2.66E-26 |
| ATOX1 | 1.223423813 | 2.85E-16 | 1.01E-15 |
| ATP6AP1 | 1.4206618 | 4.59E-28 | 4.50E-26 |
| MT1G | -1.932254305 | 3.78E-23 | 3.25E-22 |
| CHST6 | 3.41235401 | 2.37E-12 | 5.77E-12 |
| RUFY4 | 1.790744609 | 1.38E-07 | 2.40E-07 |
| ADH1A | -1.235677643 | 5.65E-17 | 2.11E-16 |
| SLC4A8 | 1.211984891 | 2.75E-05 | 3.98E-05 |
| PUF60 | 1.51359968 | 1.35E-26 | 4.00E-25 |
| ATG9B | 1.052369405 | 5.60E-06 | 8.47E-06 |
| U2AF2 | 1.104236074 | 8.95E-25 | 1.21E-23 |
| CCDC114 | 2.324467063 | 2.35E-12 | 5.74E-12 |
| GPS1 | 1.177243955 | 1.26E-26 | 3.87E-25 |
| CAPNS1 | 1.102139965 | 5.81E-23 | 4.86E-22 |
| MYBPC1 | 4.613389083 | 1.32E-17 | 5.38E-17 |
| SNX32 | 1.372311983 | 2.63E-13 | 7.23E-13 |
| FBL | 1.557454629 | 8.92E-23 | 7.20E-22 |
| TH | -1.302119294 | 7.05E-23 | 5.79E-22 |
| PRKDC | 1.5144719 | 2.87E-20 | 1.59E-19 |
| EIF3K | 1.096469729 | 2.20E-23 | 2.09E-22 |
| HLA-DMA | 1.330972764 | 4.11E-05 | 5.89E-05 |
| ADAM15 | 1.984843174 | 1.42E-26 | 4.02E-25 |
| PELP1 | 1.027738663 | 5.19E-20 | 2.79E-19 |
| RPS11 | 1.057241653 | 2.20E-17 | 8.71E-17 |
| SNRNP70 | 1.446005461 | 5.73E-26 | 1.17E-24 |
| LOXL4 | 1.6540369 | 0.000122753 | 0.000170335 |
| SH2D7 | 1.491318864 | 0.028589139 | 0.03271394 |
| BRIX1 | 1.118035351 | 2.13E-19 | 1.04E-18 |
| FOXD4 | 2.481901913 | 4.40E-13 | 1.17E-12 |
| VIM | 1.039764142 | 1.08E-08 | 2.03E-08 |
| TAGLN2 | 1.520330225 | 1.87E-20 | 1.06E-19 |
| CFL1 | 1.150639413 | 9.42E-25 | 1.26E-23 |
| LRFN2 | 2.972437215 | 7.14E-06 | 1.07E-05 |
| RPL18 | 1.079345052 | 9.79E-15 | 3.07E-14 |
| C1QL4 | 5.117909601 | 6.76E-13 | 1.74E-12 |
| COLQ | 1.15934062 | 5.74E-10 | 1.20E-09 |
| SPDYE1 | 1.276576342 | 2.18E-07 | 3.74E-07 |
| NUP210L | 1.831094209 | 8.10E-08 | 1.42E-07 |
| CLDN18 | 5.29204101 | 4.65E-06 | 7.08E-06 |
| PEA15 | 1.834112264 | 3.57E-27 | 1.64E-25 |
| PBLD | -1.132284629 | 1.55E-17 | 6.28E-17 |
| DHX37 | 1.307897847 | 2.12E-23 | 2.03E-22 |
| TDRD12 | 1.97943111 | 3.50E-08 | 6.30E-08 |
| CSF3 | -2.380770477 | 2.67E-07 | 4.51E-07 |
| NOTCH3 | 2.780411434 | 5.89E-23 | 4.88E-22 |
| MAPK3 | 1.378505311 | 5.73E-26 | 1.17E-24 |
| HBA2 | -1.130704948 | 4.51E-16 | 1.56E-15 |
| SERPINB6 | 1.006754391 | 3.53E-17 | 1.36E-16 |
| COL1A1 | 2.512046056 | 6.53E-07 | 1.07E-06 |
| EFNA1 | 1.424491432 | 2.68E-17 | 1.05E-16 |
| SCRIB | 1.979270154 | 8.37E-27 | 2.96E-25 |
| HPCA | 2.856339686 | 4.29E-07 | 7.13E-07 |
| MED25 | 1.238267789 | 7.68E-25 | 1.07E-23 |
| ZNF765 | 1.050340258 | 9.08E-12 | 2.15E-11 |
| CST2 | 3.857661226 | 3.34E-07 | 5.58E-07 |
| SLC39A14 | -1.00625467 | 4.26E-17 | 1.63E-16 |
| NTNG2 | 1.972839346 | 0.000305274 | 0.000410602 |
| HPX | -1.227427518 | 1.21E-21 | 8.09E-21 |
| NMNAT2 | 3.247242575 | 4.48E-07 | 7.44E-07 |
| BAIAP2L2 | 3.655663842 | 2.44E-19 | 1.18E-18 |
| CSF2RA | 1.00220086 | 0.010499139 | 0.012511928 |
| FBP2 | 1.194230426 | 0.002536832 | 0.003219153 |
| UNC45A | 1.188490577 | 1.13E-24 | 1.46E-23 |
| C11orf49 | 1.323877816 | 3.87E-12 | 9.33E-12 |
| MFGE8 | 1.879663886 | 1.72E-17 | 6.89E-17 |
| CACYBP | 1.748309409 | 6.00E-27 | 2.21E-25 |
| C19orf48 | 1.999520891 | 1.63E-24 | 2.00E-23 |
| RALGAPB | 1.09688874 | 1.78E-19 | 8.90E-19 |
| CAMLG | 1.071725313 | 3.21E-25 | 5.10E-24 |
| MIOX | 4.851295268 | 1.25E-11 | 2.94E-11 |
| HOXA3 | 3.45233503 | 3.81E-11 | 8.71E-11 |
| LSM14B | 1.18794716 | 1.16E-19 | 5.93E-19 |
| E4F1 | 1.392442978 | 3.43E-27 | 1.64E-25 |
| ZNF273 | 1.639008651 | 3.13E-15 | 1.02E-14 |
| ACTL6B | 2.040911566 | 6.72E-07 | 1.10E-06 |
| DNAJB2 | 1.158680835 | 8.45E-24 | 8.94E-23 |
| RNF165 | -1.143399299 | 6.71E-15 | 2.14E-14 |
| CNN1 | 1.49920381 | 5.90E-06 | 8.91E-06 |
| MCM8 | 2.122818328 | 5.53E-22 | 3.94E-21 |
| CD1A | 2.59217383 | 0.001206462 | 0.001576627 |
| CD163 | -1.334933291 | 9.77E-13 | 2.48E-12 |
| CAPN9 | 3.514919618 | 2.09E-11 | 4.89E-11 |
| IKBKG | 1.508548481 | 9.70E-15 | 3.05E-14 |
| GSDMD | 1.262469128 | 2.96E-22 | 2.16E-21 |
| AQP10 | 5.425470636 | 0.005191352 | 0.006393632 |
| PKD1L1 | 1.906361159 | 6.89E-11 | 1.55E-10 |
| ZNF223 | 1.090686274 | 4.00E-09 | 7.77E-09 |
| CALY | 4.625994587 | 4.40E-13 | 1.17E-12 |
| PPP1R16A | 1.706225372 | 4.07E-23 | 3.44E-22 |
| PDGFRL | 2.36573094 | 8.68E-16 | 2.97E-15 |
| FAM19A3 | 1.889961696 | 3.28E-05 | 4.73E-05 |
| BDNF | 1.980832708 | 0.000379029 | 0.000508319 |
| BRD9 | 1.371672511 | 7.64E-26 | 1.49E-24 |
| FAM178B | 5.524525005 | 0.000932614 | 0.001229233 |
| CD99L2 | 1.023655351 | 1.96E-14 | 5.98E-14 |
| ORM1 | -1.304539272 | 3.46E-14 | 1.04E-13 |
| CDK3 | 1.696266824 | 7.66E-14 | 2.21E-13 |
| LCN2 | 5.19036883 | 1.91E-10 | 4.14E-10 |
| DAND5 | 3.714088829 | 4.96E-17 | 1.88E-16 |
| ABCC8 | 3.359152026 | 0.001226241 | 0.001600201 |
| CDC123 | 1.084702494 | 3.34E-25 | 5.12E-24 |
| MRPS12 | 1.347125046 | 2.23E-23 | 2.09E-22 |
| CCT3 | 1.810898487 | 5.25E-29 | 1.16E-26 |
| COL4A1 | 2.445970353 | 1.76E-22 | 1.33E-21 |
| NEDD8 | 1.000631393 | 5.10E-25 | 7.45E-24 |
| C19orf53 | 1.221807363 | 3.78E-23 | 3.25E-22 |
| ZNF385D | 4.423497121 | 2.47E-08 | 4.52E-08 |
| ZNF835 | 1.592620945 | 0.023952233 | 0.027614103 |
| CHRNA1 | 3.377505077 | 0.001288678 | 0.001676922 |
| HOXA9 | 3.365883092 | 1.58E-09 | 3.19E-09 |
| CHRM2 | -3.1847077 | 1.02E-28 | 1.56E-26 |
| ULBP2 | 2.041193177 | 0.001138632 | 0.001490101 |
| CEBPA | 1.298142284 | 2.30E-10 | 4.93E-10 |
| PTOV1 | 1.020748775 | 9.25E-23 | 7.27E-22 |
| TRIM16L | 3.085758402 | 2.21E-13 | 6.13E-13 |
| CHST10 | 1.958818202 | 3.43E-06 | 5.34E-06 |
| SCNN1D | 1.301566287 | 5.83E-10 | 1.21E-09 |
| ABCC1 | 1.751091565 | 3.82E-06 | 5.91E-06 |
| CLEC4G | -4.964773758 | 2.54E-29 | 1.16E-26 |
| RIBC1 | 1.107329914 | 4.22E-12 | 1.01E-11 |
| KLK14 | 1.025683452 | 0.007042075 | 0.008524618 |
| HP | -1.550738951 | 6.60E-21 | 3.92E-20 |
| FBXL6 | 1.721101741 | 2.75E-25 | 4.52E-24 |
| IP6K1 | 1.541470835 | 6.19E-28 | 4.50E-26 |
| IGDCC3 | 6.912104856 | 1.14E-10 | 2.52E-10 |
| ADAMTS16 | 4.457924866 | 1.68E-05 | 2.45E-05 |
| FOXP4 | 1.171554318 | 4.06E-18 | 1.73E-17 |
| ERI3 | 1.053370282 | 2.29E-24 | 2.70E-23 |
| PSORS1C1 | 2.785572966 | 9.85E-13 | 2.49E-12 |
| DDX51 | 1.101603691 | 7.75E-24 | 8.29E-23 |
| SPINK5 | 3.864660742 | 7.88E-17 | 2.91E-16 |
| PARP10 | 1.007382442 | 2.13E-13 | 5.93E-13 |
| TJAP1 | 1.375959572 | 1.02E-25 | 1.91E-24 |
| RRP7A | 1.079300029 | 1.13E-17 | 4.62E-17 |
| NNAT | 1.814527448 | 2.21E-07 | 3.76E-07 |
| TNFRSF18 | 2.561731461 | 9.11E-14 | 2.60E-13 |
| SKA2 | 1.144153089 | 1.67E-18 | 7.37E-18 |
| HM13 | 1.266917329 | 2.68E-26 | 6.32E-25 |
| RPL19 | 1.026824514 | 5.37E-20 | 2.86E-19 |
| CNTD2 | 2.127800457 | 0.002948634 | 0.003711003 |
| SLC44A4 | 1.685031557 | 0.009302802 | 0.011173078 |
| PTGES2 | 1.211906346 | 8.10E-22 | 5.65E-21 |
| RAP1GAP | 1.485443187 | 0.000356667 | 0.000479028 |
| C11orf45 | 1.482852424 | 2.93E-05 | 4.23E-05 |
| RAET1E | 2.626490925 | 3.77E-17 | 1.45E-16 |
| YWHAH | 1.03860783 | 6.81E-18 | 2.83E-17 |
| SLC25A39 | 1.412758208 | 5.70E-28 | 4.50E-26 |
| PLAG1 | 2.434622255 | 7.71E-09 | 1.46E-08 |
| DENR | 1.017453821 | 3.43E-23 | 3.01E-22 |
| CCDC130 | 1.289111658 | 1.08E-24 | 1.42E-23 |
| TSC2 | 1.167342595 | 4.51E-22 | 3.27E-21 |
| CYP1A2 | -2.708234578 | 4.83E-24 | 5.40E-23 |
| EIF3B | 1.24874976 | 2.54E-26 | 6.15E-25 |
| KRTAP5-10 | 2.571477595 | 7.37E-11 | 1.66E-10 |
| IL17RD | 1.608742747 | 0.00531166 | 0.006533058 |
| ASS1 | -1.209277619 | 1.11E-18 | 5.04E-18 |
| PCDHB9 | 1.957504416 | 0.019719488 | 0.022906476 |
| RPL9 | 1.017727107 | 1.66E-13 | 4.68E-13 |
| RPS16 | 1.237336113 | 3.98E-19 | 1.88E-18 |
| SCRT1 | 3.469793926 | 2.23E-12 | 5.46E-12 |
| DTNBP1 | 1.277420931 | 2.19E-16 | 7.86E-16 |
| GAP43 | 3.762870431 | 1.35E-09 | 2.74E-09 |
| PRMT1 | 1.214395535 | 1.04E-21 | 7.09E-21 |
| RPL35 | 1.011796151 | 1.26E-10 | 2.76E-10 |
| CSNK1E | 1.369031189 | 8.20E-22 | 5.67E-21 |
| NRBP2 | 1.54713757 | 6.82E-14 | 1.99E-13 |
| QRFPR | 2.812563932 | 5.31E-06 | 8.05E-06 |
| CYP4A11 | -1.682720458 | 1.82E-24 | 2.18E-23 |
| HOXA2 | 1.155298152 | 0.043421986 | 0.04901623 |
| ZNF695 | 5.570477282 | 6.42E-12 | 1.53E-11 |
| MIIP | 1.311502028 | 6.60E-21 | 3.92E-20 |
| HCN2 | 3.88148167 | 5.41E-16 | 1.86E-15 |
| RAD1 | 1.280279349 | 8.93E-26 | 1.71E-24 |
| OGG1 | 1.396435785 | 7.28E-24 | 7.97E-23 |
| SNRPB | 1.820132541 | 1.04E-26 | 3.53E-25 |
| TSPO | 1.123651952 | 7.10E-06 | 1.06E-05 |
| ALB | -1.271411838 | 6.53E-21 | 3.92E-20 |
| STK31 | 2.011083176 | 0.030622577 | 0.034997231 |
| SEC61A1 | 1.063371792 | 1.38E-22 | 1.07E-21 |
| C2orf66 | 2.193875137 | 3.32E-07 | 5.55E-07 |
| FAIM2 | 1.741856934 | 0.002399684 | 0.00305779 |
| MKI67 | 4.142195733 | 1.44E-26 | 4.02E-25 |
| DBF4B | 2.305002302 | 1.37E-25 | 2.42E-24 |
| PLIN2 | -1.025279189 | 1.68E-12 | 4.18E-12 |
| FTL | 1.012826153 | 7.28E-07 | 1.18E-06 |
| PLG | -1.150948202 | 1.74E-19 | 8.75E-19 |
| NONO | 1.14288616 | 2.66E-22 | 1.97E-21 |
| IL32 | 1.30426765 | 2.19E-07 | 3.75E-07 |
| AIRE | 3.761615359 | 3.97E-08 | 7.13E-08 |
| HEXB | 1.036839629 | 3.86E-21 | 2.38E-20 |
| ADH1C | -1.028374127 | 1.14E-12 | 2.87E-12 |
| DCN | -1.667203784 | 1.56E-19 | 7.91E-19 |
| DMC1 | 3.05148087 | 4.75E-13 | 1.26E-12 |
| AKR1C3 | 2.237927015 | 1.18E-23 | 1.20E-22 |
| ATP1A1 | 1.374696775 | 2.76E-14 | 8.37E-14 |
| APC2 | 1.727783791 | 1.02E-18 | 4.64E-18 |
| ATP13A1 | 1.276050849 | 2.54E-26 | 6.15E-25 |
| LRP4 | 2.404812626 | 0.001343554 | 0.001736053 |
| NEK3 | 1.236146576 | 8.15E-10 | 1.67E-09 |
| TPM1 | 1.132356012 | 7.63E-14 | 2.21E-13 |
| PRDM7 | 4.068049527 | 1.41E-14 | 4.36E-14 |
| RBM39 | 1.022023262 | 1.94E-22 | 1.45E-21 |
| NUCB2 | 1.138133216 | 5.06E-15 | 1.64E-14 |
| SLC39A3 | 1.160306235 | 2.31E-23 | 2.15E-22 |
| NPTX1 | 4.90260008 | 2.63E-06 | 4.14E-06 |
| TSPAN17 | 1.482213159 | 4.30E-26 | 9.41E-25 |
| TTLL10 | 1.819887163 | 8.30E-10 | 1.70E-09 |
| TKT | 2.14621164 | 5.82E-20 | 3.08E-19 |
| ZNF750 | 2.011301438 | 2.15E-08 | 3.96E-08 |
| ALDH2 | -1.118639365 | 1.47E-22 | 1.12E-21 |
| ZSCAN10 | 2.578673801 | 6.98E-06 | 1.05E-05 |
| RPL38 | 1.439218844 | 2.47E-21 | 1.55E-20 |
| ZNF266 | 1.221010666 | 1.30E-10 | 2.84E-10 |
| GABPB2 | 1.244926275 | 7.90E-19 | 3.64E-18 |
| GK5 | 1.371470583 | 2.55E-17 | 1.00E-16 |
| TFAP2A | 2.967708056 | 5.48E-08 | 9.66E-08 |
| NPC2 | 1.04888862 | 1.89E-15 | 6.34E-15 |
| STAB2 | -4.308485282 | 2.35E-28 | 2.70E-26 |
| ZNF554 | 1.062079414 | 1.61E-19 | 8.13E-19 |
| PPM1G | 1.25419811 | 3.04E-27 | 1.55E-25 |
| TM4SF5 | 1.249442242 | 0.000184165 | 0.000251382 |
| DEGS1 | 1.182576048 | 6.59E-18 | 2.76E-17 |
| SNRPD2 | 1.676345913 | 6.68E-25 | 9.45E-24 |
| ACTA2 | 1.250331356 | 4.10E-10 | 8.67E-10 |
| PPFIA2 | 1.380651127 | 0.005499479 | 0.006746028 |
| MED12 | 1.313723275 | 2.94E-18 | 1.26E-17 |
| CLDN6 | 2.731347475 | 0.010853907 | 0.012917975 |
| PROKR1 | 3.210944204 | 0.001918788 | 0.002462043 |
| ARF1 | 1.047819245 | 9.58E-24 | 1.00E-22 |
| BAMBI | 1.233232765 | 0.000177963 | 0.000243278 |
| MYO18B | 7.89485534 | 5.23E-17 | 1.96E-16 |
| HSPB1 | 2.248492077 | 7.65E-24 | 8.28E-23 |
| RAD52 | 1.118448791 | 2.04E-16 | 7.35E-16 |
| ATP6V1B1 | 2.723246385 | 2.15E-11 | 5.01E-11 |
| SLC4A5 | 1.54299924 | 1.81E-08 | 3.34E-08 |
| DUSP23 | 1.262389478 | 7.11E-18 | 2.95E-17 |
| SQSTM1 | 1.73640938 | 9.02E-17 | 3.32E-16 |
| TIAM2 | 1.475282108 | 1.24E-10 | 2.73E-10 |
| CENPB | 1.049599291 | 1.59E-24 | 2.00E-23 |
| ST8SIA5 | 1.911237463 | 5.03E-07 | 8.30E-07 |
| C5orf34 | 3.129868494 | 5.70E-28 | 4.50E-26 |
| EIF5A2 | 2.109171417 | 2.85E-06 | 4.46E-06 |
| RPS19 | 1.458705052 | 4.25E-19 | 2.00E-18 |
| ABLIM2 | 2.112905295 | 3.93E-12 | 9.47E-12 |
| MAP4 | 1.074502071 | 2.07E-21 | 1.32E-20 |
| SUMO2 | 1.02926143 | 8.60E-23 | 7.00E-22 |
| SUSD2 | 1.245359678 | 0.023799713 | 0.027472693 |
| HSP90AA1 | 1.057758216 | 6.55E-19 | 3.04E-18 |
| ITGA5 | 1.287054671 | 6.76E-14 | 1.97E-13 |
| POLR2J3 | 1.232581108 | 3.81E-18 | 1.63E-17 |
| NENF | 1.322306343 | 5.37E-20 | 2.86E-19 |
| CYB5R1 | 1.336324415 | 1.52E-25 | 2.64E-24 |
| ASPSCR1 | 1.849182383 | 9.13E-22 | 6.27E-21 |
| FGB | -1.058014469 | 1.53E-16 | 5.60E-16 |
| FANCD2 | 2.949769142 | 2.65E-25 | 4.43E-24 |
| CXXC1 | 1.076969515 | 1.31E-23 | 1.29E-22 |
| C1R | -1.22900639 | 9.14E-23 | 7.25E-22 |
| TOMM20 | 1.308037669 | 2.55E-25 | 4.34E-24 |
| KRTCAP2 | 1.800577173 | 5.18E-29 | 1.16E-26 |
| GDAP1L1 | 3.802448279 | 3.14E-07 | 5.28E-07 |
| NHLH1 | 1.346572575 | 4.18E-05 | 5.98E-05 |
| RAB3IP | 1.062293468 | 2.78E-09 | 5.48E-09 |
| USP49 | 1.965496002 | 5.93E-27 | 2.21E-25 |
| VWF | 2.050685501 | 2.34E-17 | 9.24E-17 |
| H1F0 | 1.367938608 | 1.00E-19 | 5.15E-19 |
| KCNK12 | 4.372756289 | 3.41E-07 | 5.68E-07 |
| C7orf50 | 1.347324986 | 2.12E-21 | 1.34E-20 |
| ALDOA | 1.811157437 | 2.08E-19 | 1.03E-18 |
| FRMPD2 | 2.987097508 | 0.002161158 | 0.002761479 |
| ACBD7 | 2.348572446 | 4.88E-09 | 9.41E-09 |
| GBX2 | 4.810654416 | 3.11E-10 | 6.65E-10 |
| PLA2G4B | 1.438502375 | 4.96E-08 | 8.78E-08 |
| APOBEC3H | 1.173997369 | 2.21E-07 | 3.76E-07 |
| ZNF207 | 1.174588523 | 3.52E-24 | 3.99E-23 |
| PLCD4 | 1.805931467 | 1.23E-18 | 5.51E-18 |
| KLHDC3 | 1.10065845 | 6.85E-22 | 4.85E-21 |
| HSPA1A | 1.410241863 | 5.02E-12 | 1.20E-11 |
| GALR3 | 1.958779069 | 1.44E-05 | 2.12E-05 |
| NOL7 | 1.300999743 | 1.02E-27 | 6.26E-26 |
| KCNN2 | -2.836152312 | 2.96E-23 | 2.62E-22 |
| C21orf58 | 3.003671143 | 5.54E-27 | 2.21E-25 |
| ATP9A | 1.267198346 | 3.84E-11 | 8.76E-11 |
| MMRN1 | -1.34618788 | 3.57E-12 | 8.65E-12 |
| KAAG1 | 2.549842817 | 3.15E-05 | 4.54E-05 |
| ATP8A2 | 4.133796307 | 3.44E-11 | 7.90E-11 |
| NRM | 2.212587731 | 4.54E-21 | 2.79E-20 |

Abbreviation: HCC, hepatocellular carcinoma

Table S4. The UHSP90AA1-expression level correlated with the clinicopathological features of patients with HCC.

| Characteristic | Low HSP90AA1expression | High HSP90AA1 expression | p |
| --- | --- | --- | --- |
| T stage |  |  | 0.746 |
| T1 | 95 (25.6%) | 88 (23.7%) |  |
| T2 | 46 (12.4%) | 49 (13.2%) |  |
| T3 | 38 (10.2%) | 42 (11.3%) |  |
| T4 | 5 (1.3%) | 8 (2.2%) |  |
| N stage |  |  | 0.624 |
| N0 | 121 (46.9%) | 133 (51.6%) |  |
| N1 | 1 (0.4%) | 3 (1.2%) |  |
| M stage |  |  | 0.355 |
| M0 | 128 (47.1%) | 140 (51.5%) |  |
| M1 | 3 (1.1%) | 1 (0.4%) |  |
| Pathologic stage |  |  | 0.251 |
| Stage I | 90 (25.7%) | 83 (23.7%) |  |
| Stage II | 41 (11.7%) | 46 (13.1%) |  |
| Stage III | 36 (10.3%) | 49 (14%) |  |
| Stage IV | 4 (1.1%) | 1 (0.3%) |  |
| Tumor status |  |  | 0.382 |
| Tumor free | 107 (30.1%) | 95 (26.8%) |  |
| With tumor | 73 (20.6%) | 80 (22.5%) |  |
| Gender |  |  | 0.825 |
| Female | 62 (16.6%) | 59 (15.8%) |  |
| Male | 125 (33.4%) | 128 (34.2%) |  |
| Race |  |  | 0.315 |
| Asian | 73 (20.2%) | 87 (24%) |  |
| Black or African American | 7 (1.9%) | 10 (2.8%) |  |
| White | 98 (27.1%) | 87 (24%) |  |
| Age |  |  | 0.020 |
| <=60 | 77 (20.6%) | 100 (26.8%) |  |
| >60 | 110 (29.5%) | 86 (23.1%) |  |
| Weight |  |  | 0.652 |
| <=70 | 91 (26.3%) | 93 (26.9%) |  |
| >70 | 85 (24.6%) | 77 (22.3%) |  |
| BMI |  |  | 0.470 |
| <=25 | 86 (25.5%) | 91 (27%) |  |
| >25 | 85 (25.2%) | 75 (22.3%) |  |
| Height |  |  | 0.586 |
| < 170 | 99 (29%) | 102 (29.9%) |  |
| >=170 | 74 (21.7%) | 66 (19.4%) |  |
| Residual tumor |  |  | 0.898 |
| R0 | 171 (49.6%) | 156 (45.2%) |  |
| R1 | 8 (2.3%) | 9 (2.6%) |  |
| R2 | 1 (0.3%) | 0 (0%) |  |
| Histologic grade |  |  | 0.054 |
| G1 | 33 (8.9%) | 22 (6%) |  |
| G2 | 92 (24.9%) | 86 (23.3%) |  |
| G3 | 51 (13.8%) | 73 (19.8%) |  |
| G4 | 8 (2.2%) | 4 (1.1%) |  |
| AHTI |  |  | 0.069 |
| None | 73 (30.8%) | 45 (19%) |  |
| Mild | 50 (21.1%) | 51 (21.5%) |  |
| Severe | 7 (3%) | 11 (4.6%) |  |
| AFP (ng/ml) |  |  | 0.220 |
| <=400 | 120 (42.9%) | 95 (33.9%) |  |
| >400 | 30 (10.7%) | 35 (12.5%) |  |
| Albumin(g/dl) |  |  | 0.499 |
| <3.5 | 35 (11.7%) | 34 (11.3%) |  |
| >=3.5 | 130 (43.3%) | 101 (33.7%) |  |
| Prothrombin time |  |  | 0.910 |
| <=4 | 113 (38%) | 95 (32%) |  |
| >4 | 47 (15.8%) | 42 (14.1%) |  |
| Child-Pugh grade |  |  | 0.169 |
| A | 121 (50.2%) | 98 (40.7%) |  |
| B | 8 (3.3%) | 13 (5.4%) |  |
| C | 1 (0.4%) | 0 (0%) |  |
| FIS |  |  | 0.003 |
| 0 | 51 (23.7%) | 24 (11.2%) |  |
| 1/2 | 21 (9.8%) | 10 (4.7%) |  |
| 3/4 | 14 (6.5%) | 14 (6.5%) |  |
| 5/6 | 33 (15.3%) | 48 (22.3%) |  |
| Vascular invasion |  |  | 0.355 |
| No | 113 (35.5%) | 95 (29.9%) |  |
| Yes | 53 (16.7%) | 57 (17.9%) |  |
| OS event |  |  | 0.007 |
| Alive | 135 (36.1%) | 109 (29.1%) |  |
| Dead | 52 (13.9%) | 78 (20.9%) |  |
| DSS event |  |  | 0.185 |
| Alive | 150 (41%) | 137 (37.4%) |  |
| Dead | 34 (9.3%) | 45 (12.3%) |  |
| PFI event |  |  | 1.000 |
| Alive | 95 (25.4%) | 96 (25.7%) |  |
| Dead | 92 (24.6%) | 91 (24.3%) |  |

Abbreviations: AHTI, adjacent hepatic tissue inflammation; FIS, fibrosis ishak score; HCC, hepatocellular carcinoma

Table S5. The LRP4-expression level correlated with the clinicopathological features of patients with HCC.

| Characteristic | Low LRP4 expression | High LRP4 expression | p |
| --- | --- | --- | --- |
| T stage |  |  | 0.013 |
| T1 | 106 (28.6%) | 77 (20.8%) |  |
| T2 | 45 (12.1%) | 50 (13.5%) |  |
| T3 | 31 (8.4%) | 49 (13.2%) |  |
| T4 | 4 (1.1%) | 9 (2.4%) |  |
| N stage |  |  | 1.000 |
| N0 | 125 (48.4%) | 129 (50%) |  |
| N1 | 2 (0.8%) | 2 (0.8%) |  |
| M stage |  |  | 0.365 |
| M0 | 131 (48.2%) | 137 (50.4%) |  |
| M1 | 3 (1.1%) | 1 (0.4%) |  |
| Pathologic stage |  |  | 0.013 |
| Stage I | 100 (28.6%) | 73 (20.9%) |  |
| Stage II | 40 (11.4%) | 47 (13.4%) |  |
| Stage III | 32 (9.1%) | 53 (15.1%) |  |
| Stage IV | 3 (0.9%) | 2 (0.6%) |  |
| Tumor status |  |  | 0.987 |
| Tumor free | 103 (29%) | 99 (27.9%) |  |
| With tumor | 77 (21.7%) | 76 (21.4%) |  |
| Gender |  |  | 0.008 |
| Female | 48 (12.8%) | 73 (19.5%) |  |
| Male | 139 (37.2%) | 114 (30.5%) |  |
| Race |  |  | 0.151 |
| Asian | 81 (22.4%) | 79 (21.8%) |  |
| Black or African American | 12 (3.3%) | 5 (1.4%) |  |
| White | 86 (23.8%) | 99 (27.3%) |  |
| Age |  |  | 0.797 |
| <=60 | 90 (24.1%) | 87 (23.3%) |  |
| >60 | 96 (25.7%) | 100 (26.8%) |  |
| Weight |  |  | 0.126 |
| <=70 | 86 (24.9%) | 98 (28.3%) |  |
| >70 | 90 (26%) | 72 (20.8%) |  |
| BMI |  |  | 0.242 |
| <=25 | 85 (25.2%) | 92 (27.3%) |  |
| >25 | 88 (26.1%) | 72 (21.4%) |  |
| Height |  |  | 0.248 |
| < 170 | 98 (28.7%) | 103 (30.2%) |  |
| >=170 | 78 (22.9%) | 62 (18.2%) |  |
| Residual tumor |  |  | 0.460 |
| R0 | 169 (49%) | 158 (45.8%) |  |
| R1 | 7 (2%) | 10 (2.9%) |  |
| R2 | 1 (0.3%) | 0 (0%) |  |
| Histologic grade |  |  | 0.078 |
| G1 | 32 (8.7%) | 23 (6.2%) |  |
| G2 | 96 (26%) | 82 (22.2%) |  |
| G3 | 51 (13.8%) | 73 (19.8%) |  |
| G4 | 5 (1.4%) | 7 (1.9%) |  |
| AHTI |  |  | 0.048 |
| None | 54 (22.8%) | 64 (27%) |  |
| Mild | 63 (26.6%) | 38 (16%) |  |
| Severe | 10 (4.2%) | 8 (3.4%) |  |
| AFP (ng/ml) |  |  | 0.126 |
| <=400 | 118 (42.1%) | 97 (34.6%) |  |
| >400 | 28 (10%) | 37 (13.2%) |  |
| Albumin(g/dl) |  |  | 0.352 |
| <3.5 | 40 (13.3%) | 29 (9.7%) |  |
| >=3.5 | 117 (39%) | 114 (38%) |  |
| Prothrombin time |  |  | 0.909 |
| <=4 | 106 (35.7%) | 102 (34.3%) |  |
| >4 | 44 (14.8%) | 45 (15.2%) |  |
| Child-Pugh grade |  |  | 0.567 |
| A | 117 (48.5%) | 102 (42.3%) |  |
| B | 13 (5.4%) | 8 (3.3%) |  |
| C | 1 (0.4%) | 0 (0%) |  |
| FIS |  |  | 0.135 |
| 0 | 36 (16.7%) | 39 (18.1%) |  |
| 1/2 | 22 (10.2%) | 9 (4.2%) |  |
| 3/4 | 18 (8.4%) | 10 (4.7%) |  |
| 5/6 | 45 (20.9%) | 36 (16.7%) |  |
| Vascular invasion |  |  | 0.036 |
| No | 116 (36.5%) | 92 (28.9%) |  |
| Yes | 47 (14.8%) | 63 (19.8%) |  |
| OS event |  |  | 0.003 |
| Alive | 136 (36.4%) | 108 (28.9%) |  |
| Dead | 51 (13.6%) | 79 (21.1%) |  |
| DSS event |  |  | 0.114 |
| Alive | 151 (41.3%) | 136 (37.2%) |  |
| Dead | 33 (9%) | 46 (12.6%) |  |
| PFI event |  |  | 0.535 |
| Alive | 99 (26.5%) | 92 (24.6%) |  |
| Dead | 88 (23.5%) | 95 (25.4%) |  |

Abbreviations: AHTI, adjacent hepatic tissue inflammation; FIS, fibrosis ishak score; HCC, hepatocellular carcinoma

Table S6. The PPM1G-expression level correlated with the clinicopathological features of patients with HCC.

| Characteristic | Low PPM1G expression | High PPM1G expression | p |
| --- | --- | --- | --- |
| T stage |  |  | < 0.001 |
| T1 | 111 (29.9%) | 72 (19.4%) |  |
| T2 | 37 (10%) | 58 (15.6%) |  |
| T3 | 33 (8.9%) | 47 (12.7%) |  |
| T4 | 4 (1.1%) | 9 (2.4%) |  |
| N stage |  |  | 0.625 |
| N0 | 120 (46.5%) | 134 (51.9%) |  |
| N1 | 1 (0.4%) | 3 (1.2%) |  |
| M stage |  |  | 1.000 |
| M0 | 122 (44.9%) | 146 (53.7%) |  |
| M1 | 2 (0.7%) | 2 (0.7%) |  |
| Pathologic stage |  |  | < 0.001 |
| Stage I | 103 (29.4%) | 70 (20%) |  |
| Stage II | 35 (10%) | 52 (14.9%) |  |
| Stage III | 31 (8.9%) | 54 (15.4%) |  |
| Stage IV | 3 (0.9%) | 2 (0.6%) |  |
| Tumor status |  |  | 0.154 |
| Tumor free | 109 (30.7%) | 93 (26.2%) |  |
| With tumor | 70 (19.7%) | 83 (23.4%) |  |
| Gender |  |  | 1.000 |
| Female | 60 (16%) | 61 (16.3%) |  |
| Male | 127 (34%) | 126 (33.7%) |  |
| Race |  |  | 0.045 |
| Asian | 67 (18.5%) | 93 (25.7%) |  |
| Black or African American | 10 (2.8%) | 7 (1.9%) |  |
| White | 101 (27.9%) | 84 (23.2%) |  |
| Age |  |  | 0.055 |
| <=60 | 79 (21.2%) | 98 (26.3%) |  |
| >60 | 108 (29%) | 88 (23.6%) |  |
| Weight |  |  | 0.001 |
| <=70 | 76 (22%) | 108 (31.2%) |  |
| >70 | 96 (27.7%) | 66 (19.1%) |  |
| BMI |  |  | 0.007 |
| <=25 | 76 (22.6%) | 101 (30%) |  |
| >25 | 93 (27.6%) | 67 (19.9%) |  |
| Height |  |  | 0.392 |
| < 170 | 97 (28.4%) | 104 (30.5%) |  |
| >=170 | 75 (22%) | 65 (19.1%) |  |
| Residual tumor |  |  | 0.110 |
| R0 | 167 (48.4%) | 160 (46.4%) |  |
| R1 | 5 (1.4%) | 12 (3.5%) |  |
| R2 | 1 (0.3%) | 0 (0%) |  |
| Histologic grade |  |  | 0.014 |
| G1 | 32 (8.7%) | 23 (6.2%) |  |
| G2 | 99 (26.8%) | 79 (21.4%) |  |
| G3 | 51 (13.8%) | 73 (19.8%) |  |
| G4 | 3 (0.8%) | 9 (2.4%) |  |
| AHTI |  |  | 0.681 |
| None | 70 (29.5%) | 48 (20.3%) |  |
| Mild | 54 (22.8%) | 47 (19.8%) |  |
| Severe | 10 (4.2%) | 8 (3.4%) |  |
| AFP (ng/ml) |  |  | < 0.001 |
| <=400 | 125 (44.6%) | 90 (32.1%) |  |
| >400 | 22 (7.9%) | 43 (15.4%) |  |
| Albumin(g/dl) |  |  | 0.245 |
| <3.5 | 33 (11%) | 36 (12%) |  |
| >=3.5 | 131 (43.7%) | 100 (33.3%) |  |
| Prothrombin time |  |  | 0.182 |
| <=4 | 107 (36%) | 101 (34%) |  |
| >4 | 54 (18.2%) | 35 (11.8%) |  |
| Child-Pugh grade |  |  | 0.363 |
| A | 119 (49.4%) | 100 (41.5%) |  |
| B | 9 (3.7%) | 12 (5%) |  |
| C | 1 (0.4%) | 0 (0%) |  |
| FIS |  |  | 0.310 |
| 0 | 45 (20.9%) | 30 (14%) |  |
| 1/2 | 21 (9.8%) | 10 (4.7%) |  |
| 3/4 | 14 (6.5%) | 14 (6.5%) |  |
| 5/6 | 41 (19.1%) | 40 (18.6%) |  |
| Vascular invasion |  |  | 0.355 |
| No | 113 (35.5%) | 95 (29.9%) |  |
| Yes | 53 (16.7%) | 57 (17.9%) |  |
| OS event |  |  | 0.013 |
| Alive | 134 (35.8%) | 110 (29.4%) |  |
| Dead | 53 (14.2%) | 77 (20.6%) |  |
| DSS event |  |  | 0.142 |
| Alive | 149 (40.7%) | 138 (37.7%) |  |
| Dead | 33 (9%) | 46 (12.6%) |  |
| PFI event |  |  | 0.214 |
| Alive | 102 (27.3%) | 89 (23.8%) |  |
| Dead | 85 (22.7%) | 98 (26.2%) |  |

Abbreviations: AHTI, adjacent hepatic tissue inflammation; FIS, fibrosis ishak score; HCC, hepatocellular carcinoma

Table S7. The SEC61A1-expression level correlated with the clinicopathological features of patients with HCC.

| Characteristic | Low SEC61A1 expression | High SEC61A1 expression | p |
| --- | --- | --- | --- |
| T stage |  |  | 0.029 |
| T1 | 105 (28.3%) | 78 (21%) |  |
| T2 | 45 (12.1%) | 50 (13.5%) |  |
| T3 | 31 (8.4%) | 49 (13.2%) |  |
| T4 | 5 (1.3%) | 8 (2.2%) |  |
| N stage |  |  | 0.055 |
| N0 | 132 (51.2%) | 122 (47.3%) |  |
| N1 | 0 (0%) | 4 (1.6%) |  |
| M stage |  |  | 0.365 |
| M0 | 137 (50.4%) | 131 (48.2%) |  |
| M1 | 1 (0.4%) | 3 (1.1%) |  |
| Pathologic stage |  |  | 0.009 |
| Stage I | 100 (28.6%) | 73 (20.9%) |  |
| Stage II | 44 (12.6%) | 43 (12.3%) |  |
| Stage III | 32 (9.1%) | 53 (15.1%) |  |
| Stage IV | 1 (0.3%) | 4 (1.1%) |  |
| Tumor status |  |  | 0.529 |
| Tumor free | 107 (30.1%) | 95 (26.8%) |  |
| With tumor | 75 (21.1%) | 78 (22%) |  |
| Gender |  |  | 0.002 |
| Female | 46 (12.3%) | 75 (20.1%) |  |
| Male | 141 (37.7%) | 112 (29.9%) |  |
| Race |  |  | 0.669 |
| Asian | 83 (22.9%) | 77 (21.3%) |  |
| Black or African American | 7 (1.9%) | 10 (2.8%) |  |
| White | 91 (25.1%) | 94 (26%) |  |
| Age |  |  | 0.277 |
| <=60 | 83 (22.3%) | 94 (25.2%) |  |
| >60 | 104 (27.9%) | 92 (24.7%) |  |
| Weight |  |  | 0.063 |
| <=70 | 85 (24.6%) | 99 (28.6%) |  |
| >70 | 92 (26.6%) | 70 (20.2%) |  |
| BMI |  |  | 0.291 |
| <=25 | 85 (25.2%) | 92 (27.3%) |  |
| >25 | 87 (25.8%) | 73 (21.7%) |  |
| Height |  |  | 0.265 |
| < 170 | 97 (28.4%) | 104 (30.5%) |  |
| >=170 | 77 (22.6%) | 63 (18.5%) |  |
| Residual tumor |  |  | 1.000 |
| R0 | 171 (49.6%) | 156 (45.2%) |  |
| R1 | 9 (2.6%) | 8 (2.3%) |  |
| R2 | 1 (0.3%) | 0 (0%) |  |
| Histologic grade |  |  | 0.050 |
| G1 | 34 (9.2%) | 21 (5.7%) |  |
| G2 | 95 (25.7%) | 83 (22.5%) |  |
| G3 | 53 (14.4%) | 71 (19.2%) |  |
| G4 | 4 (1.1%) | 8 (2.2%) |  |
| AHTI |  |  | 0.062 |
| None | 57 (24.1%) | 61 (25.7%) |  |
| Mild | 55 (23.2%) | 46 (19.4%) |  |
| Severe | 14 (5.9%) | 4 (1.7%) |  |
| AFP (ng/ml) |  |  | 0.510 |
| <=400 | 118 (42.1%) | 97 (34.6%) |  |
| >400 | 32 (11.4%) | 33 (11.8%) |  |
| Albumin(g/dl) |  |  | 0.830 |
| <3.5 | 39 (13%) | 30 (10%) |  |
| >=3.5 | 125 (41.7%) | 106 (35.3%) |  |
| Prothrombin time |  |  | 0.097 |
| <=4 | 117 (39.4%) | 91 (30.6%) |  |
| >4 | 40 (13.5%) | 49 (16.5%) |  |
| Child-Pugh grade |  |  | 0.567 |
| A | 119 (49.4%) | 100 (41.5%) |  |
| B | 10 (4.1%) | 11 (4.6%) |  |
| C | 0 (0%) | 1 (0.4%) |  |
| FIS |  |  | 0.138 |
| 0 | 35 (16.3%) | 40 (18.6%) |  |
| 1/2 | 19 (8.8%) | 12 (5.6%) |  |
| 3/4 | 13 (6%) | 15 (7%) |  |
| 5/6 | 51 (23.7%) | 30 (14%) |  |
| Vascular invasion |  |  | 0.355 |
| No | 113 (35.5%) | 95 (29.9%) |  |
| Yes | 53 (16.7%) | 57 (17.9%) |  |
| OS event |  |  | < 0.001 |
| Alive | 141 (37.7%) | 103 (27.5%) |  |
| Dead | 46 (12.3%) | 84 (22.5%) |  |
| DSS event |  |  | 0.008 |
| Alive | 156 (42.6%) | 131 (35.8%) |  |
| Dead | 29 (7.9%) | 50 (13.7%) |  |
| PFI event |  |  | 0.836 |
| Alive | 94 (25.1%) | 97 (25.9%) |  |
| Dead | 93 (24.9%) | 90 (24.1%) |  |

Abbreviations: AHTI, adjacent hepatic tissue inflammation; FIS, fibrosis ishak score; HCC, hepatocellular carcinoma

Table S8. The SLC41A3-expression level correlated with the clinicopathological features of patients with HCC.

| Characteristic | Low SLC41A3 expression | High SLC41A3 expression | p |
| --- | --- | --- | --- |
| T stage |  |  | 0.188 |
| T1 | 100 (27%) | 83 (22.4%) |  |
| T2 | 42 (11.3%) | 53 (14.3%) |  |
| T3 | 35 (9.4%) | 45 (12.1%) |  |
| T4 | 8 (2.2%) | 5 (1.3%) |  |
| N stage |  |  | 1.000 |
| N0 | 126 (48.8%) | 128 (49.6%) |  |
| N1 | 2 (0.8%) | 2 (0.8%) |  |
| M stage |  |  | 0.622 |
| M0 | 133 (48.9%) | 135 (49.6%) |  |
| M1 | 1 (0.4%) | 3 (1.1%) |  |
| Pathologic stage |  |  | 0.240 |
| Stage I | 95 (27.1%) | 78 (22.3%) |  |
| Stage II | 40 (11.4%) | 47 (13.4%) |  |
| Stage III | 40 (11.4%) | 45 (12.9%) |  |
| Stage IV | 1 (0.3%) | 4 (1.1%) |  |
| Tumor status |  |  | 0.249 |
| Tumor free | 110 (31%) | 92 (25.9%) |  |
| With tumor | 73 (20.6%) | 80 (22.5%) |  |
| Gender |  |  | 0.122 |
| Female | 53 (14.2%) | 68 (18.2%) |  |
| Male | 134 (35.8%) | 119 (31.8%) |  |
| Race |  |  | 0.686 |
| Asian | 78 (21.5%) | 82 (22.7%) |  |
| Black or African American | 7 (1.9%) | 10 (2.8%) |  |
| White | 95 (26.2%) | 90 (24.9%) |  |
| Age |  |  | 0.232 |
| <=60 | 82 (22%) | 95 (25.5%) |  |
| >60 | 104 (27.9%) | 92 (24.7%) |  |
| Weight |  |  | < 0.001 |
| <=70 | 76 (22%) | 108 (31.2%) |  |
| >70 | 101 (29.2%) | 61 (17.6%) |  |
| BMI |  |  | 0.007 |
| <=25 | 77 (22.8%) | 100 (29.7%) |  |
| >25 | 94 (27.9%) | 66 (19.6%) |  |
| Height |  |  | 0.130 |
| < 170 | 94 (27.6%) | 107 (31.4%) |  |
| >=170 | 78 (22.9%) | 62 (18.2%) |  |
| Residual tumor |  |  | 0.063 |
| R0 | 171 (49.6%) | 156 (45.2%) |  |
| R1 | 5 (1.4%) | 12 (3.5%) |  |
| R2 | 0 (0%) | 1 (0.3%) |  |
| Histologic grade |  |  | 0.002 |
| G1 | 38 (10.3%) | 17 (4.6%) |  |
| G2 | 93 (25.2%) | 85 (23%) |  |
| G3 | 51 (13.8%) | 73 (19.8%) |  |
| G4 | 3 (0.8%) | 9 (2.4%) |  |
| AHTI |  |  | 0.174 |
| None | 68 (28.7%) | 50 (21.1%) |  |
| Mild | 48 (20.3%) | 53 (22.4%) |  |
| Severe | 12 (5.1%) | 6 (2.5%) |  |
| AFP (ng/ml) |  |  | 0.014 |
| <=400 | 122 (43.6%) | 93 (33.2%) |  |
| >400 | 25 (8.9%) | 40 (14.3%) |  |
| Albumin(g/dl) |  |  | 0.733 |
| <3.5 | 39 (13%) | 30 (10%) |  |
| >=3.5 | 123 (41%) | 108 (36%) |  |
| Prothrombin time |  |  | 0.069 |
| <=4 | 103 (34.7%) | 105 (35.4%) |  |
| >4 | 55 (18.5%) | 34 (11.4%) |  |
| Child-Pugh grade |  |  | 0.811 |
| A | 119 (49.4%) | 100 (41.5%) |  |
| B | 10 (4.1%) | 11 (4.6%) |  |
| C | 1 (0.4%) | 0 (0%) |  |
| FIS |  |  | 0.695 |
| 0 | 42 (19.5%) | 33 (15.3%) |  |
| 1/2 | 15 (7%) | 16 (7.4%) |  |
| 3/4 | 14 (6.5%) | 14 (6.5%) |  |
| 5/6 | 48 (22.3%) | 33 (15.3%) |  |
| Vascular invasion |  |  | 0.192 |
| No | 112 (35.2%) | 96 (30.2%) |  |
| Yes | 50 (15.7%) | 60 (18.9%) |  |
| OS event |  |  | < 0.001 |
| Alive | 138 (36.9%) | 106 (28.3%) |  |
| Dead | 49 (13.1%) | 81 (21.7%) |  |
| DSS event |  |  | 0.081 |
| Alive | 154 (42.1%) | 133 (36.3%) |  |
| Dead | 33 (9%) | 46 (12.6%) |  |
| PFI event |  |  | 0.836 |
| Alive | 97 (25.9%) | 94 (25.1%) |  |
| Dead | 90 (24.1%) | 93 (24.9%) |  |

Abbreviations: AHTI, adjacent hepatic tissue inflammation; FIS, fibrosis ishak score; HCC, hepatocellular carcinoma
